# Trifunctional Graphene‐Sandwiched Heterojunction‐Embedded Layered Lattice Electrocatalyst for High Performance in Zn‐Air Battery‐Driven Water Splitting

**DOI:** 10.1002/advs.202408869

**Published:** 2024-09-17

**Authors:** Dong Won Kim, Jihoon Kim, Jong Hui Choi, Do Hwan Jung, Jeung Ku Kang

**Affiliations:** ^1^ Department of Materials Science & Engineering and NanoCentury Institute Korea Advanced Institute of Science and Technology 291 Daehak‐ro Yuseong‐gu Daejeon 34141 Republic of Korea

**Keywords:** graphene‐sandwiched structure, heterojunction, internal electric fields, trifunctional electrocatalyst, water splitting, Zn‐air battery

## Abstract

Zn‐air battery (ZAB)‐driven water splitting holds great promise as a next‐generation energy conversion technology, but its large overpotential, low activity, and poor stability for oxygen reduction reaction (ORR), oxygen evolution reaction (OER), and hydrogen evolution reaction (HER) remain obstacles. Here, a trifunctional graphene‐sandwiched, heterojunction‐embedded layered lattice (G‐SHELL) electrocatalyst offering a solution to these challenges are reported. Its hollow core‐layered shell morphology promotes ion transport to Co_3_S_4_ for OER and graphene‐sandwiched MoS_2_ for ORR/HER, while its heterojunction‐induced internal electric fields facilitate electron migration. The structural characteristics of G‐SHELL are thoroughly investigated using X‐ray absorption spectroscopy. Additionally, atomic‐resolution transmission electron microscopy (TEM) images align well with the DFT‐relaxed structures and simulated TEM images, further confirming its structure. It exhibits an approximately threefold smaller ORR charge transfer resistance than Pt/C, a lower OER overpotential and Tafel slope than RuO₂, and excellent HER overpotential and Tafel slope, while outlasting noble metals in terms of durability. Ex situ X‐ray photoelectron spectroscopy analysis under varying potentials by examining the peak shifts and ratios (Co^2+^/Co^3+^ and Mo^4+^/Mo^6+^) elucidates electrocatalytic reaction mechanisms. Furthermore, the ZAB with G‐SHELL outperforms Pt/C+RuO_2_ in terms of energy density (797 Wh kg^−1^) and peak power density (275.8 mW cm^−2^), realizing the ZAB‐driven water splitting.

## Graphical abstract

A trifunctional G‐SHELL catalyst is developed from a zeolitic imidazole framework on graphene oxide, achieving twofold‐lower ORR transfer resistance than Pt/C, and excellent OER/HER overpotential and Tafel slope. Moreover, the Zn‐air battery‐driven water splitting cell with G‐SHELL exhibits high capacity (703 mAh kg^−1^), energy density (797 Wh kg^−1^), and peak power density (275.8 mW cm^−2^), with long‐cycle stability, outperforming the cell with Pt/C+RuO_2_.

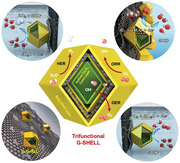

## 1 Introduction

Self‐powered water splitting, which can be driven by high‐energy density cells such as metal‐air batteries without additional energy costs,^[^ ^1^, ^2^, ^3^ ^]^ offers great potential to produce hydrogen, which is the greatest chemical energy carrier (142 MJ kg^−1^).^[^ ^4^ ^]^ Zinc‐air batteries (ZABs) in principle offer a high operation voltage (>1.23 V) for water splitting.^[^ ^5^ ^]^ ZABs can utilize zinc metal as an anode, which has a low redox potential (−0.762 V vs standard hydrogen electrode) for suitably operating in aqueous electrolytes.^[^ ^6^ ^]^ Additionally, the earth‐abundant Zn metal, which allows a high theoretical capacity (820 mAh g^−1^), renders it cost‐effective, environmentally friendly, and flame retardant.^[^ ^7^, ^8^, ^9^ ^]^ During charge, the ZABs cathode undergoes an oxygen evolution reaction (OER), while during discharge, an oxygen reduction reaction (ORR) occurs.^[^ ^10^ ^]^ Because of the abundant supply of oxygen from the air as well as electrons from the Zn metal anode, ZABs can attain high theoretical gravimetric and volumetric energy densities, surpassing Li‐ion batteries by more than 5 folds in gravimetric and 3 folds in volumetric terms.^[^ ^11^, ^12^ ^]^ Nevertheless, typical ZABs exhibit slow oxygen reaction kinetics at the air cathode, resulting in large overpotentials. Besides, the irreversible nature of OER during charge and ORR during discharge at the cathode causes poor cycle stability.^[^ ^13^ ^]^ Furthermore, ZAB‐driven water splitting involves hydrogen evolution reaction (HER), which occurs alongside OER and ORR.^[^ ^14^ ^]^ Pt and its alloys have been postulated as the most active catalysts for ORR and HER,^[^ ^15^ ^]^ whilst noble metal‐oxide catalysts such as RuO_2_ have been considered ideal catalysts for OER.^[^ ^16^ ^]^ However, noble metals have disadvantages such as expensive pricing, limited reserves in the Earth's crust, and low electrocatalytic stability.^[^ ^17^ ^]^ On the other hand, transition metal chalcogenides (TMCs) show significant potential as trifunctional electrocatalysts for ORR, OER, and HER.^[^ ^18^ ^]^ Especially, MoS_2_ is a preferred TMC due to its high theoretical HER activity similar to Pt, and thermodynamic stability.^[^ ^19^, ^20^ ^]^ However, even the most stable 2H (hexagonal) phase of MoS_2_ was reported to have low electrical conductivity, poor wettability, and aggregation properties.^[^ ^21^ ^]^ Furthermore, 2H MoS_2_ showed low activity for OER and ORR, limiting its usefulness as a trifunctional electrocatalyst.^[^ ^22^, ^23^, ^24^ ^]^ Recently, significant advancements have been achieved in the development of heterojunction structures, paring components with different bandgaps, which are capable of enhancing surface reaction kinetics and facilitating charge transfer.^[^ ^25^ ^]^ Especially, the heterojunction of MoS_2_ with multi‐valent 3d transition metals (TMs) has been found to result in improved adsorption of oxidative intermediates such as HO* and HOO*.^[^ ^26^ ^]^ Additionally, heterostructures combining cobalt‐based metallic clusters with MoS_2_ have been reported to reduce the reaction barrier for HER.^[^ ^27^ ^]^ Furthermore, a theoretical study^[^ ^28^ ^]^ suggested that Co‐based TM oxide@MoS_2_ enables a lower OER overpotential than other 3d TMs like V, Cr, and Mn. Furthermore, CoS_x_@Cu_2_MoS_4_‐MoS_2_/NSG, synthesized through pyrolysis, has demonstrated engineered physicochemical properties leading to high activity for OER, ORR, and HER.^[^ ^29^ ^]^ Nevertheless, in practical applications, the discovery and synthesis of a cost‐effective trifunctional electrocatalyst that provides low overpotential, excellent cycle stability, and high activity for ORR, OER, and HER would signify a major breakthrough, enabling the achievement of high performance in a ZAB‐driven water‐splitting cell.

In this work, we synthesize a graphene‐sandwiched, heterojunction‐embedded layered lattice (G‐SHELL) catalyst from a zeolitic imidazole framework (ZIF) on the graphene oxide (GO) surface. G‐SHELL consists of a hollow core‐shell morphology with trifunctional catalytic sites, where it has a hollow Co_3_S_4_ core layer promoting OER activity as well as MoS_2_ shell layers promoting ORR/HER activity. Besides, G‐SHELL is shown to have conductive graphene layers sandwiched between core‐shell heterojunctions, which act as electron conduction channels. Also, a 3D hollow morphology enables fast ion transport, while the layers of MoS_2_ and graphene on the surface promote electron transfer. The density functional theory (DFT) calculations of two heterostructures (Co_3_S_4_/MoS_2_ and graphene/MoS_2_) are also performed to determine whether the formation of these structures is thermodynamically favorable. In addition, Cs‐corrected scanning transmission electron microscopy (Cs‐STEM) as well as X‐ray absorption near edge structure (XANES) and extended X‐ray absorption fine structure (EXAFS) analyses are utilized to elucidate G‐SHELL's heterojunction and bonding characteristics. The experimentally observed STEM images were further compared to the simulated STEM images for verification. Additionally, the induced internal electric fields (IEFs) between heterojunctions are demonstrated to accelerate electron migration to active sites for the three electrocatalytic reactions, causing fast redox kinetics and high activity. Furthermore, the spectra for O 1s, Co^2+^/Co^3+^ 2p, and Mo^6+^/Mo^4+^ 3d orbital peaks are obtained to unveil the reaction mechanisms, and the Mo‐S bonding peak characteristics are measured throughout the extensive potential range to determine electrocatalytic stability. Finally, a self‐powered water‐splitting cell is integrated by combining a rechargeable aqueous ZAB with an alkaline water‐splitting electrolyzer. G‐SHELL acts as an ORR electrocatalyst for the ZAB cathode, converting oxygen from the air into hydroxides during discharge for the alkaline water electrolyzer, while generating oxygen during charge. We show that ZAB‐driven water splitting can achieve high performance by utilizing the full advantages of G‐SHELL as a trifunctional electrocatalyst for ORR, OER, and HER.

## 2 Results and Discussion

**Figure 1:**
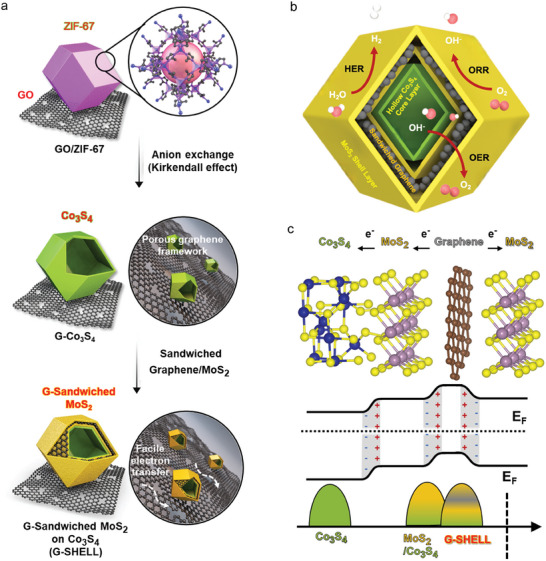
Illustrations of a trifunctional graphene‐sandwiched heterojunction‐embedded layered lattice (G‐SHELL) structure. Schematic representation of a) synthesis procedures of G‐SHELL from a zeolitic imidazole framework, b) hollow core‐layered shell structure with trifunctional sites for oxygen reduction evolution (ORR), oxygen evolution reaction (OER), and hydrogen evolution reaction (HER), and c) heterojunctions, heterojunction‐induced internal electric fields, and the corresponding band structure.

**Figure 2:**
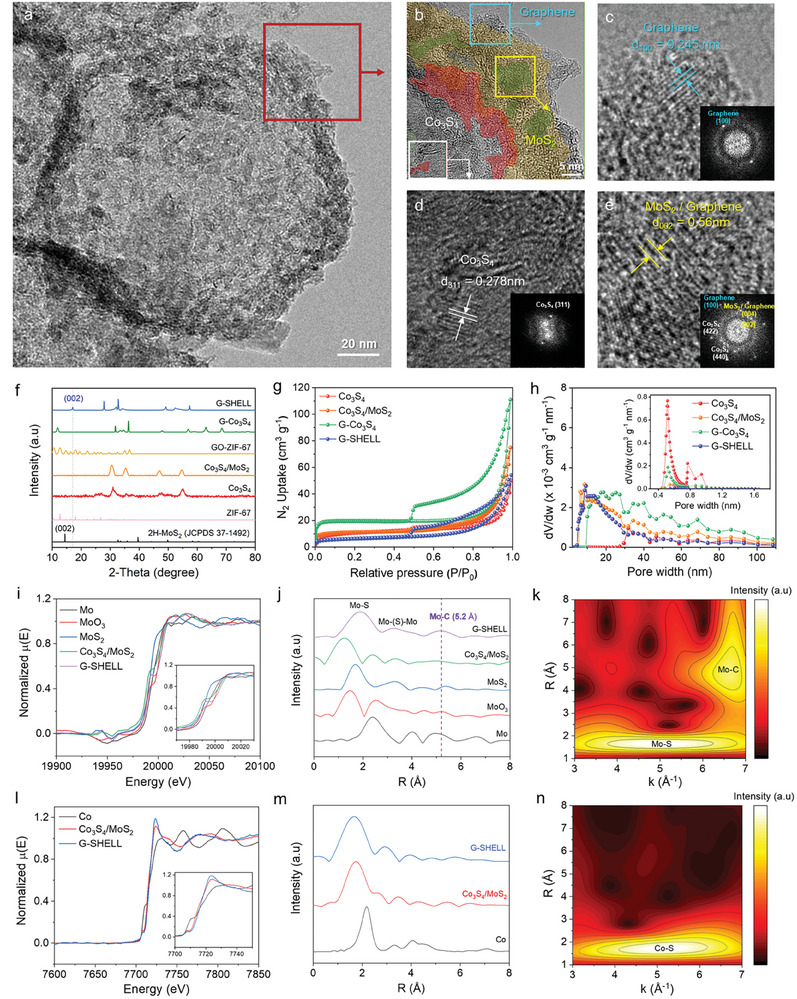
Structural characterizations. a,b) TEM image of G‐SHELL. c–e) HR‐TEM images showing a graphene (100) plane, Co_3_S_4_ (311) plane, and MoS_2_/Graphene (002) plane, respectively. Inset: FFT patterns of the image. f) XRD patterns. g) N_2_ adsorption‐desorption isotherms and its derived, h) differential pore volume curves (Inset: Horvath‐Kawazoe differential pore volume). i,l) XANES spectra. j,m) Fourier transformed real spaces of EXAFS spectra. k,n) Wavelet transformations of k^2^‐weighted Fourier transformations of Mo K and Co K edges, respectively.

**Figure** **1**a depicts the solvothermal synthesis procedure for G‐SHELL. GO was first synthesized according to the method described in our previous work,^[^ ^30^ ^]^ which was modified from Hummer's method.^[^ ^31^ ^]^ Then, the as‐synthesized GO was diluted in methanol and mixed with a cobalt nitrate solution. Next, to grow ZIF‐67 structures on the GO surface, the organic linker of 2‐methylimidazole was added to the Co^2+^/GO mixtures. The ultraviolet‐vis (UV–Vis) and Fourier transform infrared (FT‐IR) spectroscopy analyses were performed to determine the optimal ratios of Co^2+^/GO mixture for ZIF‐67 growth on the GO surface (Figures S1 and S2, Supporting Information). Subsequently, it was mixed with Mo precursor (sodium molybdate) solution and Thioacetamide (TAA) solution. The final mixture was transferred to a Teflon‐lined vessel to undergo the solvothermal synthesis steps. The autoclave was initially put in a pre‐heated 120 °C oven for 4 h to sulfidize ZIF‐67, resulting in Co_3_S_4_ attached to graphene (G‐Co_3_S_4_). The different diffusivities of S and Co were observed to result in the formation of G‐Co_3_S_4_ with a hollow morphology, which is attributed to the Kirkendall effect.^[^ ^32^ ^]^ Subsequently, heating at 200 °C for 8 h was followed to result in the growth of MoS_2_ on the surface of Co_3_S_4_, called Co_3_S_4_‐MoS_2_ heterojunctions. The scanning electron microscopy (SEM) image (Figure S3, Supporting Information) reveals the morphologies of ZIF‐67, Co_3_S_4_, Co_3_S_4_/MoS_2_, GO‐ZIF‐67, G‐Co_3_S_4_, and G‐SHELL. The average particle size distributions (Figure S4, Supporting Information) show that ZIF‐67, Co_3_S_4_, and Co_3_S_4_/MoS_2_ have the large particle sizes of 1100 nm, 721 nm, and 627 nm, respectively, but GO‐ZIF‐67, G‐Co_3_S_4_, and G‐SHELL have the much smaller particle sizes of 138 nm, 122 nm, and 113 nm, respectively. This demonstrates that GO plays an important function in particle size control.^[^ ^33^ ^]^ Interestingly, the catalyst structure derived from ZIF‐67 or GO‐ZIF‐67 was found to retain a rhombic dodecahedral shape, as exhibited in Figures S5–S8 (Supporting Information). Figure 1b and Figure S8 (Supporting Information) reveal how G‐SHELL enables rapid ion transport to both the inner Co_3_S_4_ layer with OER sites and also the outer MoS_2_ layers with ORR/HER sites. The hollow structures of G‐SHELL allow facile ion transport to Co_3_S_4_ and MoS_2_ layers, increasing active site accessibility and decreasing steric hindrance.^[^ ^34^ ^]^ Figure 1c reveals further details on the G‐SHELL band structure. A graphene layer has electron‐donating properties, which implies that the sandwiched graphene donates electrons to its van der Waals (vdW)‐bonded MoS_2_ layer. Then, the MoS_2_ layer transfers electrons to the Co_3_S_4_ layer. Furthermore, the induced heterojunction IEFs promote rapid electron migration to active sites for OER, ORR, and HER, resulting in quick redox kinetics and high activity. **Figure** **2**a reveals the TEM images of a G‐SHELL structure with a hollow core‐shell morphology, in which the inner core looks bright and the outer shell appears dark. An enlarged outer shell structure is also shown in Figure 2b. Besides, high‐resolution TEM (HRTEM) images were analyzed to determine the d‐spacings of 0.245 nm for the graphene (100) plane (Figure 2c),^[^ ^35^ ^]^ 0.278 nm for the Co_3_S_4_ (311) plane (Figure 2d),^[^ ^36^ ^]^ and, 0.56 nm for the graphene‐sandwiched MoS_2_ interlayer (Figure 2e). Because the d‐spacing value of 0.56 nm is not near the MoS_2_ (002) planar distance (0.62 nm) or graphene planar distance (0.33 nm), it is deduced that the (0.56 nm) d‐spacing represents the MoS_2_‐graphene interlayer distance. From the X‐ray diffraction (XRD) analysis, it was revealed that the G‐SHELL's (002) peak at 2θ = 17° (Figure 2f) has the same d_002_ as the d‐spacing value found in HRTEM suggesting MoS_2_‐graphene heterostructure, whereas for the bare 2H‐MoS_2_, the (002) peak has occurred below 2θ = 15° (JCPDS 37–1492),^[^ ^37^ ^]^ indicating a distinct difference in the van der Waals bonding behavior between bare MoS_2_ and G‐SHELL. These graphene‐sandwiched layers were further verified with the (001) peak at 2θ = 9.5°, and the (004) peak at 2θ = 34.2° (Figure S9, Supporting Information). The co‐existence of Co_3_S_4_ and graphene‐sandwiched MoS_2_ was further verified using the fast Fourier transformation (FFT) pattern produced from HRTEM images (Figure 2c–e, inset). Figure 2e verifies the existence of graphene‐sandwiched MoS_2_ interlayers since the d_002_ value is consistent with the XRD peaks.

Furthermore, we conducted DFT simulations to determine the favorability of forming the MoS_2_/Co_3_S_4_ heterostructure and the MoS_2_/graphene heterojunction. The simulated results not only confirmed their existence but also revealed the IEF generated at the Co_3_S_4_/MoS_2_ and MoS_2_/graphene interfaces (Figures S10,S11, Supporting Information). The charge density difference plots demonstrate the electron density donation of MoS_2_ when forming a heterostructure with Co_3_S_4_, and the electron density donation of graphene when forming a heterojunction with MoS_2_, as described in Figure 1c. Further details of DFT calculations are provided in Section 1.5 of the Supporting Information. Using the relaxed Co_3_S_4_/MoS_2_ and MoS_2_/graphene supercells, STEM images were simulated. The simulated STEM images were then compared to the atomic resolution STEM images of G‐SHELL (Figure S12, Supporting Information). The parameter settings for the STEM image simulation were configured to reflect the real microscopy. Bright atoms in Figure S12b,c (Supporting Information) correspond to Mo, while the dark, smaller atoms in Figure S12b (Supporting Information) correspond to carbon. Details about the STEM image calculations are provided in Section 1.6 of the Supporting Information. Figure 2g,h shows the N_2_ adsorption‐desorption isotherms modeled using the Brunauer–Emmett–Teller (BET) theory.^[^ ^38^ ^]^ Micropores in G‐SHELL were identified as having a type IV isotherm with four inflection points. The hysteresis of G‐Co_3_S_4_ and G‐SHELL could be categorized as H3‐type hysteresis because the slopes of their adsorption and desorption curves differed. Given that H3‐type hysteresis occurs in substances containing flaky particles, graphene is concluded to be responsible for the hysteresis behavior. This argument is supported by the isotherm curves from graphene‐containing samples (G‐Co_3_S_4_ and G‐SHELL) differing considerably from graphene‐free samples (Co_3_S_4_ and Co_3_S_4_/MoS_2_). Figure 2h demonstrates that G‐SHELL possesses large ion‐accessible mesopores (ca. 10 nm) enabling rapid ion transport during OER, ORR, and HER. An inductively coupled plasma mass spectrometry (ICP‐MS) analysis shows further that the atomic percentage of Mo in G‐SHELL is much greater than that in the graphene‐free sample, Co_3_S_4_/MoS_2_ (Figure S13, Supporting Information). To reveal the atomic intricacies of the bonding nature around Mo and Co, X‐ray absorption spectroscopy (XAS) analyses (Figure 2i–n), including XANES and EXAFS, were undertaken. Mo, MoO_3_, MoS_2_, Co_3_S_4_/MoS_2,_ and G‐SHELL were investigated to determine the nearby atoms around Mo, whereas Co, Co_3_S_4_/MoS_2_ and G‐SHELL were analyzed to determine the bordering atoms around Co. Compared to the d‐spacing of the bare MoS_2_‐MoS_2_ vdW bond (0.62 nm), the d‐spacing of the MoS_2_‐graphene vdW bond was found to be much shorter (0.56 nm). Because G‐SHELL has more neighboring atoms than bare MoS_2_, it is predicted to have a stronger XANES peak intensity.^[^ ^39^ ^]^ As demonstrated in Figure 2i, the absorption intensity, which is influenced by numerous scatterings in the XANES region, was much greater for G‐SHELL than Co_3_S_4_/MoS_2_. Besides, the existence of the graphene‐MoS_2_ structure was proven by examining the Fourier transformation of k^2^‐weighted EXAFS patterns for the Mo K edge (Figure S14, Supporting Information), the Fourier transformed real space EXAFS spectra (Figure 2j), and the wavelet transformations of k^2^‐weighted Fourier transformations (Figure 2k).^[^ ^40^ ^]^ The Mo─C bond peak at 5.2 Å (Figure 2j) corresponds to both the XRD (002) d‐spacing (Figure 2f) for G‐SHELL and also the HRTEM for MoS_2_‐Graphene (Figure 2e). Also, the EXAFS wavelet transformation using the Morlet wavelet for the Mo K edge of G‐SHELL (Figure 2k) discovered two maximum peaks. One peak at the bottom‐right side corresponds to the Mo─Mo bond, while the other peak at the middle‐right side relates to the Mo─C bond.**^[^** ^41^ **^]^** Other reference samples did not show a signal at 5.2 Å, as verified in Figure S15 (Supporting Information). In addition, the Co K edges of Co, Co_3_S_4_/MoS_2,_ and G‐SHELL were examined using XANES and EXAFS analyses (Figure 2l–n). Similar to the XANES spectrum of Mo, the intensity of G‐SHELL was found to be the greatest, suggesting that the number of scattering around Co was the largest in G‐SHELL ascribed to heterojunctions. The Co K edge EXAFS spectra of Co_3_S_4_/MoS_2_ and G‐SHELL showed similar peak distributions, indicating that Co in G‐SHELL forms a heterojunction.^[^ ^42^ ^]^ Additionally, the wavelet transformation of Co K‐edge EXAFS for G‐SHELL in Figure 2n and Figures S16,S17 (Supporting Information) shows a distinct Co‐S peak, indicating that Co forms a bond with S. For the quantitative analysis of coordination environment around both Mo and Co, EXAFS fitting was conducted for all the measured samples (Figures S18–S20, Supporting Information). Scattering paths for the EXAFS fitting were selected using the as‐relaxed structures of MoS_2_/Graphene and Co_3_S_4_/MoS_2_, and their fitting results including each path's coordination number, R (in Å), bond disorder, and the R‐score of each EXAFS fit are displayed in Tables S1,S2 (Supporting Information). The resulting coordination number of the nearest Mo‐S path for MoS_2_, Co_3_S_4_/MoS_2_, and G‐SHELL were 6.075, 5.980, and 6.030, respectively. We can infer that when Co_3_S_4_ and MoS_2_ form a heterostructure, the two phases can share sulfur atoms, resulting in a decrease of Mo─S bonds.^[^ ^42^ ^]^ Since the MoS_2_ in G‐SHELL not only forms a heterojunction with Co_3_S_4_ but also with graphene, the decrease in Mo‐S coordination number due to sulfur sharing is smaller than that of Co_3_S_4_/MoS_2_, thus the order of Mo─S bond coordination number is MoS_2_ > G‐SHELL > Co_3_S_4_. The same trend is observed for the coordination number of Co‐S in Co_3_S_4_ (4.017) and G‐SHELL (4.061), since sharing sulfur with Mo will result in the decrease of Co‐S coordination number. Furthermore, the coordination number of the Mo‐C scattering was detected to be ca. 10 at R = 4.67 Å, implying the existence of van der Waals bonding between MoS_2_ and graphene.

**Figure 3:**
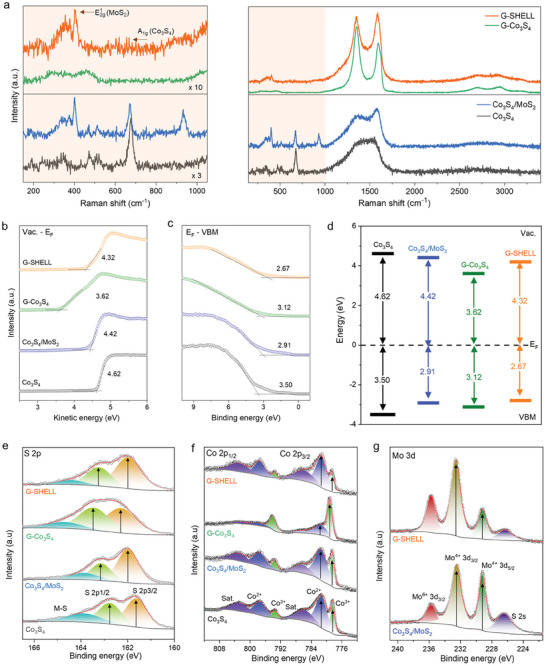
Chemical bonding and electrical property characterizations. a) Raman spectra. b) UPS spectra with the secondary electron cutoff region for work function determination and c) VBM determination. d) Diagram of energy levels calculated through the linear extrapolation of the leading edge in the UPS. XPS spectra of e) S 2p, f) Co 2p, and g) Mo 3d.

**Figure** **3**a shows the Raman spectra for G‐SHELL and other catalysts. The peaks reflected the bonding vibration in G‐SHELL comprised of Co_3_S_4_ and MoS_2_. The peaks of Co_3_S_4_ were detected at 185 cm^−1^ (A_g_), 341 cm^−1^ (E_g_), 464 cm^−1^ (F_2g_), 510 cm^−1^ (S‐S), and 660 cm^−1^ (A_1g_).^[^ ^43^, ^44^ ^]^ Co_3_S_4_/MoS_2_ heterojunction caused a blue shift in the symmetric stretching vibration of the tetrahedral Co^2+^─S bond, suggesting a strong Co^2+^–S interaction and the peak at 960 cm^−1^ is due to the Co‐MoS_x_ heterostructure.^[^ ^45^ ^]^ MoS_2_ exhibited two distinct peaks: in‐plane E^1^_2g_ (ca. 380 cm^−1^) and out‐of‐plane A_1g_ (ca. 404.9 cm^−1^). G‐SHELL showed a peak of 28.54 cm^−1^ between E^1^_2g_ and A_1g_, which is larger than 26.67 cm^−1^ of Co_3_S_4_/MoS_2_, indicating the graphene‐sandwiched MoS_2_ structure via van der Waals (vdW)^[^ ^46^ ^]^ interaction. Furthermore, the I_D_/I_G_ intensity ratios of 1.34 for G‐Co_3_S_4_, and 0.96 for G‐SHELL were used to quantify the degree of graphitization. The lower intensity of G‐SHELL compared to G‐Co_3_S_4_ is ascribed to the increase of graphitic carbons generated by solvothermal synthesis at a higher temperature (200 °C). Furthermore, the ultraviolet photoelectron spectroscopy (UPS) spectra, as seen in Figure 3b–d and Figure S21 (Supporting Information), show that the valence band maximum (VBM) edges are at 3.50 eV for Co_3_S_4_, 2.91 eV for Co_3_S_4_/MoS_2_, 3.12 eV for G‐Co_3_S_4_, and 2.67 eV for G‐SHELL. All of the materials had VBM values less than the Fermi energy level, implying that they were all semiconductors. In addition, the work function, which is defined as the minimum amount of energy necessary for an electron to escape from the Fermi level to the vacuum level,^[^ ^47^ ^]^ was determined. The work functions of Co_3_S_4_, Co_3_S_4_/MoS_2_, G‐Co_3_S_4_, and G‐SHELL were 4.62 eV, 4.42 eV, 3.62 eV, and 4.32 eV, respectively, demonstrating that electrons can readily flow from MoS_2_/graphene to Co_3_S_4_. Because of the high degree of overlap in energy levels between Co_3_S_4_ and MoS_2_/graphene, a built‐up IEF stimulates electron flow from MoS_2_/graphene to Co_3_S_4_ to achieve equilibrium, signaling that it can play a role in accelerating reaction kinetics.^[^ ^48^ ^]^ Furthermore, the X‐ray photoelectron spectroscopy (XPS) survey scan of G‐SHELL in Figure S22 (Supporting Information) identified the peaks of C, Co, Mo, S, and O species. Figure 3e shows three main peaks, indicating the presence of both bridging S_2_^2−^ (denoted as M‐S in the spectrum, including Co–S, Mo–S, and edge sulfur) and also terminal S_2_^2−^ (S 2p_3/2_ and S 2p_1/2_), at 164.1, 162.9, and 161.7 eV.^[^ ^49^ ^]^ Figure S23a (Supporting Information) reveals that the S 2p_3/2_ peak shifted to higher binding energy from 161.2 to 162.0 and 162.3 eV, then slightly decreased to 161.95 eV in Co_3_S_4_, Co_3_S_4_/MoS_2_, G‐Co_3_S_4_, and G‐SHELL. Co_3_S_4_/MoS_2_ and G‐SHELL were found to have comparable binding energies with a similar portion of the S 2p_3/2_ ratio. This indicates that the terminal S species are more strongly bonded to graphene than to MoS_2_, and G‐SHELL has a sandwiched structure. Figure 3f depicts Co 2p spectra containing the two pairs of satellite peaks (778.5 and 793.3 eV) and spin‐orbit doublets (781.4 and 797.5 eV) for Co^3+^ 2p_3/2_ and Co^3+^ 2p_1/2_ as well as Co^2+^ 2p_3/2_ and Co^2+^ 2p_1/2_. The coexistence of the tetrahedral (Co^2+^) and octahedral (Co^3+^) sites is explained by the chemical states^[^ ^50^ ^]^ of these two ions in Co_3_S_4_. Figure S23b (Supporting Information) reveals the shifts in Co_3_S_4_/MoS_2_ (+0.5 eV), G‐Co_3_S_4_ (+0.7 eV), and G‐SHELL (+0.45 eV) compared to the Co^2+^ peak position in Co_3_S_4_. This finding indicates that the heterojunction enhances the binding energy of Co^2+^ species. The bonding nature was further explained by determining the areal ratio of Co^2+^ and Co^3+^ XPS peaks (Co^2+^/Co^3+^). Interestingly, the samples containing Co_3_S_4_/MoS_2_ heterojunctions (Co_3_S_4_/MoS_2_ or G‐SHELL) had a high Co^2+^/Co^3+^ ratio. In contrast, G‐Co_3_S_4_ demonstrated a dramatically lower Co^2+^/Co^3+^ ratio attributed to the bonding between Co_3_S_4_ and graphene surfaces. The high Co^2+^/Co^3+^ ratio in G‐SHELL indicates a shift in Fermi level to attain optimal binding energy with intermediate species (e.g., OH_ads_), which may lead to enhanced OER characteristics and overcome slow ORR kinetics.^[^ ^51^ ^]^ Figure 3g and Figure S23c (Supporting Information) show a comparison of the Mo 3d spectra for Co_3_S_4_/MoS_2_ and G‐SHELL, which have the usual peaks of Mo 3d_5/2_ (228.8 eV), Mo 3d_3/2_ (231.8 eV), and S 2s (226.0 eV).^[^ ^52^ ^]^ Unlike Mo^4+^ 3d_5/2_ and Mo^4+^ 3d_3/2_ peaks that represent MoS_2_, the peak at 235.75 eV reveals a high valence state of Mo^6+^ 3d_3/2_. When compared to Co_3_S_4_/MoS_2_, G‐SHELL has a greater Mo^6+^ peak intensity at the higher position. This considerable rise in the Mo valence state confirms the establishment of a heterojunction between MoS_2_ and Co_3_S_4_, as well as a shift of electron density.^[^ ^53^ ^]^ The deconvoluted C 1s peak in Figure S24 (Supporting Information) shows the characteristics of sp^2^‐hybridized C─C/C═C (284.6 eV), C─S (ca. 285.3 eV), C─O (ca. 286.3 eV), C═S (ca. 287.8 eV) and C═O (ca. 289.1 eV) bonds.^[^ ^54^ ^]^ This indicates that the high sp^2^‐dominance of G‐SHELL contributes to its high electric conductivity.

**Figure 4:**
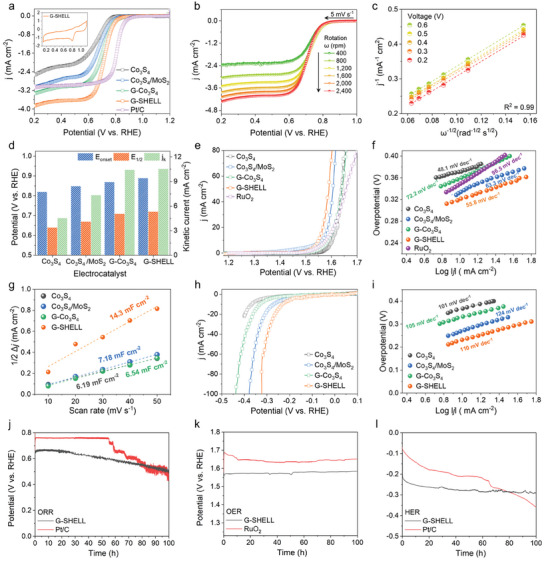
Trifunctional activity of Co_3_S_4_, Co_3_S_4_/MoS_2_, G‐Co_3_S_4_, and G‐SHELL electrocatalysts. a) ORR polarization curves at 1600 rpm in O_2_‐saturated 0.1 M KOH solution. The inset shows the cyclic voltammetry (CV) curves of G‐SHELL in an O_2_‐saturated 0.1 M KOH solution. b) ORR polarization curves of G‐SHELL at various rotating rates in rpm. c) K‐L plots (*ω* ^−1/2^ vs *j* ^−1^; ω and j are the angular rotation speed and the current density at a certain voltage) obtained from the ORR polarization curves of G‐SHELL. d) ORR performance histograms of the onset voltage (E_onset_), and half‐wave potential (E_1/2_), and kinetic current density (j_k_). e) OER polarization curves at 1600 rpm in 1 M KOH and f) corresponding Tafel slopes. g) Electric double layer capacitances calculated from the slope of 1/2Δ*j*‐scan rate plot. (Δ*j* is the current density difference between anodic and cathodic currents at 1.025 V versus reversible hydrogen electrode (RHE) in Figure S22, Supporting Information). h) HER polarization curves at 1600 rpm in 1 M KOH and i) corresponding Tafel slopes. Chronopotentiometry‐based stability test of G‐SHELL and noble metal electrocatalyst for j) ORR, k) OER, and l) HER.

Cyclic voltammetry (CV) measurements were performed in 0.1 M KOH under saturated O_2_ conditions without rotation in a three‐electrode rotating disk electrode (RDE) device. All electrochemical catalysts had a prominent ORR characteristic peak around 0.8 V, as seen in **Figure** **4**a inset and Figure S25 (Supporting Information). Figure 4b also shows the ORR polarization curves acquired through the linear sweep voltammetry measurements on the G‐SHELL catalyst at various rotating speeds. In addition, Figure 4c and Figure S26 (Supporting Information) show Koutecky–Levich (K‐L) plots^[^ ^55^ ^]^ originated from the polarization curves for Co_3_S_4_, Co_3_S_4_/MoS_2_, G‐Co_3_S_4_, and Pt/C. The reversed square root of the rotation speed and the reverse current density were found to be well linearized in K–L plots. Figure 4d and Table S3 (Supporting Information) reveal the onset potential (E_onset_), half‐wave potential (E_1/2_), and kinetic current density (j_k_). G‐SHELL outperformed Co_3_S_4_, Co_3_S_4_/MoS_2_, and G‐Co_3_S_4_ with values of 0.89 V, 0.72 V, and 10.5 mA cm^−2^, which were similar to the benchmark Pt/C (0.93 V, 0.81 V, and 10.7 mA cm^−2^). Figure S27 (Supporting Information) shows the rotating ring‐disk electrode (RRDE) analysis for the detection and quantification of hydrogen peroxide (HO_2_^−^) formation via the 2e^−^ transfer mechanism of the ORR, and it indicates that the ring current density of G‐SHELL was the lowest among our samples with only 2.53% HO_2_^−^ formation. The electron transfer number during ORR for G‐SHELL was found to be 3.95, suggesting a desirable four‐electron route at 0.6 V versus reversible hydrogen electrode (RHE), whereas those of Co_3_S_4_, Co_3_S_4_/MoS_2_, G‐Co_3_S_4,_ and Pt/C were 3.83, 3.88, 3.92, and 3.97, respectively. The ORR kinetics of G‐SHELL were further validated by electrochemical impedance spectroscopy (EIS) analysis at 0.63 V versus RHE at 1600 rpm. The obtained Nyquist plot was fitted to an equivalent circuit model (Figure S28, supporting information), and the parameters are summarized in Table S4 (Supporting Information). G‐SHELL shows the smallest solution resistance (R_s_ = 85.6 Ω) and about three times lower charge transfer resistance (R_ct_ = 290.8 Ω) than that of Pt/C (R_s_ = 100.4 Ω and R_ct_ = 903.6 Ω), demonstrating the efficient redox kinetics facilitated by G‐SHELL's tuned electronic structure. The diffusion‐limited current density (j_L_) of G‐SHELL was 3.9 mA cm^−2^, outperforming the benchmark Pt/C (3.0 mA cm^−2^) at 0.2 V versus reversible hydrogen electrode (RHE) at 1600 rpm. We also evaluated G‐SHELL's OER and HER activities in 1 M KOH electrolyte. Figure 4e demonstrates that G‐SHELL has high OER activity with an overpotential of 320 mV at 10 mA cm^−2^, compared to 354 mV for the noble RuO_2_ catalyst. G‐SHELL exhibits a Tafel slope of 55.8 mV dec^−1^ (Figure 4f), supporting faster OER kinetics than Co_3_S_4_/MoS_2_ (63.3 mV dec^−1^) and RuO_2_ (96.5 mV dec^−1^). Additionally, we measured the double‐layer capacitance (C_dl_), which can be used to calculate the electrochemical active surface area (ECSA).^[^ ^56^ ^]^ It is worth noting that catalytic activity is proportional to the number of active sites related to the ECSA. The C_dl_ of G‐SHELL was determined using CV curves (Figure S29, Supporting information) at various scan rates within a non‐Faradaic potential window (0.925–1.025 V vs RHE). Figure 4g and Figure S30 (Supporting information) show that G‐SHELL has a higher C_dl_ value of 14.3 mF cm^−2^ than Co_3_S_4_ (6.19 mF cm^−2^), Co_3_S_4_/MoS_2_ (6.54 mF cm^−2^), G‐Co_3_S_4_ (7.18 mF cm^−2^), Pt/C (4.24 mF cm^−2^), and RuO_2_ (6.76 mF cm^−2^). G‐SHELL enabled high HER activity in 1 M KOH electrolyte with a low overpotential of 220 mV at 10 mA cm^−2^ and a low Tafel slope of 110 mV dec^−1^, as exhibited in Figure 4h,i and Figure S31 (Supporting information). Table S5 (Supporting Information) reveals that G‐SHELL has excellent overpotentials and Tafel slopes for both HER and OER. To further evaluate the trifunctional activities, the current densities for ORR, OER, and HER, along with the corresponding specific and mass activities, were calculated (Figure S32 and Table S6, Supporting Information). The specific activity and mass activity of G‐SHELL for ORR were determined to be 0.35 A m^−2^ and 7.8 A g^−1^, respectively, which are higher than those of Co_3_S_4_ (0.04 A m^−2^ and 3.5 A g^−1^). Additionally, G‐SHELL exhibited significantly higher OER activities (7.78 A m^−2^, 173.3 A g^−1^) and HER activities (2.25 A m^−2^, 50.1 A g^−1^) compared to Co_3_S_4_ (0.23 A m^−2^ and 18.9 A g^−1^ for OER and 0.07 A m^−2^ and 5.9 A g^−1^ for HER). These superior values were attributed to G‐SHELL's high catalytic activity at specific reaction sites with enhanced intrinsic properties. Besides, G‐SHELL gives the highest stability in both chronoamperometry (Figure S33, Supporting information) and chronopotentiometry (Figure 4I,j), outlasting Pt/C for ORR, RuO_2_ for OER, and Pt/C for HER in terms of durability. Even after being immersed in 1 M KOH for more than 10 days, Figure S34 (Supporting Information) exhibits that G‐SHELL retained its hollow morphology and crystallinity. Additionally, even after 100 hours of three different electrochemical reactions (ORR, OER, HER), the MoS_2_/Graphene sandwiched structures are observed in STEM, matching with the as‐simulated structures of MoS_2_/Graphene sandwiched structure (Figures S35–S37, Supporting Information). SEM‐EDS further confirms the stability of G‐SHELL for three reactions (Figure S38, Supporting Information) without significant structure decomposition. Figure S39 (Supporting Information) demonstrates that G‐SHELL exhibited high methanol tolerance, whereas Pt/C resulted in a significant decline in performance. Although several reports have discussed the surface reconstruction of transition metal‐based catalysts during electrochemical reactions,^[^ ^57^, ^58^ ^]^ the stability of G‐SHELL can be primarily attributed to the formation of Co_3_S_4_/MoS_2_/Graphene heterojunction.^[^ ^59^ ^]^

**Figure 5:**
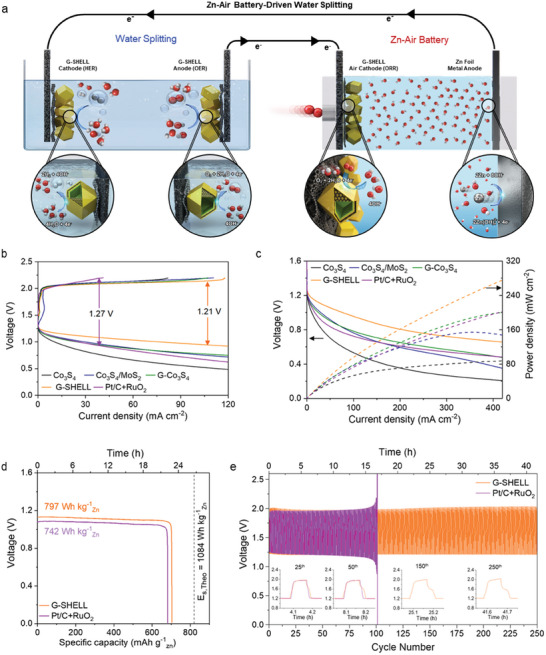
Electrochemical performance of a ZAB‐driven water‐splitting cell with G‐SHELL. a) Diagram of a self‐driven water‐splitting cell integrated by combining a ZAB with an alkaline water electrolyzer. b) Polarization curves during charging and discharging at 10 mV s^−1^ and c) discharge current densities versus voltages with the corresponding power densities. d) Discharge curves under the continuous consumption of zinc metal. The specific capacity was normalized to the mass of the consumed Zn. e) Galvanostatic charge‐discharge cycling profiles of ZAB‐driven water‐splitting cells with G‐SHELL and Pt/C+RuO_2_ at 1 mA cm^−2^ with the interval of 10 min.

A self‐powered water‐splitting cell was developed by combining a ZAB with an alkaline water‐splitting electrolyzer, which requires an OER electrocatalyst at the ZAB cathode to convert oxygen into hydroxide molecules during discharge, the electrolyzer anode with an OER electrocatalyst connected to the ZAB cathode, and the electrolyzer cathode with a HER electrocatalyst connected to the ZAB anode. **Figure** **5**a depicts a ZAB‐driven water‐splitting cell with G‐SHELL as a trifunctional electrocatalyst for OER, HER, and ORR. A reference catalyst Pt/C+RuO_2_ was also employed for the comparison of ZAB performances. Figure S40 (Supporting Information) shows that the open‐circuit voltage (OCV) of 1.43 V in a ZAB cell with G‐SHELL exceeds the OCV of 1.41 V in a ZAB cell with Pt/C+RuO_2_ configuration. Besides, the OCV with G‐SHELL was observed to be sustained over 187 h, outperforming Co_3_S_4_/MoS_2_ (176 h), G‐Co_3_S_4_ (183 h), and Co_3_S_4_ (0.65 h). Furthermore, the decreased potential gap between discharge and charge polarization curves (Figure 5b) demonstrates the G‐SHELL‐based ZAB cell's outstanding rechargeability. Figure 5c shows the discharge polarization and power density curves. The peak power density of the G‐SHELL‐based ZAB cell was 275.8 mW cm^−2^, which was substantially greater than the 202.6 mW cm^−2^ of the Pt/C+RuO_2_‐based cell. As demonstrated in Figure S41 (Supporting Information) and Table S7 (Supporting Information), the round‐trip efficiencies with G‐SHELL varied from 67% at 1.0 mA cm^−2^ to 56% at 20 mA cm^−2^, excelling that of Pt/C+RuO_2_ (from 63% to 37%). The G‐SHELL‐based cell showed a specific capacity of 703 mAh g^−1^ (Figure 5d), outperforming the PtC+RuO_2_‐based cell (681 mAh g^−1^), which makes it more comparable to the theoretical capacity of Zn‐air battery (820 mAh g^−1^).^[^ ^60^ ^]^ Table S8 (Supporting Information) summarizes that the ZAB cell with G‐SHELL leads to a high energy density of 797 Wh kg^−1^ surpassing 742 Wh kg^−1^ with Pt/C as well as those with other electrocatalysts. Figure S42 (Supporting Information) also reveals the rate capability of ZABs with the air electrodes of G‐SHELL and Pt/C+RuO_2_. The G‐SHELL‐based ZAB resulted in stable discharge voltages at even up to 100 mA cm^−2^ under repeated discharge and resting cycles. Besides, the long‐term cycle stability test of ZABs at 10 mA cm^−2^ with 20‐minute intervals was carried out, as shown in Figure S43 (Supporting Information). A small voltage gap of 0.95 V was maintained during 100 cycles with a high round‐trip efficiency of 54.7%, which outperformed the Pt/C+RuO_2_‐based cell (1.12 V, 50.1%). The G‐SHELL‐based cell also demonstrated negligible performance degradation after 250 cycles of operation (41.6 h), while the Pt/C+RuO_2_‐based cell caused a rapid degradation by elevating the voltage gap to 1.156 V after 100 cycles, as exhibited in Figure 5e. Moreover, the contact angle measurements using a catalyst ink drop on a hydrophobic gas diffusion layer with deionized water (DI)‐water droplet on Co_3_S_4_, Co_3_S_4_/MoS_2_, G‐Co_3_S_4_, and G‐SHELL show that the high hydrophilicity of G‐SHELL results in lower surface energy between the electrode and electrolyte (Figure S44, Supporting Information), indicating that the hydrophilic property of G‐SHELL is beneficial for reaction intermediate adsorption and bubble elimination.^[^ ^61^ ^]^ Also, the overall water‐splitting performances of Co_3_S_4_, Co_3_S_4_/MoS_2,_ G‐Co_3_S_4_, G‐SHELL, Pt/C, and RuO_2_, which were coated on a hydrophilic carbon substrate in N_2_‐saturated 1 M KOH solution, suggest that the water electrolyzer required a small overpotential of 0.56 V to achieve a current density of 10 mA cm^−2^, as shown in Figure S45a,b (Supporting Information). In addition, the EIS measurement at 1.56 V versus RHE on the G‐SHELL shows a low charge transfer resistance of 3.32 Ω compared to RuO_2_ (4.64 Ω), indicating facile OER kinetics (Figure S45c and Table S9, Supporting Information). Moreover, the water‐splitting performance in a symmetric two‐electrode system was evaluated in a 1 M KOH solution. The anode and cathode were separated by a Nafion membrane (Figure S46a, Supporting Information). The cell voltage required to achieve 100 mA cm⁻^2^ was 2.07 V for the G‐SHELL∥G‐SHELL configuration, only 90 mV higher than the benchmark water‐splitting system of Pt/C∥RuO₂ (Figure S46b, Supporting Information). Notably, the overall water splitting overpotential of the G‐SHELL∥G‐SHELL configuration was lower than that of Pt/C∥RuO₂ in high current densities. G‐SHELL exhibited a voltage of 2.14 V at 200 mA cm⁻^2^, which is 80 mV lower than the noble metal system. To evaluate the Faraday efficiency of G‐SHELL, the water‐displacement method was employed at 2.1 V for 100 minutes. The experimentally collected H₂ and O₂ gases closely matched with the theoretically calculated amounts, reaching the theoretical value of 2:1 ratio (production rate of 1.17 mmol h⁻¹ for H₂ and 0.56 mmol h⁻¹ for O₂) with Faraday efficiencies of 96.1% for H₂ and 92.7% for O₂ (Figure S46c, Supporting Information). Figure S47a (Supporting Information) depicts a self‐powered hydrogen generation system based on the ZAB‐driven water‐splitting cell, with G‐SHELL serving as the ZAB cathode electrocatalyst for ORR, the electrolyzer anode electrocatalyst for OER, and the electrolyzer cathode electrocatalyst for HER. Besides, a snapshot during the electrolysis process (Figure S47b, Supporting Information) demonstrates that the ZAB‐driven cell produced H_2_ and O_2_ bubbles via HER and OER on the electrolyzer cathode and anode surfaces, respectively.

**Figure 6:**
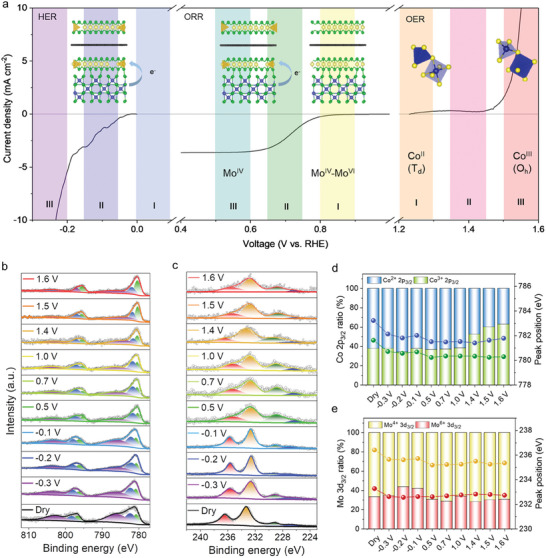
Trifunctional electrocatalytic reaction mechanisms of G‐SHELL. a) Linear sweep voltammetry curve of G‐SHELL that highlights the three different reaction regions. Ex situ XPS spectra in the highlighted regions for b) Co 2p and c) Mo 3d orbitals. Corresponding XPS peak shift and ratio for d) Co 2p_3/2_ and e) Mo 3d_3/2_ orbitals.

Ex situ XPS spectra acquired from the electrodes with a fluorine‐doped tin oxide (FTO) substrate over the OER, ORR, and HER potential regions are depicted in **Figures** **6** and S48 (Supporting Information), which demonstrate the mechanisms of the trifunctional electrocatalyst. Figure 6a suggests that the IEF between Co_3_S_4_ and MoS_2_/graphene accelerates electron migration for fast redox kinetics, by lowering reaction barriers^[^ ^62^, ^63^ ^]^ at HO* sites for OER, O* sites for ORR, and H* sites for HER. Figure S49a (Supporting Information) reveals O 1s spectra with varying potentials. During OER, the peak has shifted to the M‐O binding energy region, whereas the dominant peak has shifted toward the M‐OH binding energy region during ORR and HER. As shown in Figure 6b,c, as the potential increases in the OER region, the area of high‐valency peaks from Co 2p and Mo 3d increases, whereas the area decreases as the potential decreases in the ORR and HER regions. Moreover, the Co^3+^/Co^2+^ ratio was found to dramatically rise during OER (Figure 6d). The high‐valence Co species provide OER active sites, indicating that the local bonding environment of Co_3_S_4_ has altered to allow Co^2+^ species to easily lose their electrons and turn into catalytically active Co^3+^ species. It is worth noting that the Co^2+^ ions strongly bind with the intermediate (HO*) while Co^3+^ ions are capable of releasing O_2_, making the coexistence of the two different states beneficial for the OER activity.^[^ ^64^ ^]^ Additionally, multivalent Mo^6+^ and Mo^4+^ states may serve as active sites, facilitating the formation of OH^−^ during ORR and H_2_ during HER.^[^ ^65^ ^]^ These mechanisms demonstrate that G‐SHELL's trifunctional activity stems from multivalent states, as shown in Figure 6e. The peak position of Mo 3d has remained relatively constant, indicative of a well‐maintained MoS_2_/graphene layer and supporting that G‐SHELL enables high electrochemical stability. Figure S49b (Supporting Information) demonstrates further that the M‐S bonding peak in the S 2p spectrum was stable throughout a wide potential range, indicating remarkable electrocatalytic stability.

## 3 Conclusion

In summary, we developed a trifunctional G‐SHELL electrocatalyst, which is a heterojunction‐embedded layered metal chalcogenide produced on graphene. G‐SHELL was discovered to feature a hollow core‐shell structure with a Co_3_S_4_ inner layer which is active for OER and a MoS_2_/graphene outer layer responsible for ORR/HER activity. Intriguingly, we found that during our synthesis procedure, a substantial amount of MoS_2_ layers form the graphene‐sandwiched heterojunction which enhances electron conductivity and contributes to the stability of Co_3_S_4_. Moreover, the heterojunction‐induced IEF accelerated electron migration to HO* sites for OER, O* sites for ORR, and H* sites for HER. G‐SHELL achieved about 3‐fold smaller ORR resistance than Pt/C, a lower OER overpotential (320 mV at 10 mA cm^−2^) and Tafel slope (55.8 mV dec^−1^) than RuO_2_ at 354 mV and 96.5 mV dec^−1^, and excellent HER overpotential and Tafel slope, while outlasting Pt/C for ORR, RuO_2_ for OER, and Pt/C for HER in terms of durability. Additionally, the stable Mo^6+^/Mo^4+^ ratio and Mo peak location under the applied voltage confirmed G‐SHELL's remarkable stability. The ZAB cell with G‐SHELL surpassed the corresponding cell with Pt/C+RuO_2_ configuration in terms of capacity (703 mAh kg^−1^), energy density (797 Wh kg^−1^), and peak power density (275.8 mW cm^−2^). Furthermore, the G‐SHELL‐based cell enabled negligible performance degradation after 250 cycles, while the Pt/C+RuO_2_‐based cell degraded rapidly only after 100 cycles. Additionally, it exhibited a small voltage gap during cycles as well as a higher round‐trip efficiency than the Pt/C+RuO_2_‐based cell. Moreover, a self‐powered water‐splitting cell was assembled by coupling a rechargeable aqueous ZAB with an alkaline water‐splitting electrolyzer. Consequently, this work provides a strategy to realize a high‐performance trifunctional electrocatalyst capable of achieving low overpotentials, high activity, and long‐cycle stability in ZAB‐driven water splitting.

## Conflict of Interest

The authors declare no conflict of interest.

## Supporting information



Supporting Information

## Data Availability

The data that support the findings of this study are available from the corresponding author upon reasonable request.
