# Supplementary material for: Trifunctional Graphene‐Sandwiched Heterojunction‐Embedded Layered Lattice Electrocatalyst for High Performance in Zn‐Air Battery‐Driven Water Splitting
Source: Adv Sci (Weinh). 2024 Sep 17;11(42):2408869. doi: 10.1002/advs.202408869 (PMC11558083; doi:10.1002/advs.202408869)
Supplement: Supplementary file 1 — Supporting Information [file ADVS-11-2408869-s001.pdf]

ADVANCED SCIENCE
Open Access

# Supporting Information

for *Adv. Sci.*, DOI 10.1002/advs.202408869

Trifunctional Graphene-Sandwiched Heterojunction-Embedded Layered Lattice Electrocatalyst for High Performance in Zn-Air Battery-Driven Water Splitting

*Dong Won Kim, Jihoon Kim, Jong Hui Choi, Do Hwan Jung and Jeung Ku Kang**

Copyright WILEY-VCH Verlag GmbH & Co. KGaA, 69469 Weinheim, Germany, 2024.

# Supporting Information

**Trifunctional Graphene-Sandwiched Heterojunction-Embedded Layered Lattice Electrocatalyst for High Performance in Zn-air Battery-Driven Water Splitting**

*Dong Won Kim*[‡]*, Jihoon Kim*[‡]*, Jong Hui Choi, Do Hwan Jung and Jeung Ku Kang*[*]

D. W. Kim, J. Kim, J. H. Choi, D. H. Jung, Prof. J. K. Kang

Department of Materials Science & Engineering and NanoCentury Institute

Korea Advanced Institute of Science and Technology, 291 Daehak-ro, Yuseong-gu, Daejeon 34141, Republic of Korea

[‡]D. W. Kim and J. Kim contributed equally to this work

[*]Corresponding author: jeungku@kaist.ac.kr

**Section S1. Methods details**

**S1.1. Preparation**

**Chemicals:** Graphite powder as 300 mesh size was obtained from Alfa Aesar Co. Ltd., sulfuric acid, phosphoric acid, hydrogen peroxide, and hydrochloric acid were purchased from Duksan Co. Ltd., Ketjenblack® EC was supplied by Mitsubishi Co. Ltd., fluorine-doped tin oxide (FTO) glass, Nafion™ solution, 2-Methylimidazole, Potassium permanganate, Cobalt(II) nitrate hexahydrate, Sodium molybdate dihydrate, Thioacetamide, Potassium hydroxide, Zinc acetate dihydrate, Zinc Foil, Isopropanol, Anhydrous Ethanol, and Anhydrous Methanol were supplied by Sigma-Aldrich. Deionized water was prepared in the laboratory.

**Synthesis of graphene oxide (GO) solution:** The GO was synthesized by a modified Hummer's method,[1] which is reported in our previous work.[2] 2 g of graphite powder and 40 mL of concentrated sulfuric acid ($H_2SO_4$) were mixed and sonicated for 4 h for pretreatment. Then, graphite powder was washed with deionized water and dried in a vacuum oven at 60 °C overnight. The dried powder was added to 200 mL of concentrated $H_2SO_4$ (95%) and 36 mL of phosphoric acid ($H_3PO_4$, 85%). 12 g of potassium permanganate ($KMnO_4$) was slowly added to the mixture while stirring and the temperature was kept below 100 °C. After mixing, the mixture was stirred for 24 h, and deionized water was carefully added to dilute the acidity. The mixture was kept in an ice bath throughout this process to keep the temperature below 50 °C. The mixture was additionally stirred for 24 h. Subsequently, 30 mL of hydrogen peroxide ($H_2O_2$, 37%) was added to the reaction mixture and then stirred for 24 h. The addition of hydrogen peroxide changed the color of the mixture from dark brown to a bubbly light yellow. The mixture was washed with a 0.1 M hydrochloric acid (HCl) aqueous solution twice to get rid of metal ions. Afterward, the mixture was washed with deionized water 3 times to remove the remaining traces of acid. Then, the mixture was sonicated for 4 h for exfoliation and centrifuged

to remove any heavy particles. The concentration of the mixture was controlled at 4 mg $mL^{-1}$ and stored under ambient conditions.

**Synthesis of ZIF-67 and GO-ZIF-67:** ZIF-67 was synthesized by modifying a previous process[3]. First, 1 mmol of cobalt nitrate hexahydrate ($Co(NO_3)_2 \cdot 6H_2O$) and 3 mmol of 2-Methylimiazole were dissolved in 10mL methanol, Then vigorous stirring until power fully dissolved to clear solution. Then, the solution of 2-Methlylimidazole was added to the cobalt nitrate hexahydrate solution. As a mixture of the two solutions, the purple precipitates were formed. GO-ZIF-67 was prepared by following the aforementioned procedure, except for using a certain amount of the graphene oxide-dispersed methanol solution. The ZIF-67 and GO-ZIF-67 were centrifuged washed with methanol 3 times and dried under a vacuum oven for overnight.

**Synthesis of $Co_3S_4$, $Co_3S_4/MoS_2$, G-$Co_3S_4$, and G-SHELL:** Preparing $Co_3S_4$ and G-$Co_3S_4$, 25mg of ZIF-67 and ZIF-67/GO were dispersed 10mL anhydrous ethanol and stirred until being homogeneously dispersed. Then, separately prepared 25mg of Thioacetamide dissolved in 10mL of ethanol solution were added into ZIF-67 and GO-ZIF-67 solution were mixed and stirred for 30 min. Then, the mixture solution was transferred into a 25 mL Teflon-lined stainless autoclave. The mixture was sealed and heated at 120 °C for 4 h. A Black-colored power was recovered by washing with deionized water and ethanol multiple times with centrifuge. The obtained power was dried in a vacuum oven for overnight. $Co_3S_4/MoS_2$ and G-SHELL followed the similar process by adding 25 mg of Sodium molybdate dihydrate and further heating to 200°C for 8 h to heterogeneously grow $MoS_2$.

## S1.2. Material Characterization

The powder X-ray diffraction (XRD) analysis were done using Ultima IV (Rigaku) with Cu-K1α radiation of 1200 W (40 kV, 30 mA). The range of angle (2θ) was set to be 5-80° at

ambient temperature. Scanning electron microscopy (SEM) analysis were carried out using Magellan400 (FEI company). Transmission electron microscopy (TEM) images were obtained from JEM-ARM200F (JEOL), which an operation voltage of 200 kV. Elemental distributions were examined using the energy-dispersive X-ray microscopy of the same TEM device. The Inductive Coupled Plasma Mass Spectroscopy (ICP-MS) were conducted by using iCAP RQ (Thermo Scientific). For X-ray photoelectron spectroscopy (XPS) analysis, Thermo VG Scientific K-alpha spectrometer (Thermo Scientific) with Al-Kα radiation at 350 W (3 mA) was used. $N_2$ adsorption isotherm data were collected using Qudrasorp (Quantachrome). Pore size distribution was analyzed using the density functional theory method. X-ray absorption at near edge structure (XANES) and extended X-ray absorption fine structure (EXAFS) analyses were performed with QuantumLeap-H2000 (Sigray). The energy ranges of 7650-7950 eV and 19950-20150 eV were selected to analyze Co and Mo, respectively.

### S1.3. Electrochemical Measurements

The electrochemical measurements were conducted using the SP-300 potentiostats (BioLogic). To measure oxygen reduction reaction (ORR), A standard three-electrode electrochemical cell was constructed using rotating disk electrode (RDE) for working electrode (geometric area = 0.0707 $cm^2$), Pt wire for counter electrode, and Hg/HgO electrode as reference electrode, respectively. 0.1 M KOH was used as electrolyte for the analysis of ORR. Catalyst ink was prepared by dispersing 4 mg of catalyst and 1 mg of Ketjen black (EC 600 JD) in a solution containing 950 μL of isopropyl alcohol (IPA) and 50 μL of 5 wt% Nafion solution. The mixed solution was sonicated for over than 60 min for homogeneous dispersion. Afterward, 4 μL of catalyst ink was dropped onto the RDE surface and dried. The measured voltage was converted to a reversible hydrogen electrode (RHE) using the following equation of

$$\mathrm{E(vs.\,RHE)} = E(\mathrm{vs.\,Hg/HgO}) + E^0(\mathrm{vs.\,Hg/HgO}) + 0.0592\ \mathrm{pH} \qquad \mathrm{(S1)}$$

The electrolyte was purged with pure $O_2$ gas for over 30 min. Linear sweep voltammetry (LSV) analysis was conducted at a scan rate of 5 mV $s^{-1}$. The electrochemical active surface area (ESCA) was calculated using the double-layer capacitance ($C_{dl}$) determined using the different scan rates of cyclic voltammetry (CV). The electrochemical impedance spectroscopy (EIS) was conducted at set potential with an AC amplitude of 5 mV and frequency range of 100 kHz to 0.1 Hz. The measured impedance data were fitted using an equivalent circuit model, including $R_s$ (solution resistance), $R_{ct}$ (charge transfer resistance), $R_m$ (mass transfer resistance), and CPE (constant phase element).[4,5] The LSV curves on RDE with different rotating speeds at 400, 800, 1200, 1600, 2000, and 2400 revolutions per minute (rpm) were obtained during the ORR test. The corresponding kinetic current density was calculated based on the Koutecky-Levich (K-L) equation[6] of

$$\frac{1}{J} = \frac{1}{J_L} + \frac{1}{J_K} = \frac{1}{B\omega^{1/2}} + \frac{1}{J_K} \quad \text{(S2)}$$

$$\mathrm{B} = 0.62\mathrm{n}FC_0(D_0)^{2/3}v^{-1/6} \quad \text{(S3)}$$

Where $J_L$ is the diffusion-limited current density, $J_K$ is the kinetic current density, $\omega$ is the angular velocity, n is the transfer number of electrons $F$ is the Faraday constant (96,485 C $mol^{-1}$), $D_0$ is the diffusion coefficient of $O_2$ (1.9 x $10^{-5}$ $cm^2$ $s^{-1}$), $C_0$ is the concentration of $O_2$ (1.2 x $10^{-6}$ mol $cm^{-3}$), and $v$ is the viscosity of the electrolyte (assumed to be 0.01 $cm^2$ $s^{-1}$). The methanol tolerance tests were estimated at a rotation speed of 400 rpm, where 3M methanol was injected at 1000 s. To further investigate the ORR mechanism and kinetics simultaneously changed potential, the Rotating-ring disk electrode (RRDE) measurement was conducted by using an RRDE electrode, consisting of a glassy carbon disk (diameter: 4 mm, A = 0.1256 $cm^2$) surrounded by a Pt ring (inner and outer-ring diameter: 5, 7mm) with dropping 10 μL of catalyst ink. The LSV measurements were performed on an RRDE at a rotation speed of 1600 rpm with a scan rate of 5 mV $s^{-1}$ in an $O_2$-saturated 0.1 M KOH solution. The Pt ring electrode was held

at 1.2 V vs. RHE to estimate the electron transfer number ($n$) per $O_2$ molecule and percentage of peroxide (% $HO_2^-$ ) generation from ring and disk current using the following equation:

$$n = 4 \times \frac{I_D}{I_D + I_R/N} \quad \text{(S4)}$$

$$HO_2^- \ (\%) = 200 \times \frac{I_R/N}{I_D + I_R/N} \quad \text{(S5)}$$

Where $I_D$ and $I_R$ are the disk and ring currents, respectively. $N$ is the collection efficiency of Pt ring which is 0.37. Oxygen evolution reaction (OER) and hydrogen evolution reaction (HER) were conducted at a rotation speed of 1600 rpm and a scan rate of 5 mV $s^{-1}$ in $N_2$-saturated alkaline media of 1 M KOH. The overpotential ($\eta$) of OER and ORR were calculated by $\eta$ = $E_{RHE}$ – 1.23 (V) and HER was $\eta$ = $E_{RHE}$ – 0 (V). LSV curves were corrected by 85% iR compensation. The mass activity ($\mathrm{MA} = \mathrm{I/m}$, A $g^{-1}$) was calculated to catalyst loading mass (m) on GC RDE electrode to observed current (I) in LSV curves at 1600 rpm. which is. The specific activity ($\mathrm{SA} = \mathrm{I/(m\ S_{BET})}$, $\mathrm{A\ m^{-2}}$) was calculated using the BET specific surface area ($S_{BET}$). The stabilities of the catalysts were evaluated using chronoamperometry and chronopotentiometry in 0.1 M KOH for ORR and 1 M KOH for OER and HER. The chronoamperometry measurements were conducted using an RDE electrode at 400 rpm for ORR (0.60 V vs. RHE), OER (1.56 V vs. RHE), and HER (-0.23 V vs. RHE). The chronopotentiometry measurements were performed using a hydrophilic carbon paper current collector for ORR (5 mA $cm^{-2}$), OER (10 mA $cm^{-2}$), and HER (10 mA $cm^{-2}$).The faradaic efficiency (FE) of G-SHELL was calculated by assembling two symmetric electrode (loading 1 mg $cm^{-2}$), using water-gas displacement method to dividing the amount of the experimentally measured gas by the theoretically calculated gas during water electrolysis. The faradaic efficiency was calculated according to the following equation:

$$\mathrm{FE}(\%) = \frac{V_E}{V_T} = \frac{V_E}{(Q \times V_m)/(Z \times F)} \times 100 \quad \text{(S6)}$$

Where $V_E$ is the volume of the experimentally measured gas, $V_T$ is the theoretically calculated volume of gas, $Z$ is the stoichiometric charge number (2 electrons per $H_2$ molecule and 4 electrons per $O_2$ molecule ), $Q = \int_0^{\Delta t} I(t)dt$ is the charge, F is the Faraday constant ($F =$ 96485.34 C mol$^{-1}$), and $V_m$ is the molar volume of gas (24.1 mol L$^{-1}$ at 298.15 K and 1 atm). The *ex-situ* XPS was measured with three different reaction regions by dropping 50 μL of catalyst ink on a conductive FTO glass (2 x 1 cm$^2$, surface resistance of ~7 Ω/sq). The catalyst-loaded FTO glass was assembled in an H-type three-electrode system as the working electrode, with Pt as the counter electrode, and Hg/HgO reference electrode in $N_2$-saturated 1 M KOH for OER. A glassy carbon electrode was used as the counter electrode for HER and ORR (with $O_2$-saturated 0.1 M KOH). Subsequently, LSV was applied with a scan rate of 5 mV s$^{-1}$ to reach the target potential and maintained for 5 minutes under a certain potential. After the reaction, the samples were gently washed with DI water and dried in a room-temperature vacuum oven.

### S1.4. Zn-air battery (ZAB) performance evaluation

A polished and washed Zn plate (250 μm thick) was used as an anode. The catalyst ink was dropped onto a of carbon paper with a Polytetrafluoroethylene coated gas-diffusion layer (Sigracet 39BB) and used as cathode. The mass loading of the catalyst was 1 mg cm$^{-2}$. An aqueous solution containing 6 M KOH and 0.2 M zinc acetate dihydrate [$Zn(OAc)_2 \cdot 2H_2O$] was used as an electrolyte for the ZAB. Ni foam was washed with 3 M HCl and distilled water and used as a backing layer to collect current and support the gas diffusion layer. For the measurement of power density, charging process was initially conducted from open circuit voltage (OCV) to 2.2 V with a scan rate of 10 mV s$^{-1}$, and the discharge process was conducted with the same scan rate until the current density have reached 420 mA cm$^{-2}$. For comparison

of the performance, 20 wt% Pt/C + $RuO_2$ with a mass ratio of 1:1 were used as a reference. The specific capacity of the ZAB was calculated based on the amount of Zn consumed.

**S1.5. Methods for density functional theory (DFT) calculation**

The ab initio calculations were conducted using the Quantum ESPRESSO package for the structural examination of $Co_3S_4/MoS_2$ heterojunction and $MoS_2$-graphene heterostructure. For the exchange-correlation functional, the Perdew-Burke-Ernzerhof exchange correlation functional was used. The cutoff energy used was 400 eV and the force threshold was 0.005 eV bohr$^{-1}$. The Monkhorst-Pack grid was set to $1 \times 1 \times 1$ for both calculations. To avoid undesired interactions along the c-axis during the structural relaxation of $MoS_2/Co_3S_4$ heterojunction, a vacuum of 15 Å was introduced. To accurately consider the Van der Waals interaction between $MoS_2$ and graphene, Grimme-d3 (DFT-D3) correction was applied for the structural relaxation of the $MoS_2$-graphene sandwiched heterostructure. Consequently, the size of the slab was $9.40 \times 9.40 \times 30.89$ Å for $Co_3S_4/MoS_2$ heterojunction and $12.39 \times 12.39 \times 9.85$ Å for $MoS_2$/graphene. The self-consistent calculations satisfied an energy threshold of $10^{-4}$ eV.

**S1.6. STEM image simulation**

For the simulation of a STEM image, the as-obtained cif file of the $MoS_2$/graphene structure was used. All the simulation procedure was conducted using the DrProbe software, and the parameters used for the simulation were the same as the parameters from Thermo Fisher Spectra Ultra. The size of the simulated image was set to 1 nm $\times$ 1 nm.

**Section S2. Supplementary figures and tables**

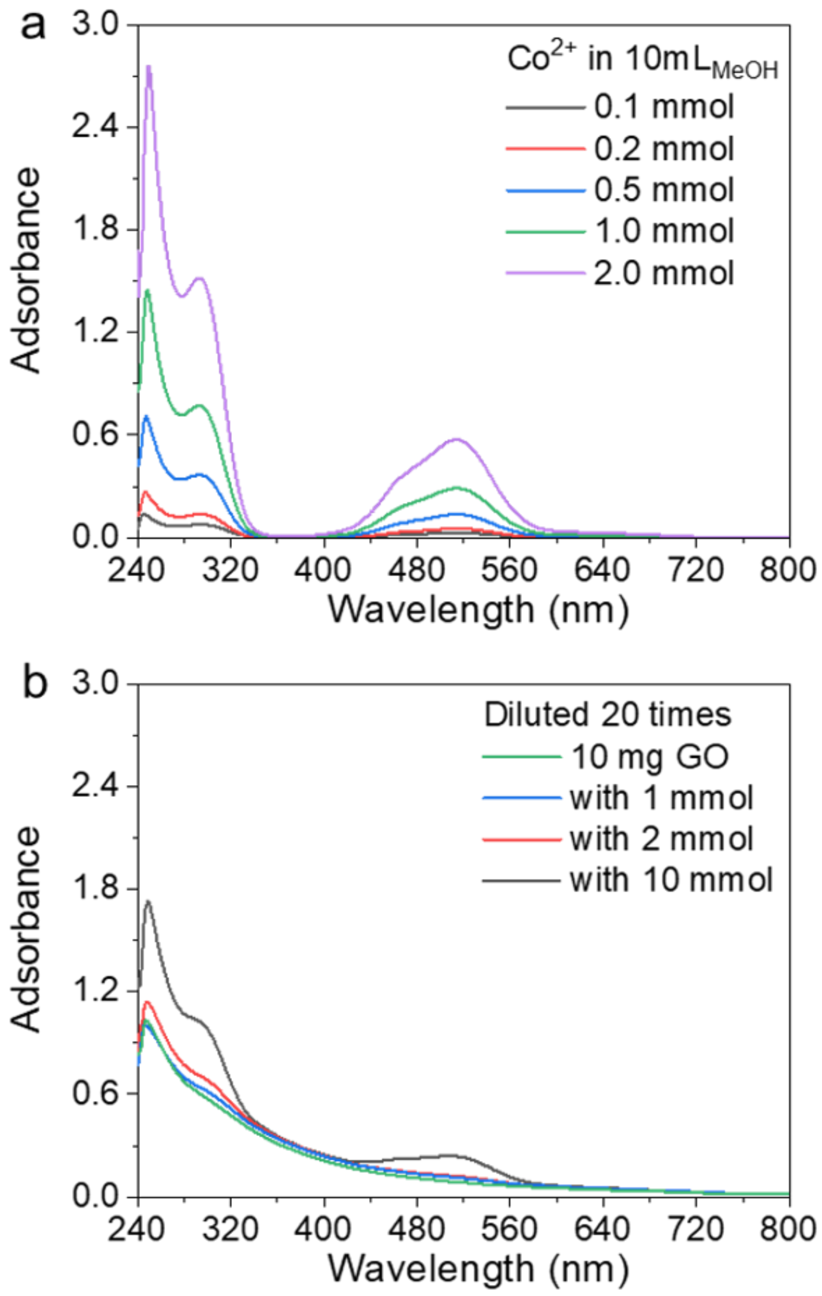


**Figure S1. Ultraviolet-visible (UV-Vis) spectroscopy analysis of Co(II) adsorption amounts.** Those for a) diluted Co(II) nitrate in 10 mL of methanol as a function of concentration and b) 10 mg graphene oxide (GO) dispersed in cobalt solution as a function of concentration after being diluted 20 times. The inset graph depicts Co(II) functional peaks around 550 nm.

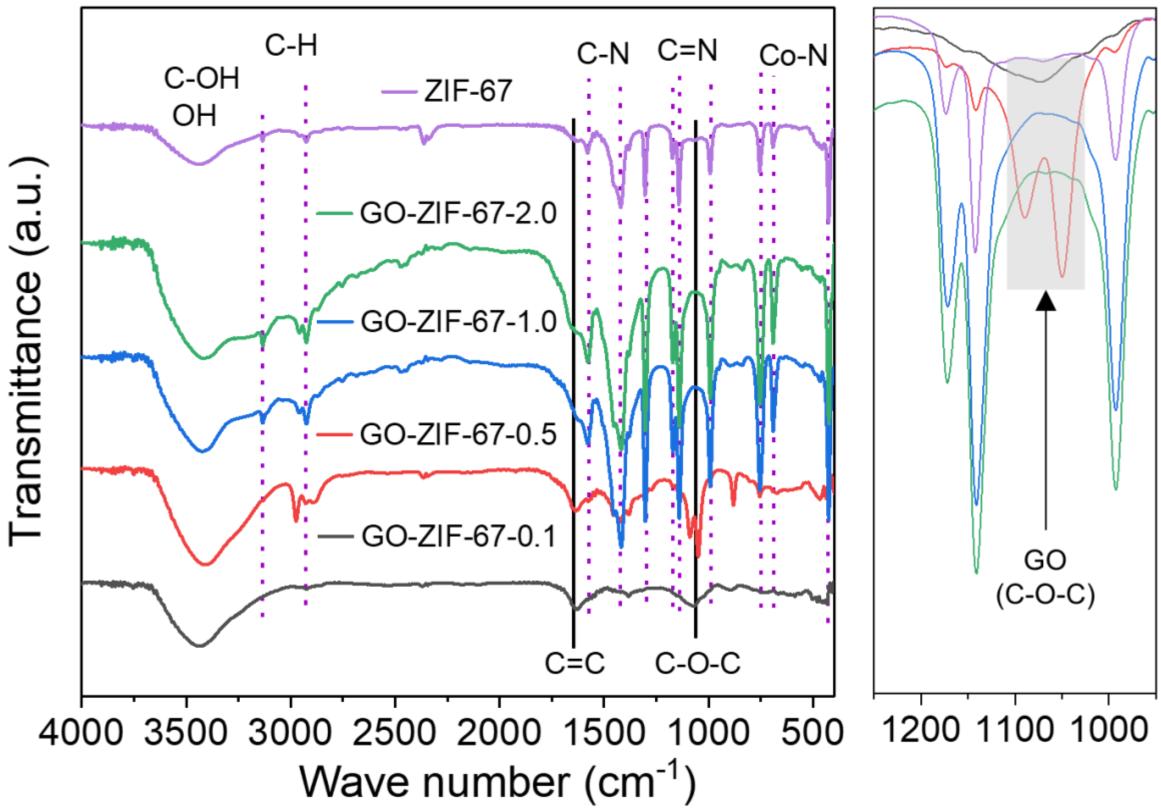


**Figure S2. Fourier-transform infrared (FT-IR) spectroscopy analysis of GO-ZIF-67-x as a function of used Co(II) moles.**

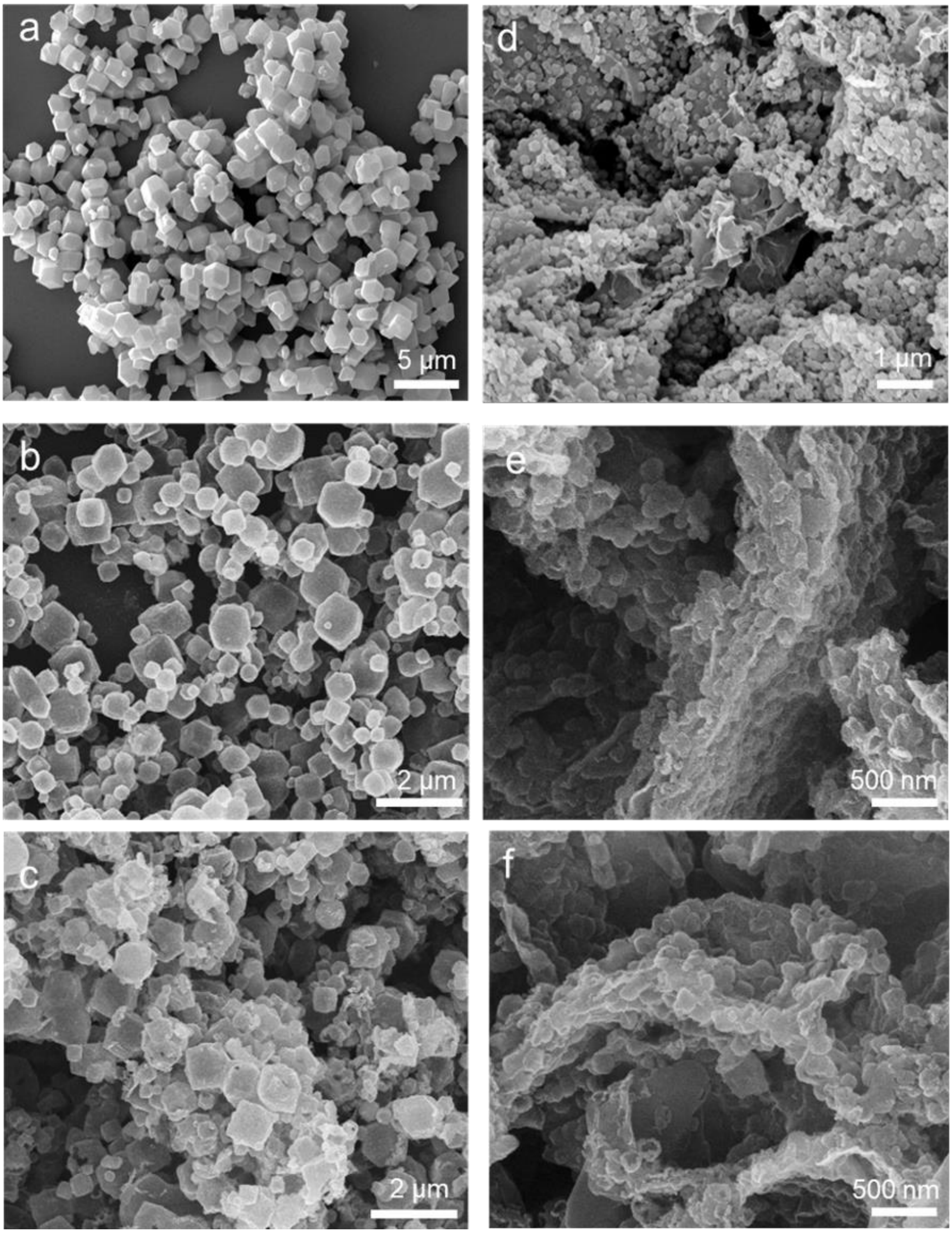


**Figure S3. Scanning electron microscopy (SEM) analysis.** a-c) SEM images of ZIF-67, $Co_3S_4$, and $Co_3S_4/MoS_2$. d-f) SEM images of GO-ZIF-67, G-$Co_3S_4$, and G-SHELL.

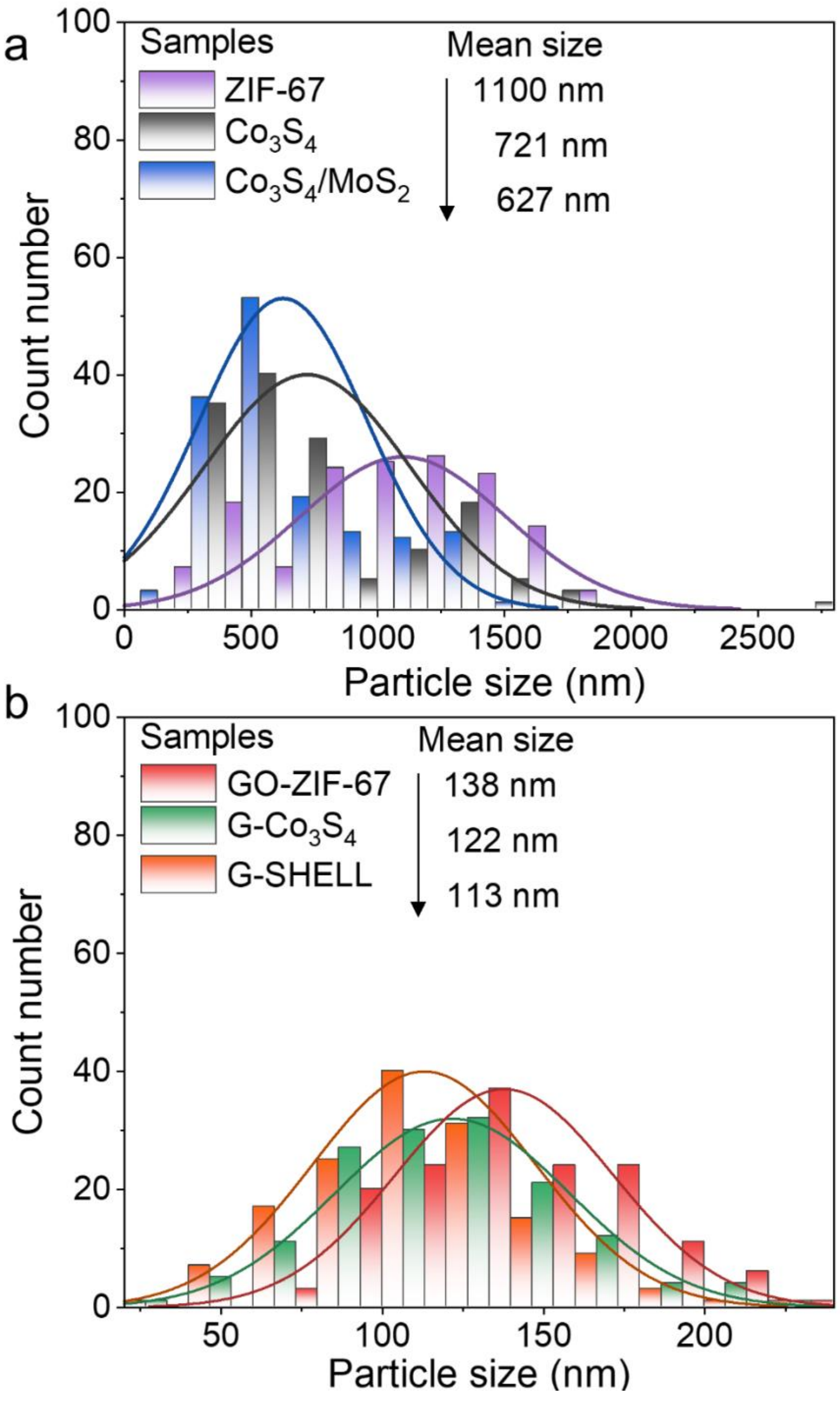


**Figure S4. Particle size distributions of electrocatalysts.** a) ZIF-67 with its derived catalyst and b) GO-ZIF-67 with its derived catalyst, which was derived by counting 150 samples for each SEM image in Figure S3.

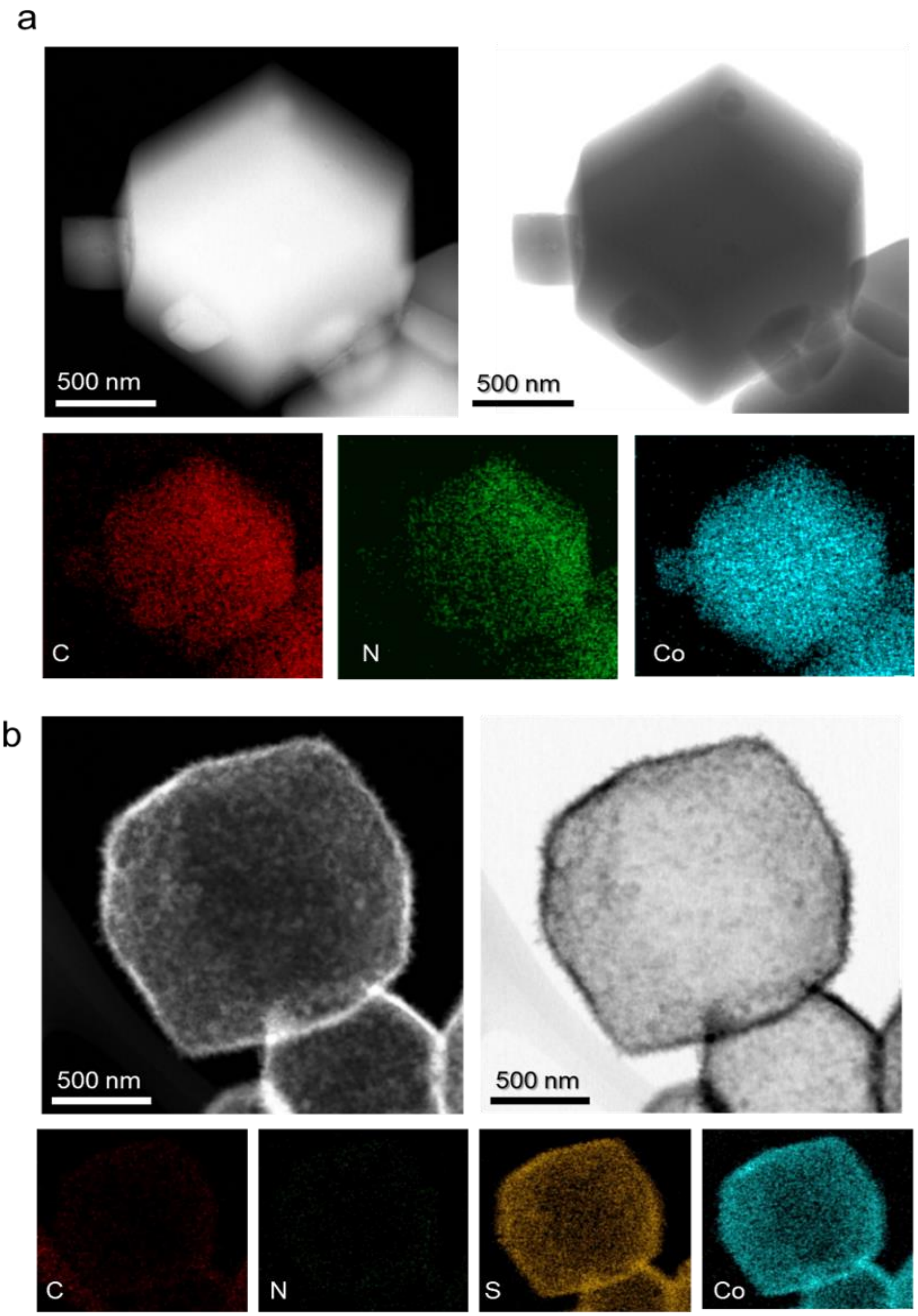


**Figure S5. Scanning electron microscopy-energy dispersive spectroscopy (STEM-EDS) analysis.** STEM-EDS images of a) ZIF-67 and b) $Co_3S_4$.

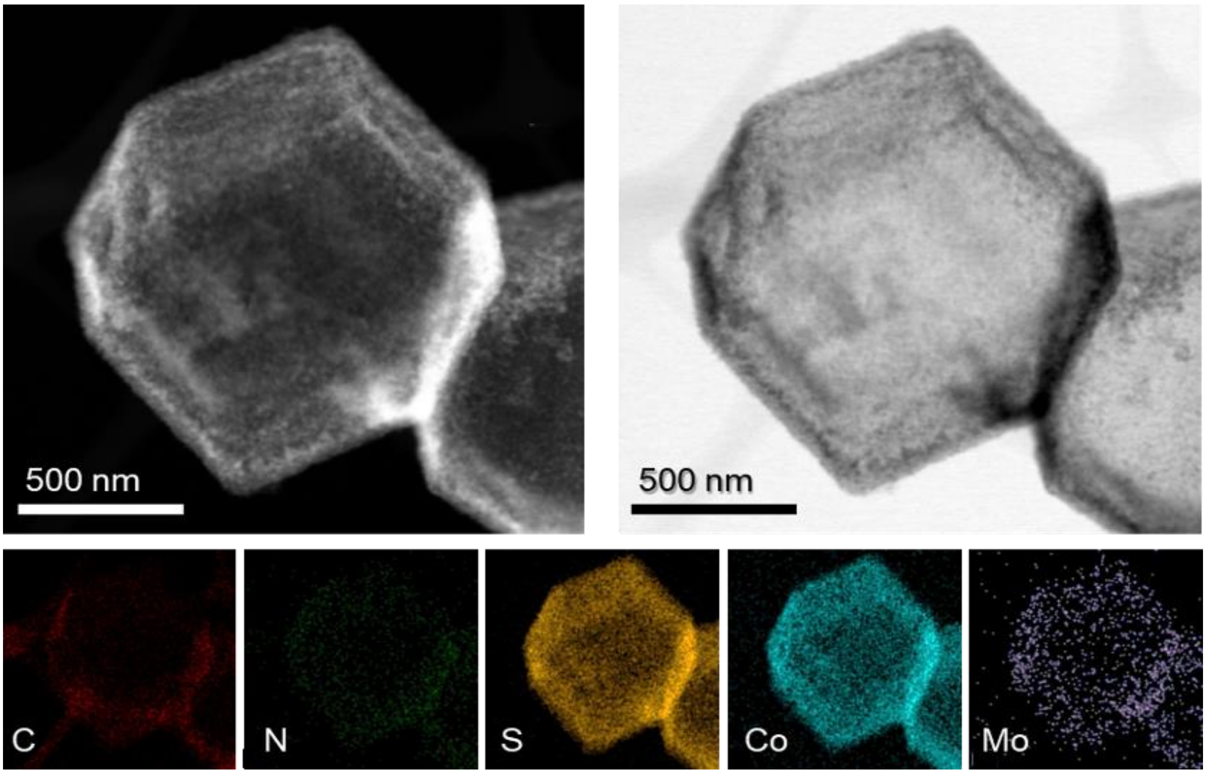


**Figure S6. STEM-EDS images of $Co_3S_4/MoS_2$.**

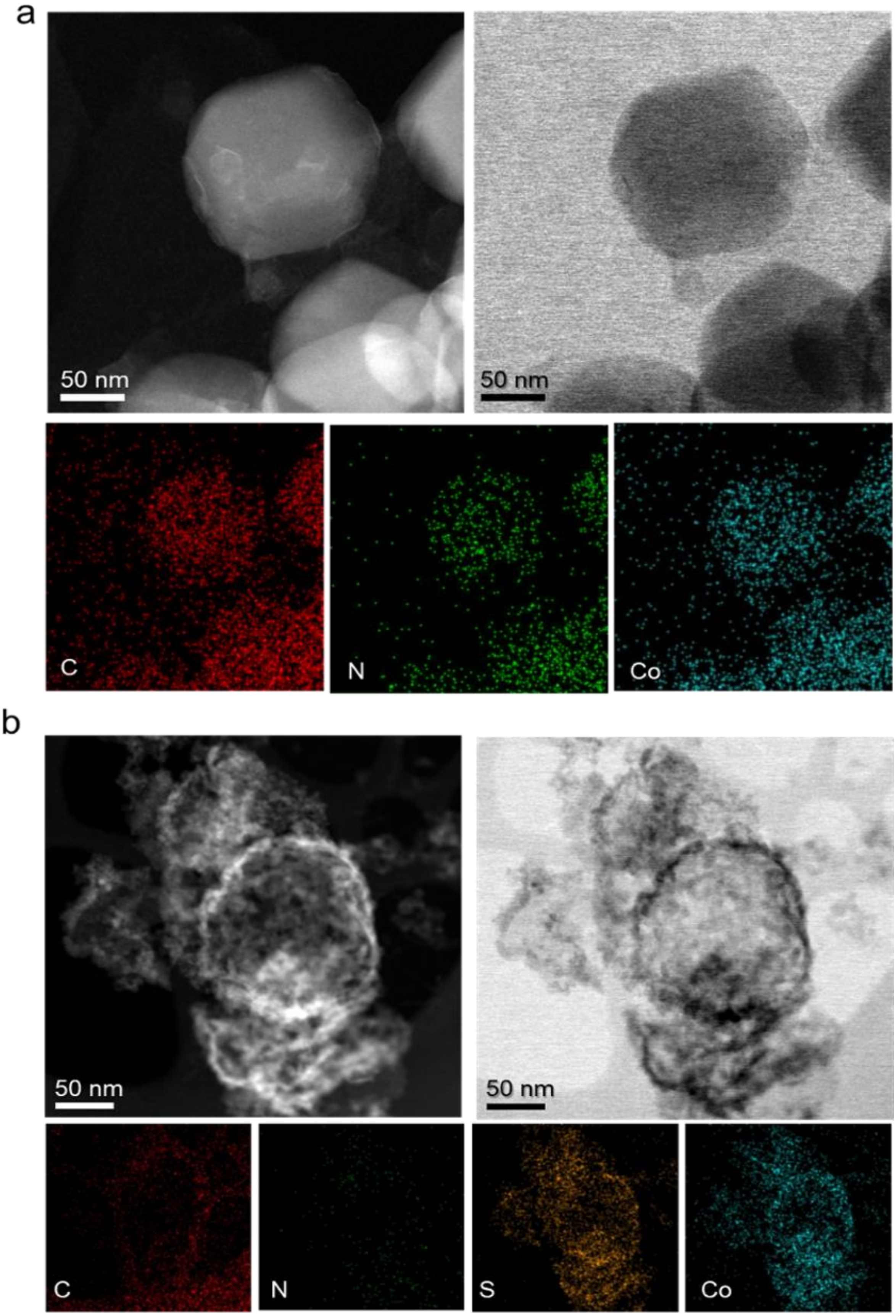


**Figure S7. STEM-EDS analysis.** STEM-EDS images of a) GO-ZIF-67 and b) G-$Co_3S_4$.

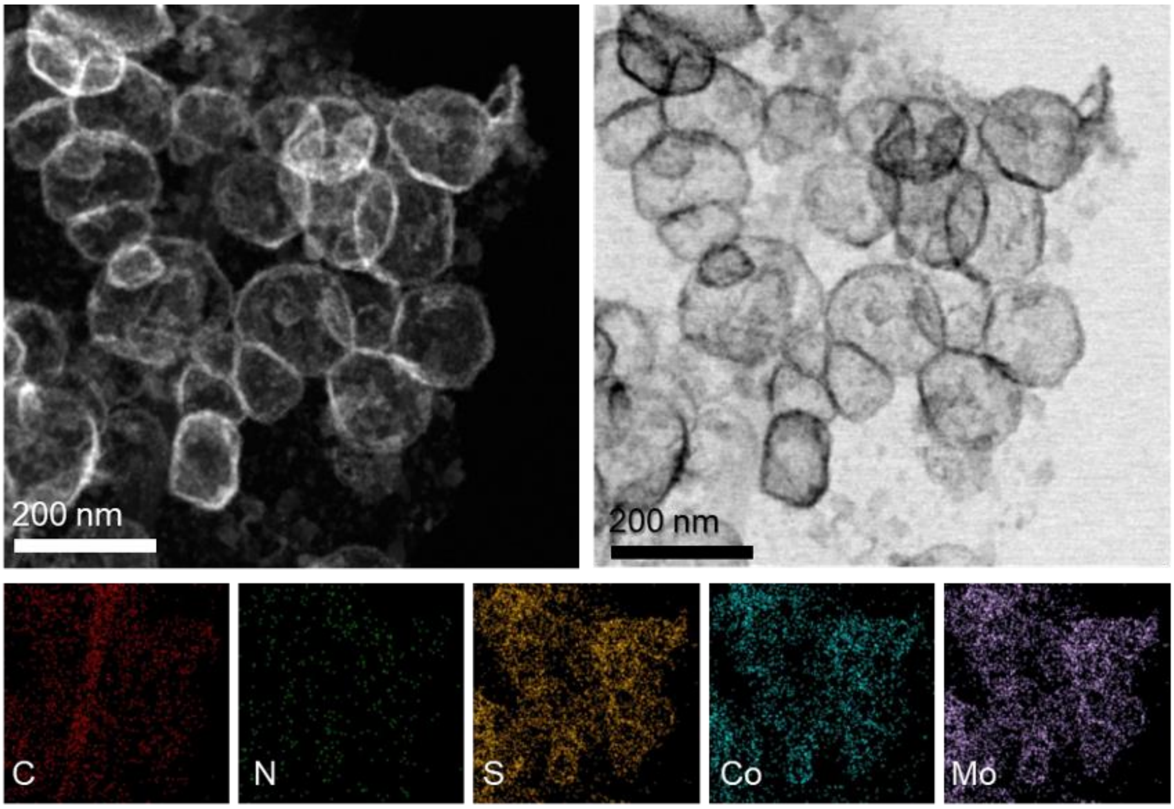


**Figure S8. STEM-EDS analysis of G-SHELL.**

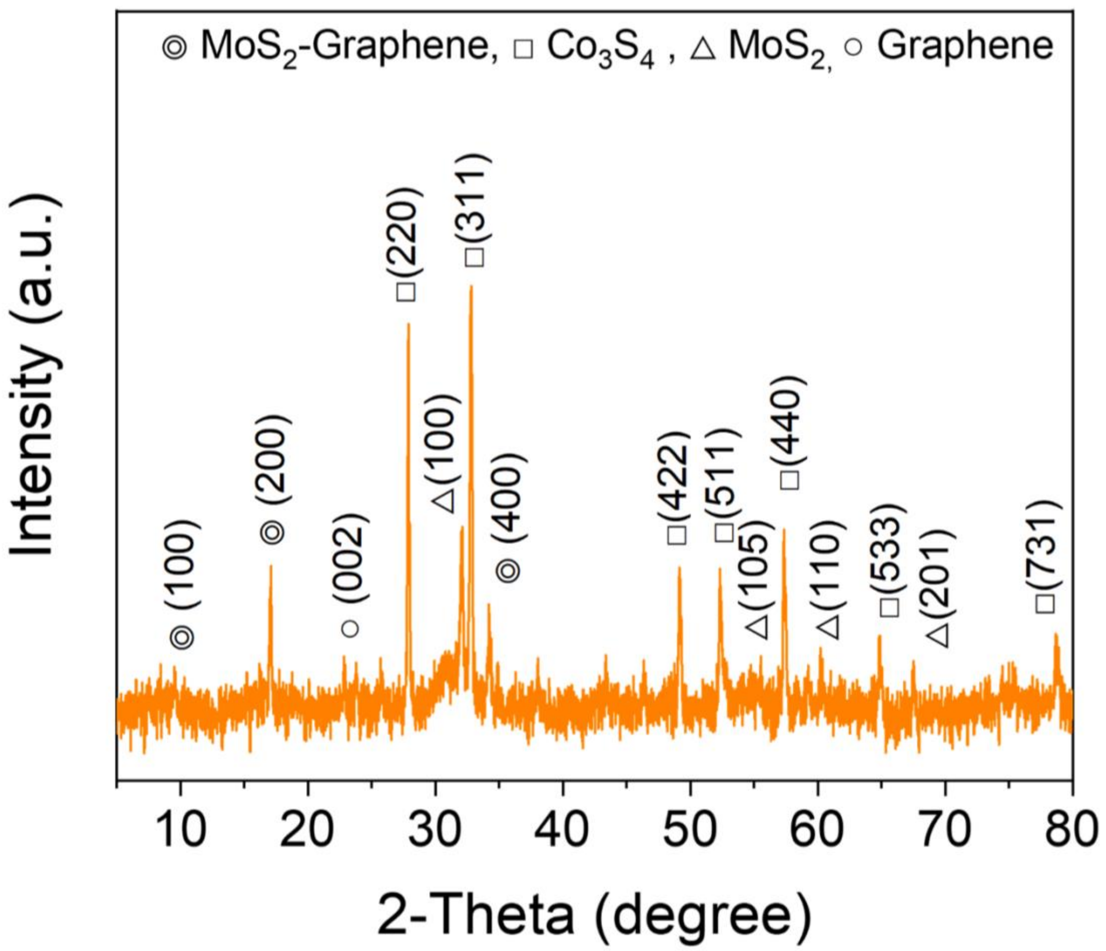


**Figure S9. XRD analysis of G-SHELL.**

(unit: e Å$^{-3}$).

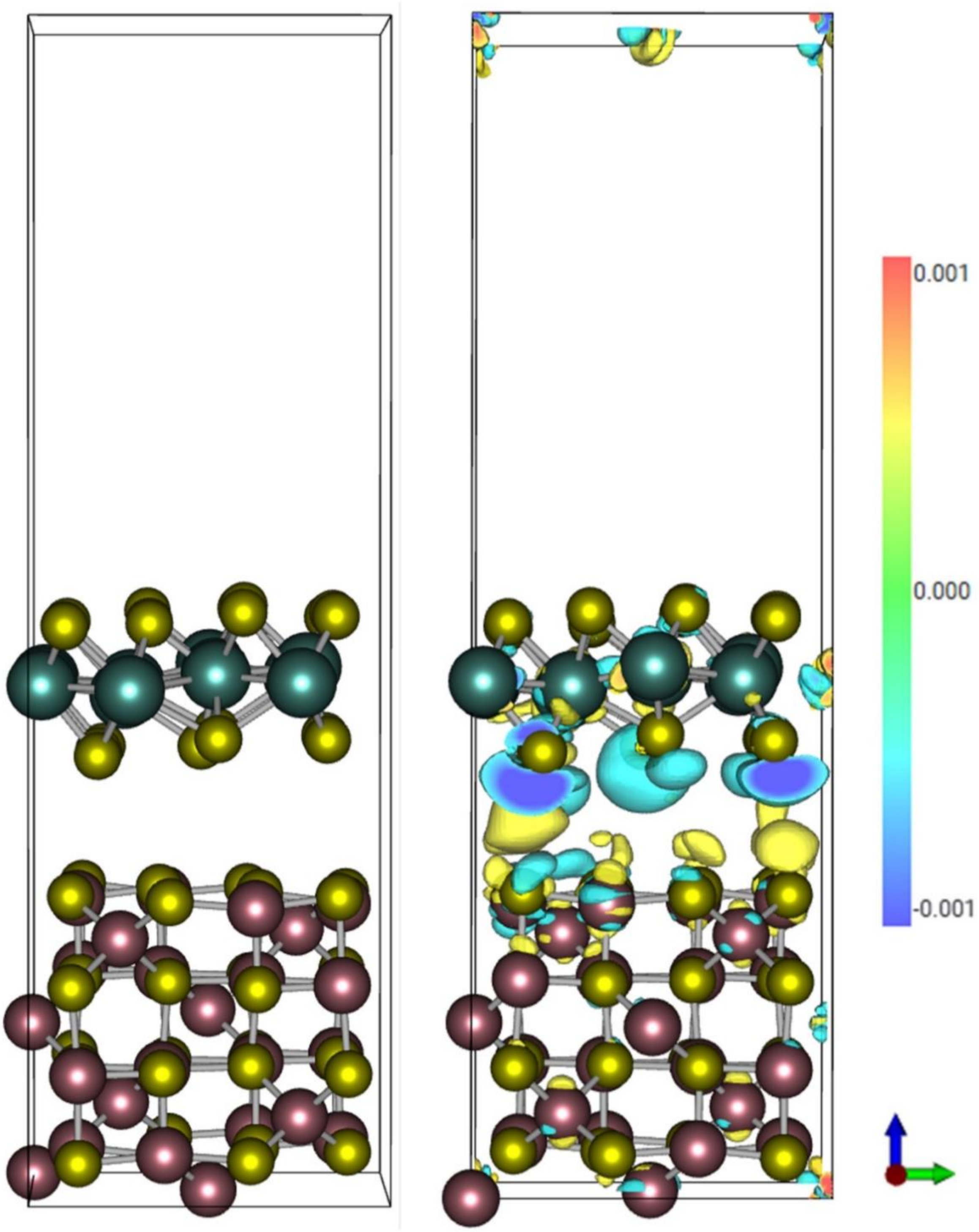


**Figure S10. DFT calculation of $Co_3S_4/MoS_2$ structure and charge density difference plot (unit: e Å$^{-3}$).**

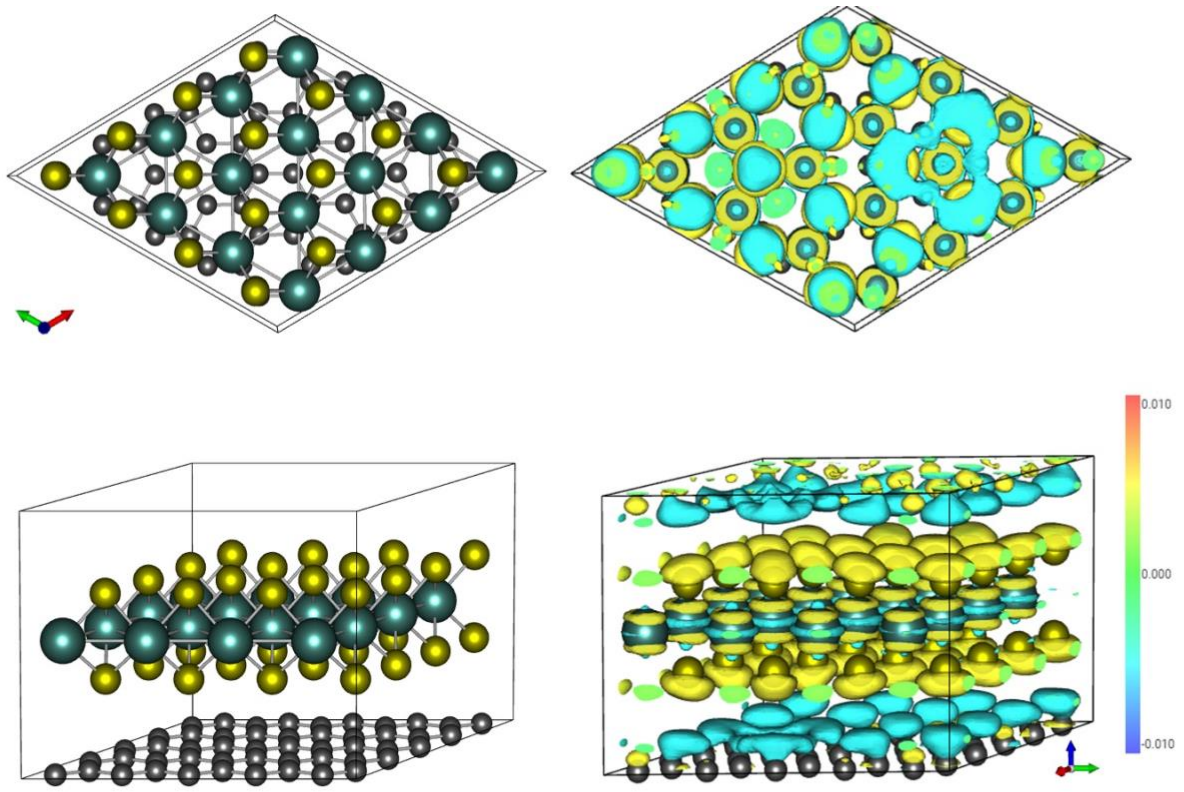


**Figure S11. DFT calculation of $MoS_2$/Graphene structure and charge density difference plot (unit: e Å$^{-3}$).**

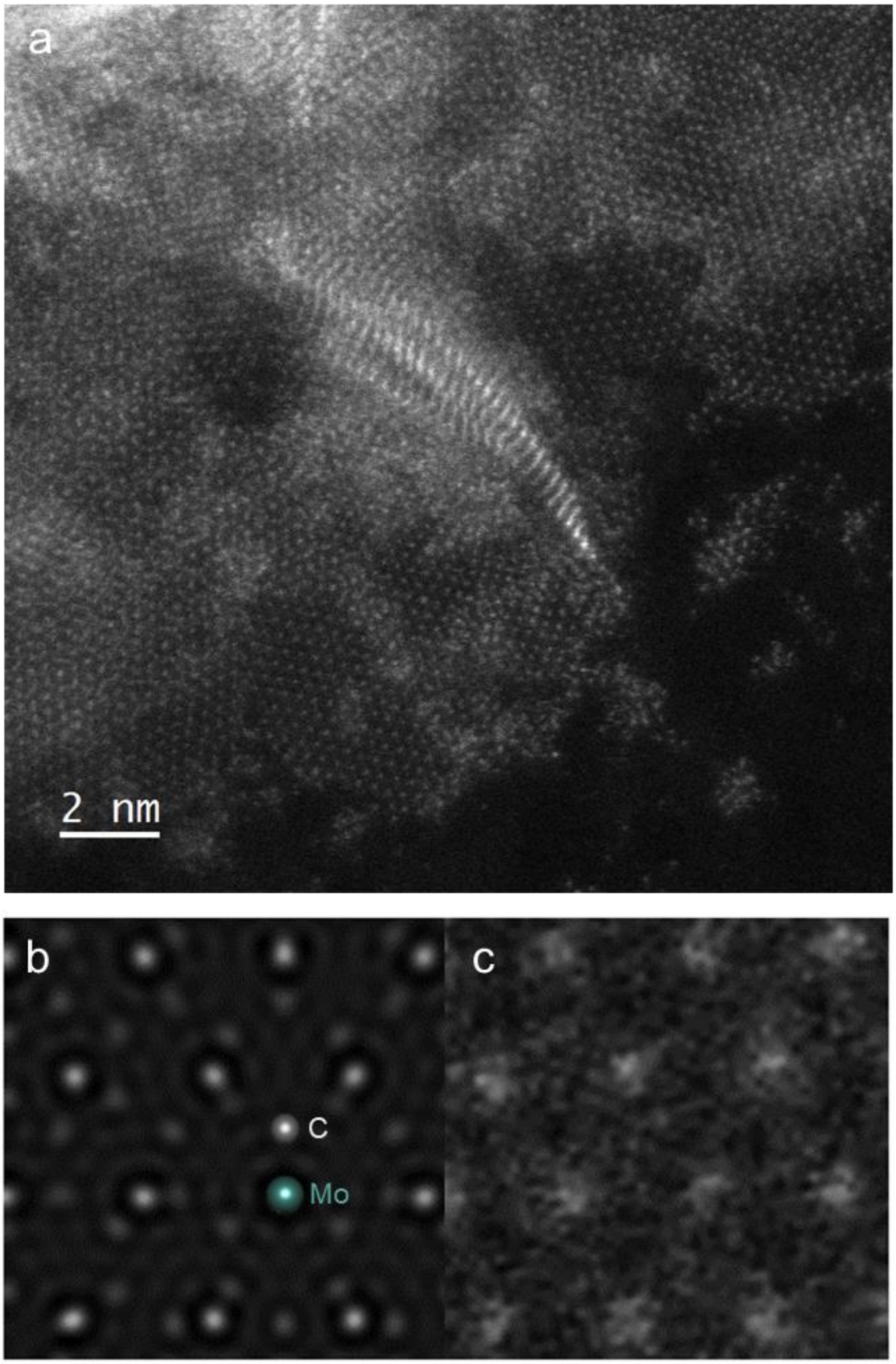


**Figure S12. Atomic-resolution STEM images of G-SHELL.** a) STEM image of G-SHELL. b) Simulated STEM image of $MoS_2$/Graphene heterostructure, and c) atomic STEM image of G-SHELL. The length and width of images b) and c) are 1 nm X 1 nm.

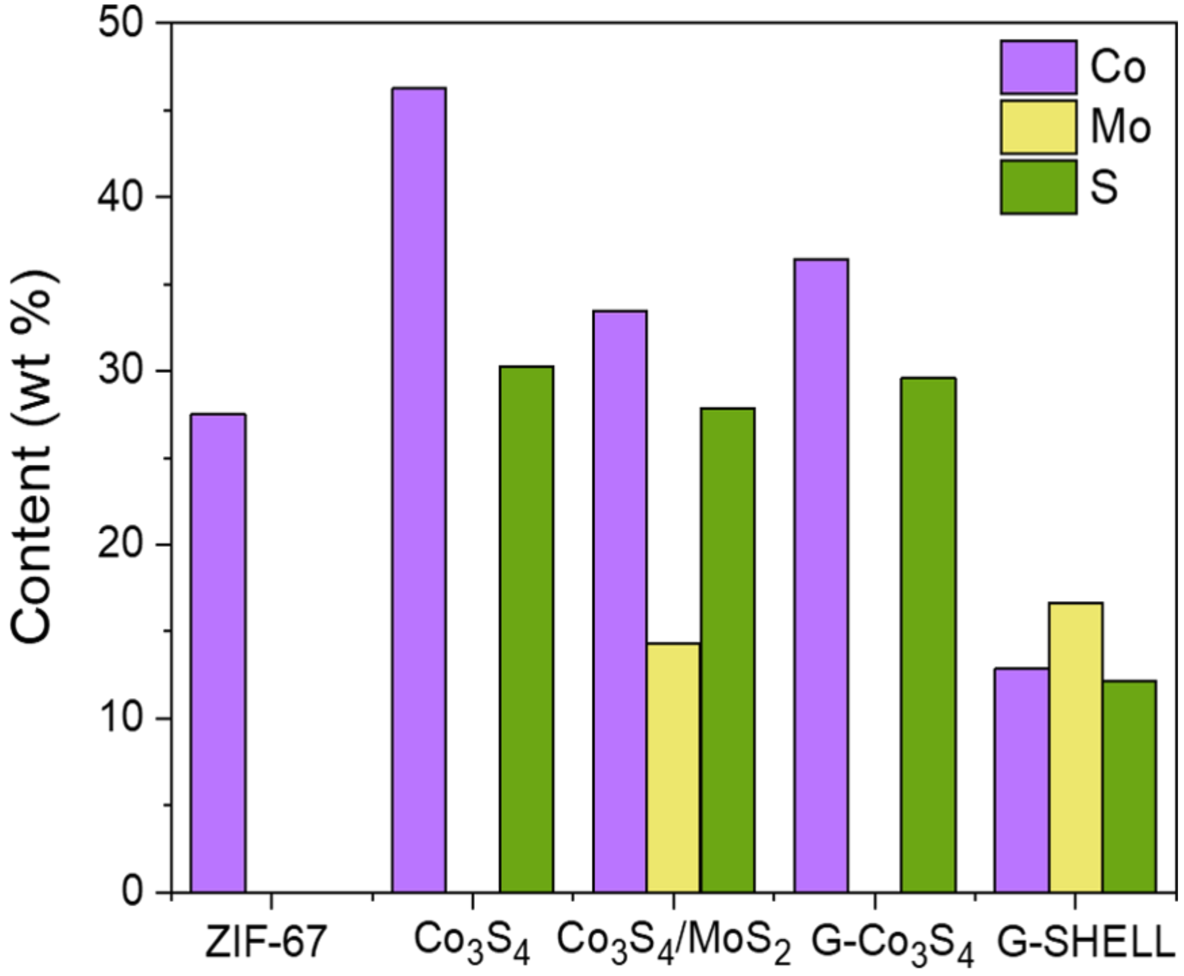


**Figure S13. ICP-MS of ZIF-67, $Co_3S_4$, $Co_3S_4/MoS_2$, G-$Co_3S_4$, and G-SHELL.**

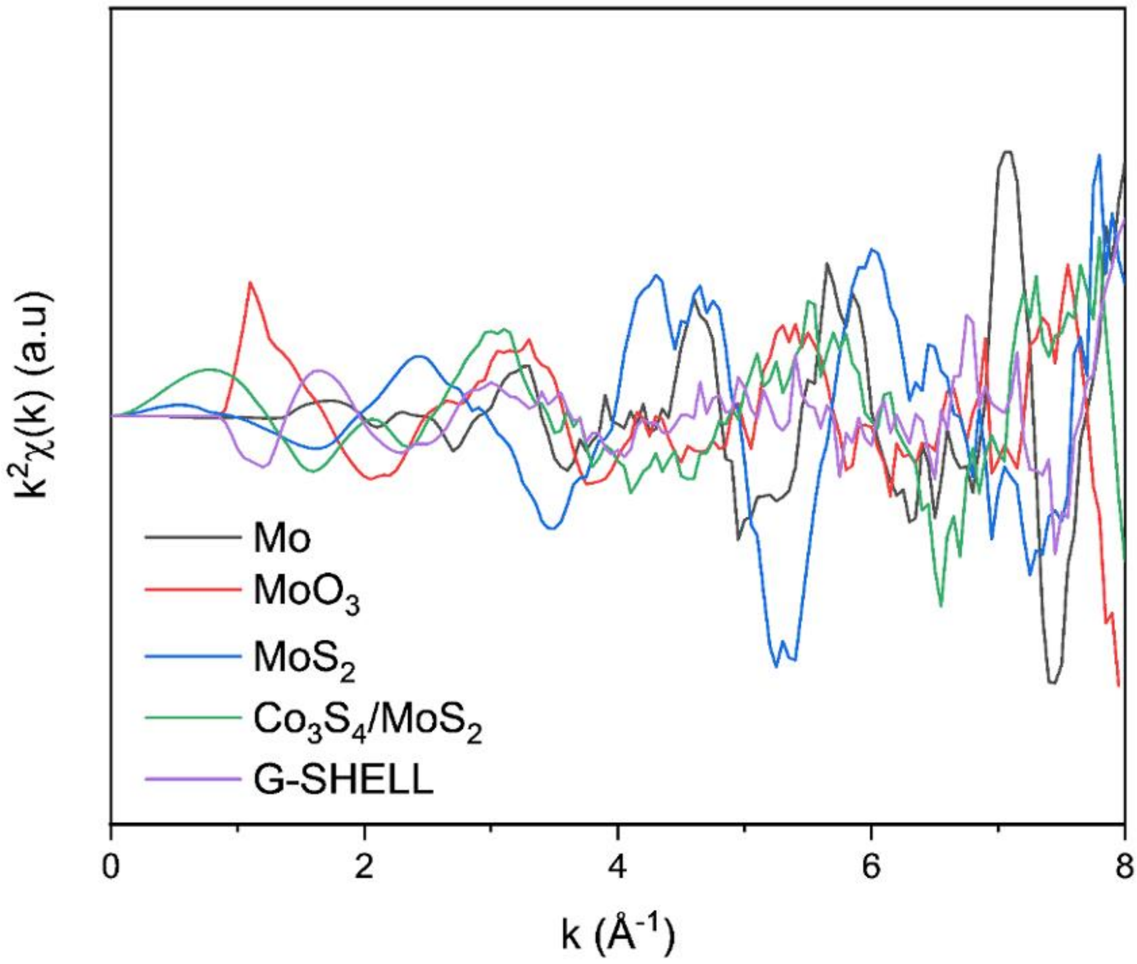


**Figure S14.** **$k^2$-weighted Fourier transformation of Mo K edge spectra.**

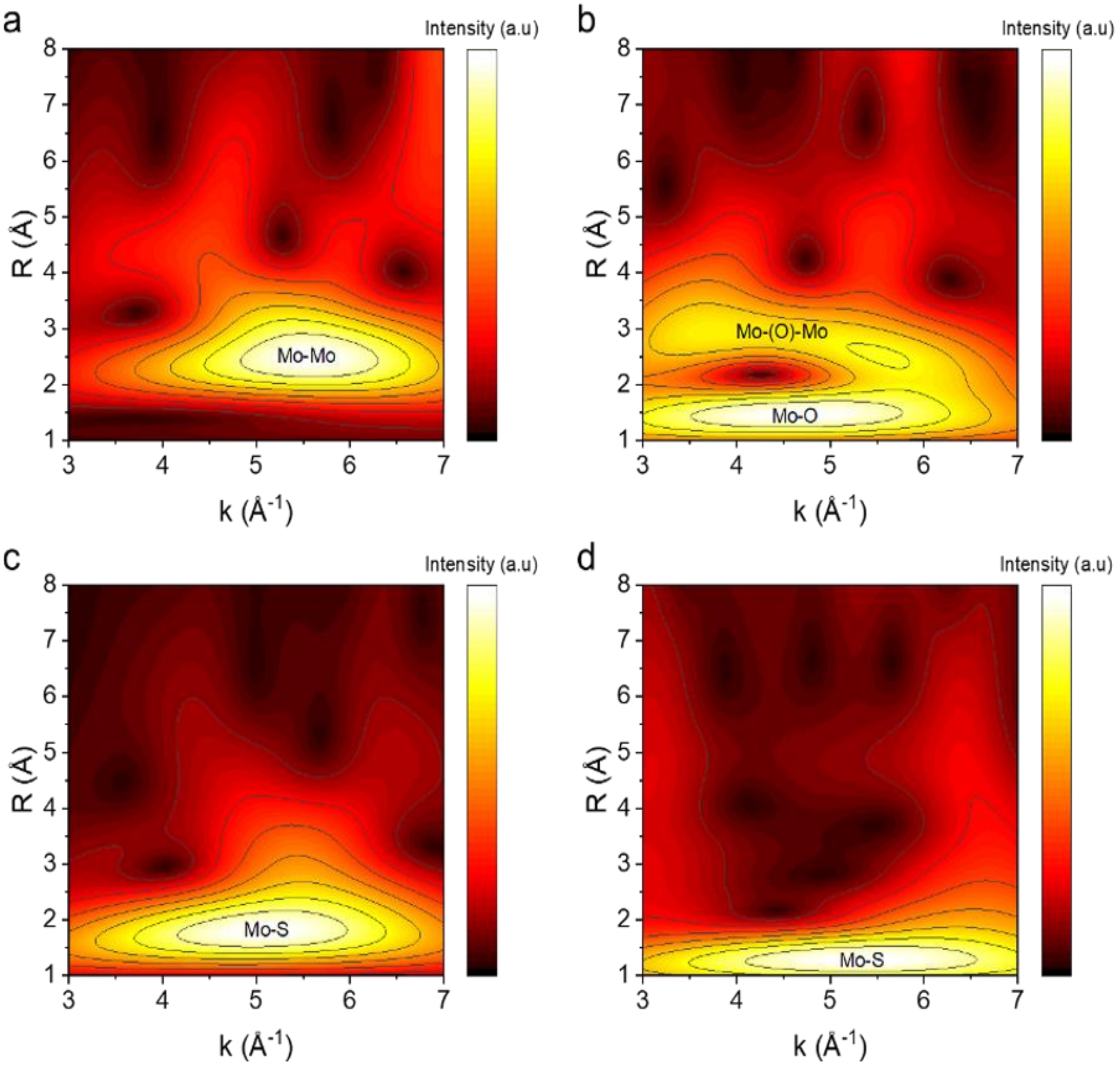


**Figure S15. Wavelet transformation of Mo K edge spectra.** a) Mo, b) $MoO_3$, c) $MoS_2$, and d) $Co_3S_4$/ $MoS_2$.

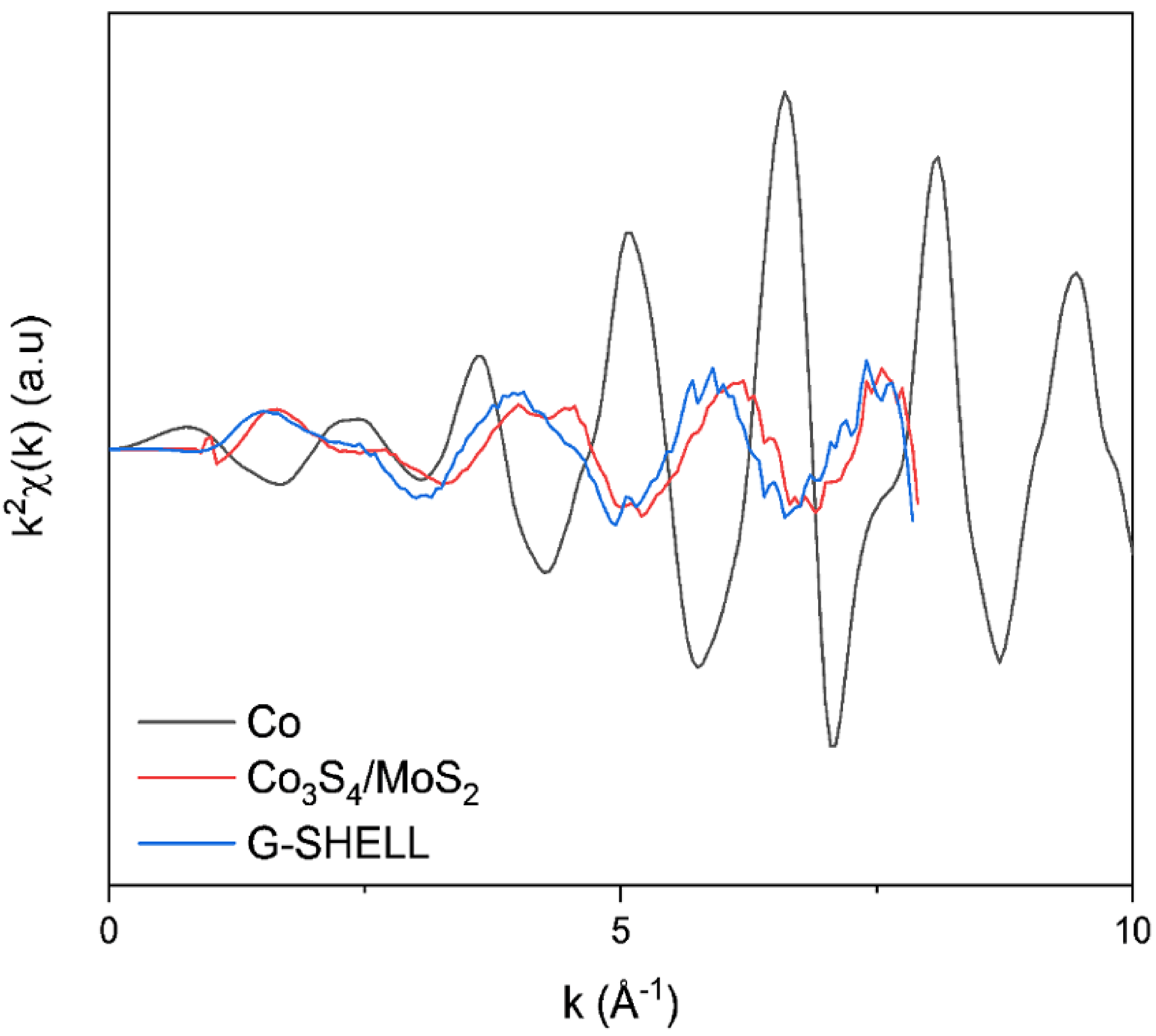


**Figure S16.** **$k^2$-weighted Fourier transformation of Co K edge spectra.**

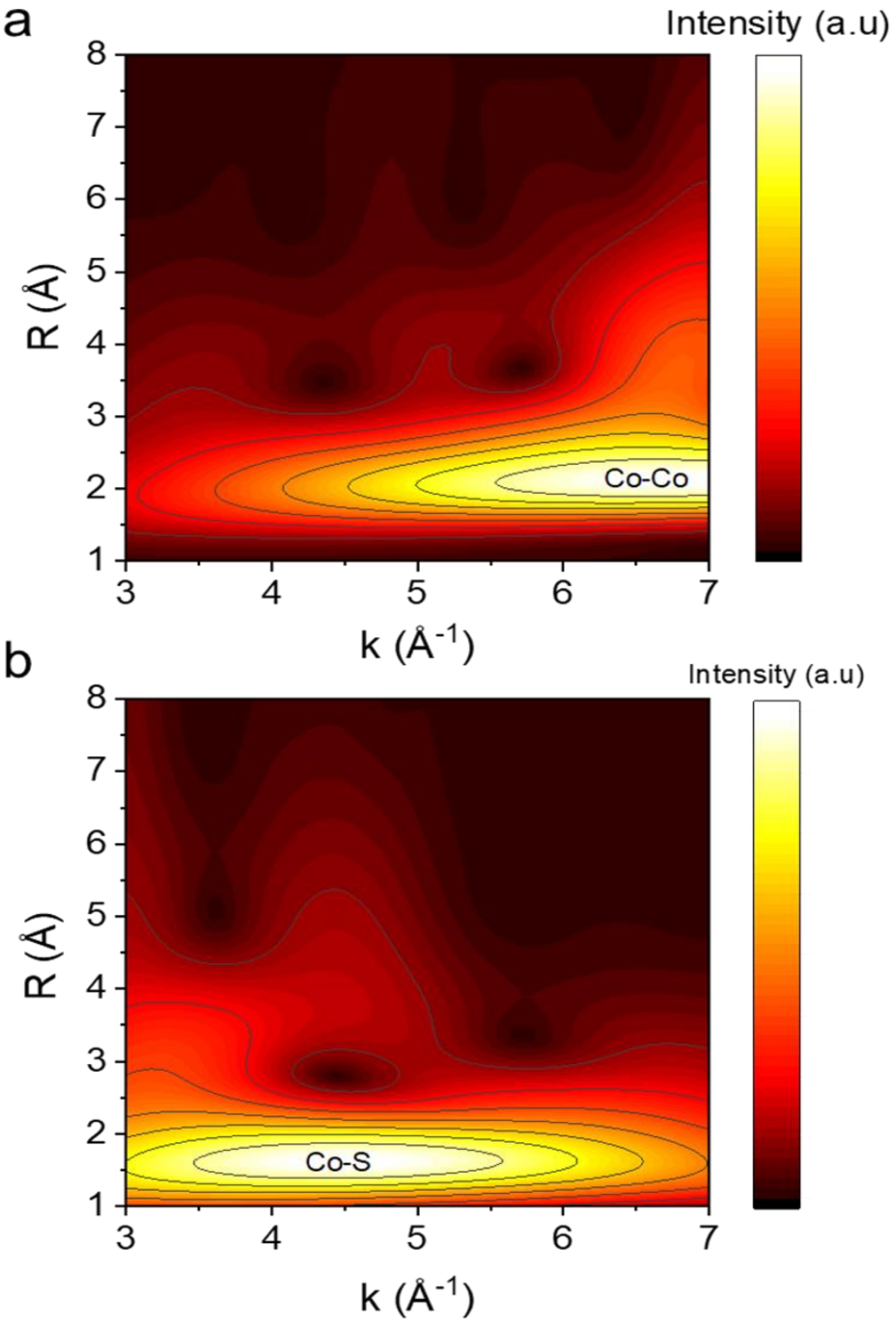


**Figure S17. Wavelet transformation of Co K edge spectra.** a) Co and b) $Co_3S_4$.

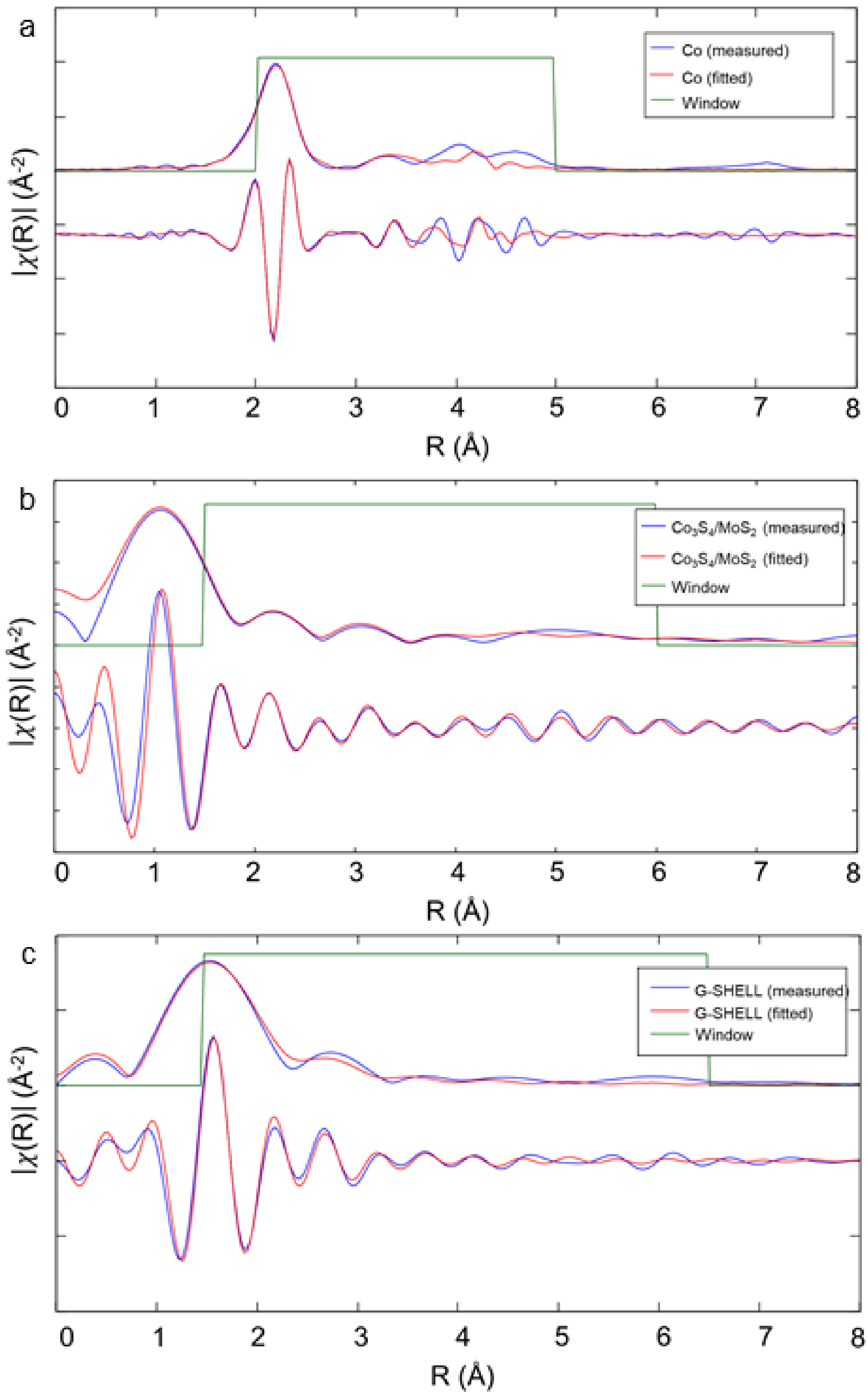


**Figure S18. Co K edge EXAFS spectra.** a) Co, b) $Co_3S_4/MoS_2$, and c) G-SHELL.

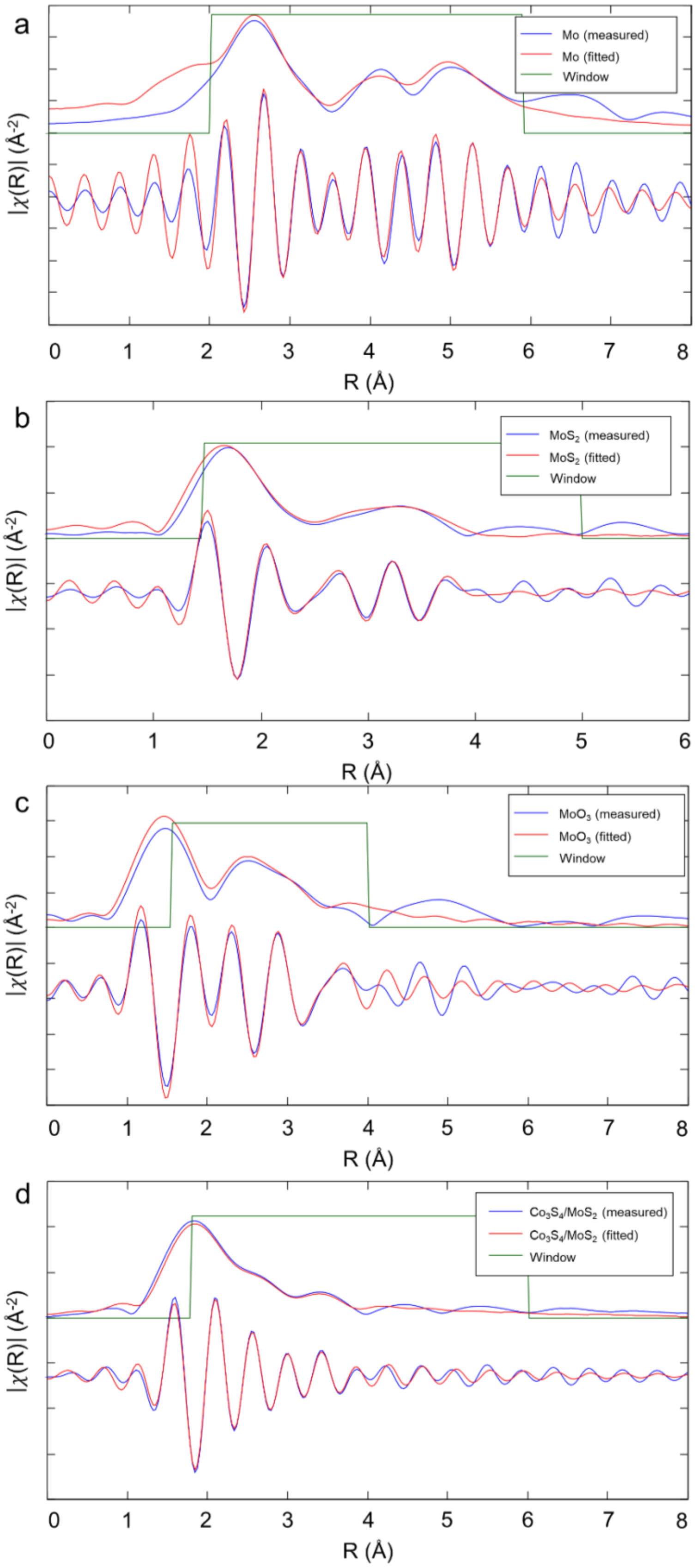


**Figure S19. Mo K edge EXAFS spectra.** a) Mo, b) $MoS_2$, c) $MoO_3$, and d) $Co_3S_4/MoS_2$.

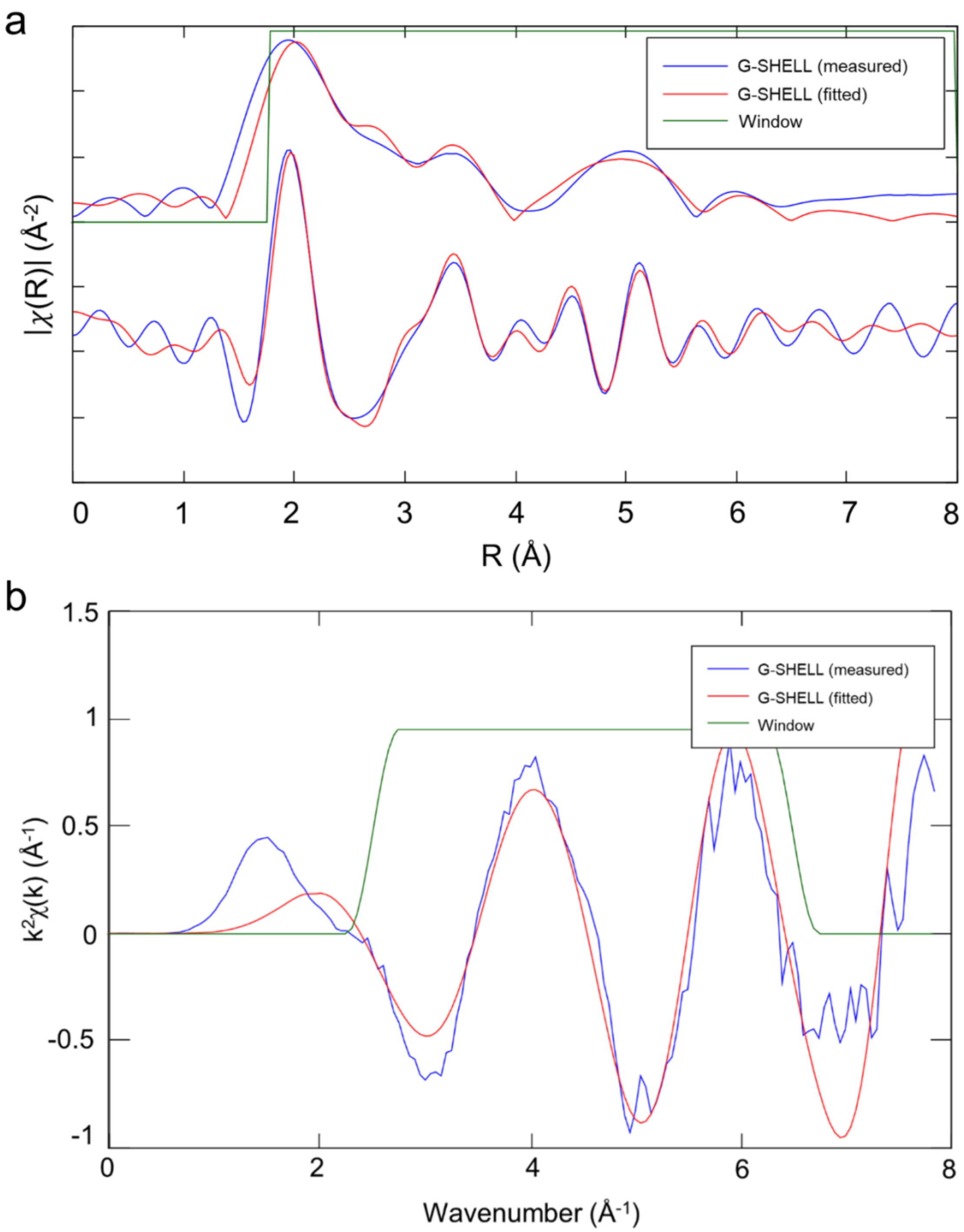


**Figure S20. Mo K edge EXAFS spectra of G-SHELL.** a) R-space and b) k-space.

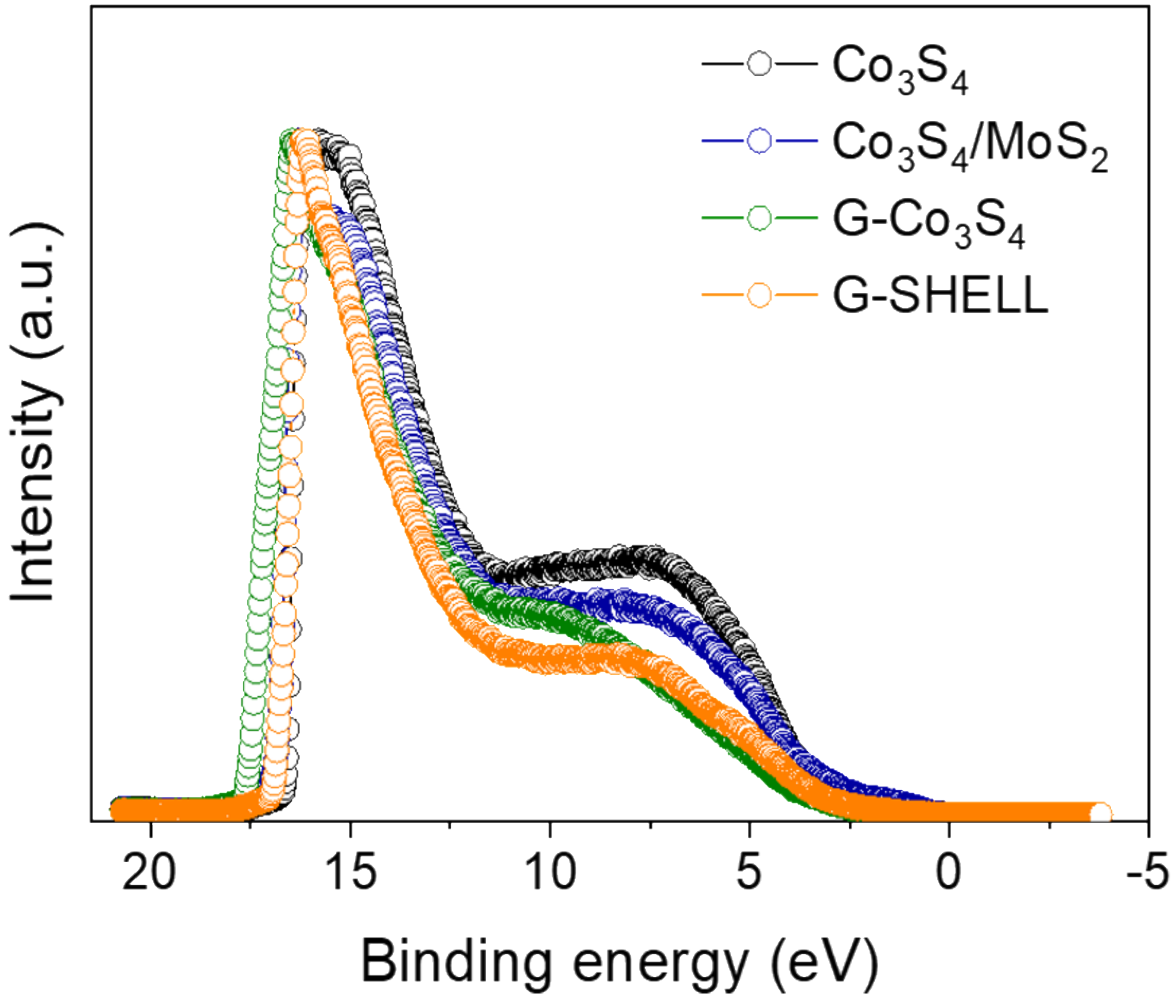


**Figure S21. Full-spectrum ultraviolet photoelectron spectroscopy (UPS) spectra of $Co_3S_4$, $Co_3S_4/MoS_2$, G-$Co_3S_4$, and G-SHELL.**

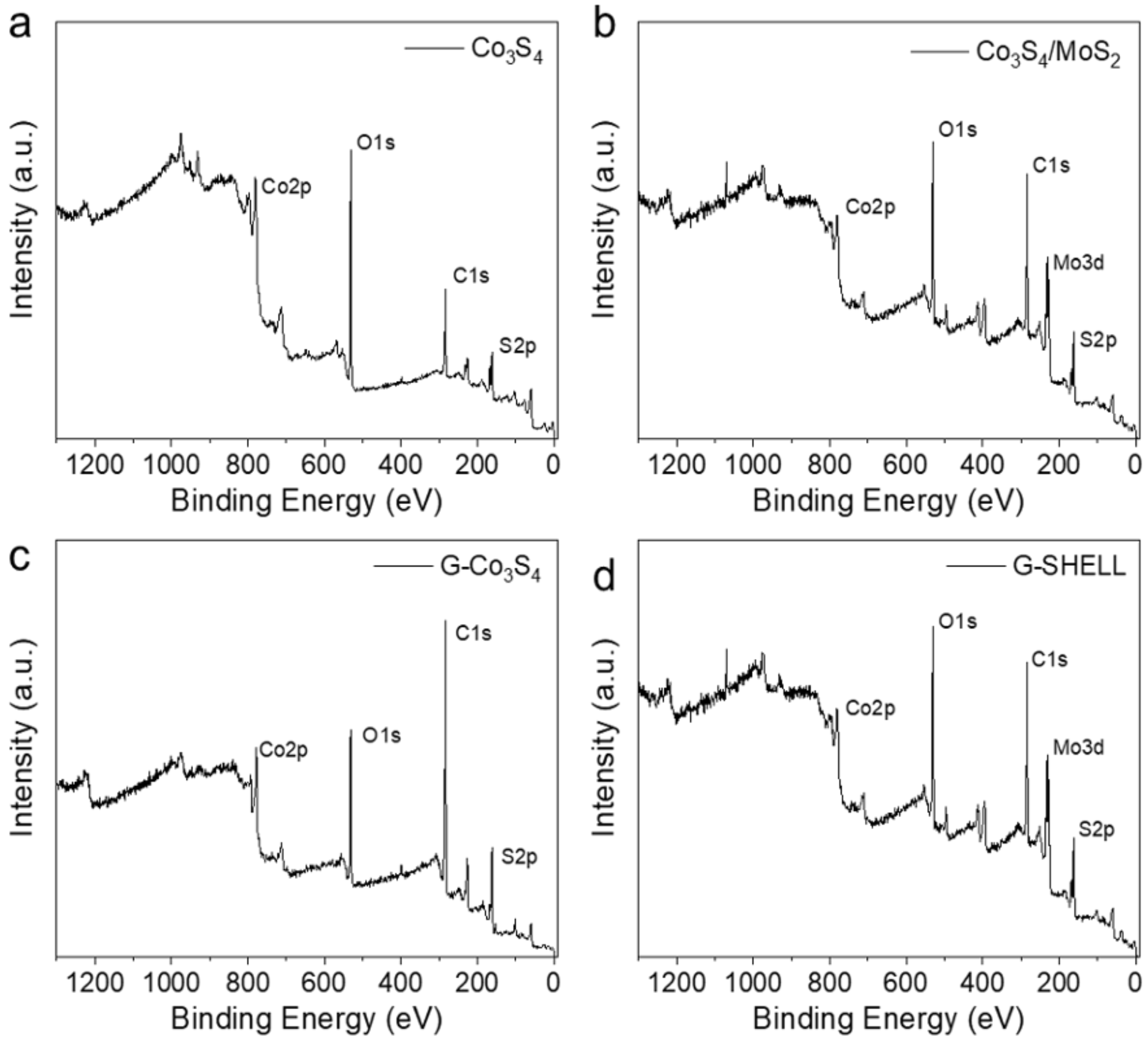


**Figure S22. X-ray photoelectron spectroscopy (XPS) survey spectra.** a) $Co_3S_4$, b) $Co_3S_4/MoS_2$, c) G-$Co_3S_4$, and d) G-SHELL.

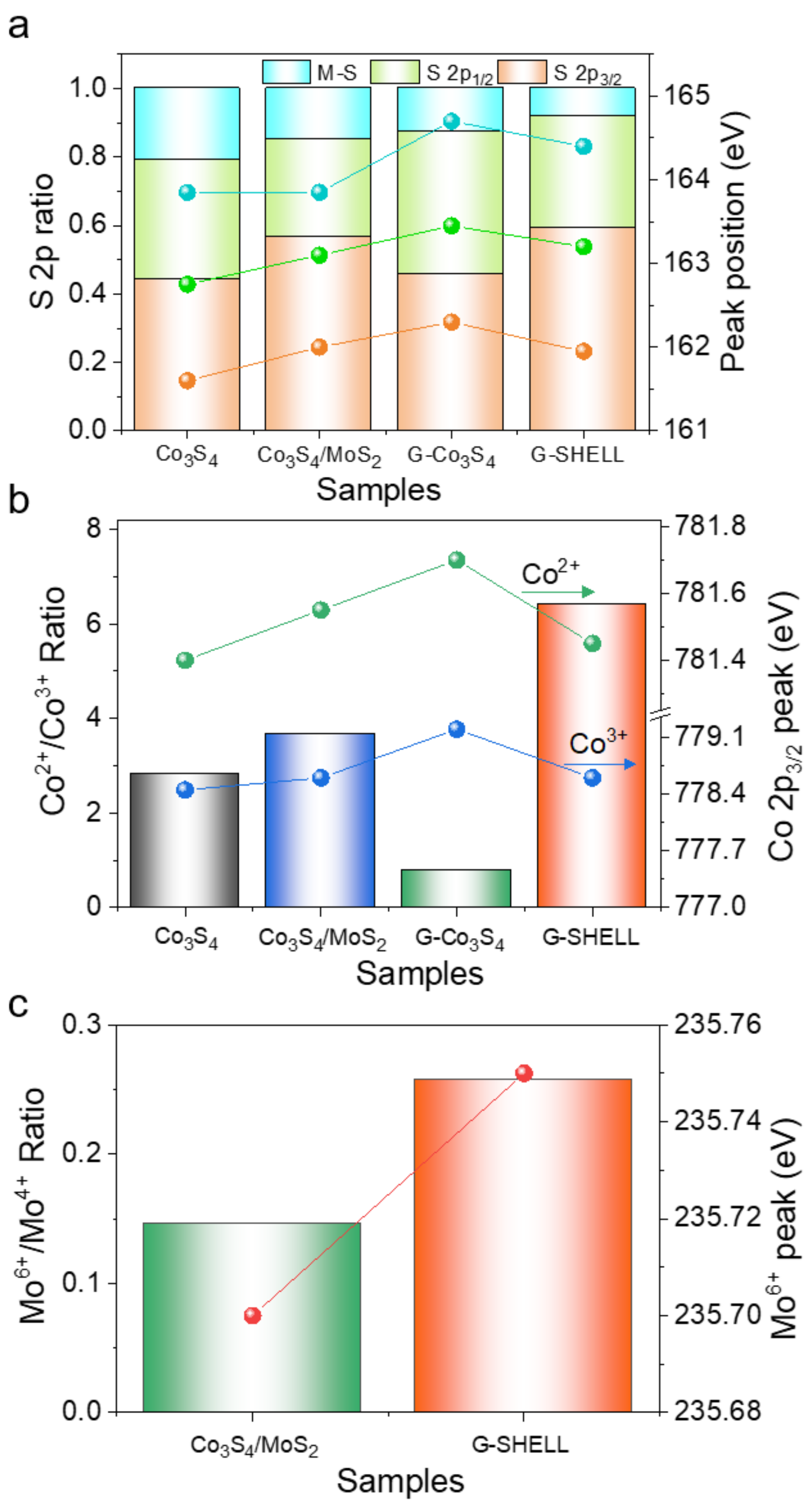


**Figure S23. XPS characteristics of different bonding species.** a) S 2p, b) Co 2p, and c) Mo 3d.

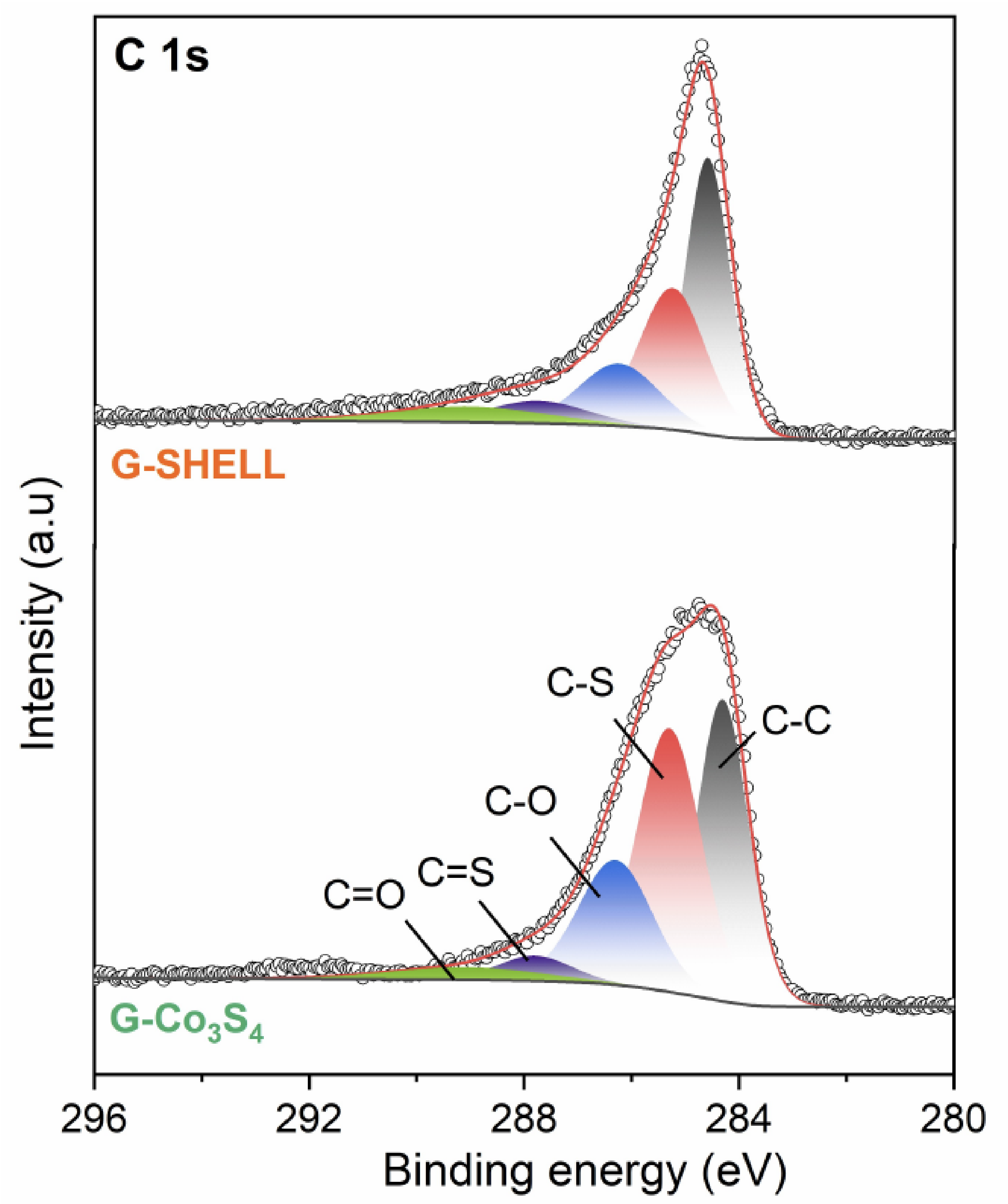


**Figure S24. XPS spectra for C1s orbitals of G-$Co_3S_4$ and G-SHELL.**

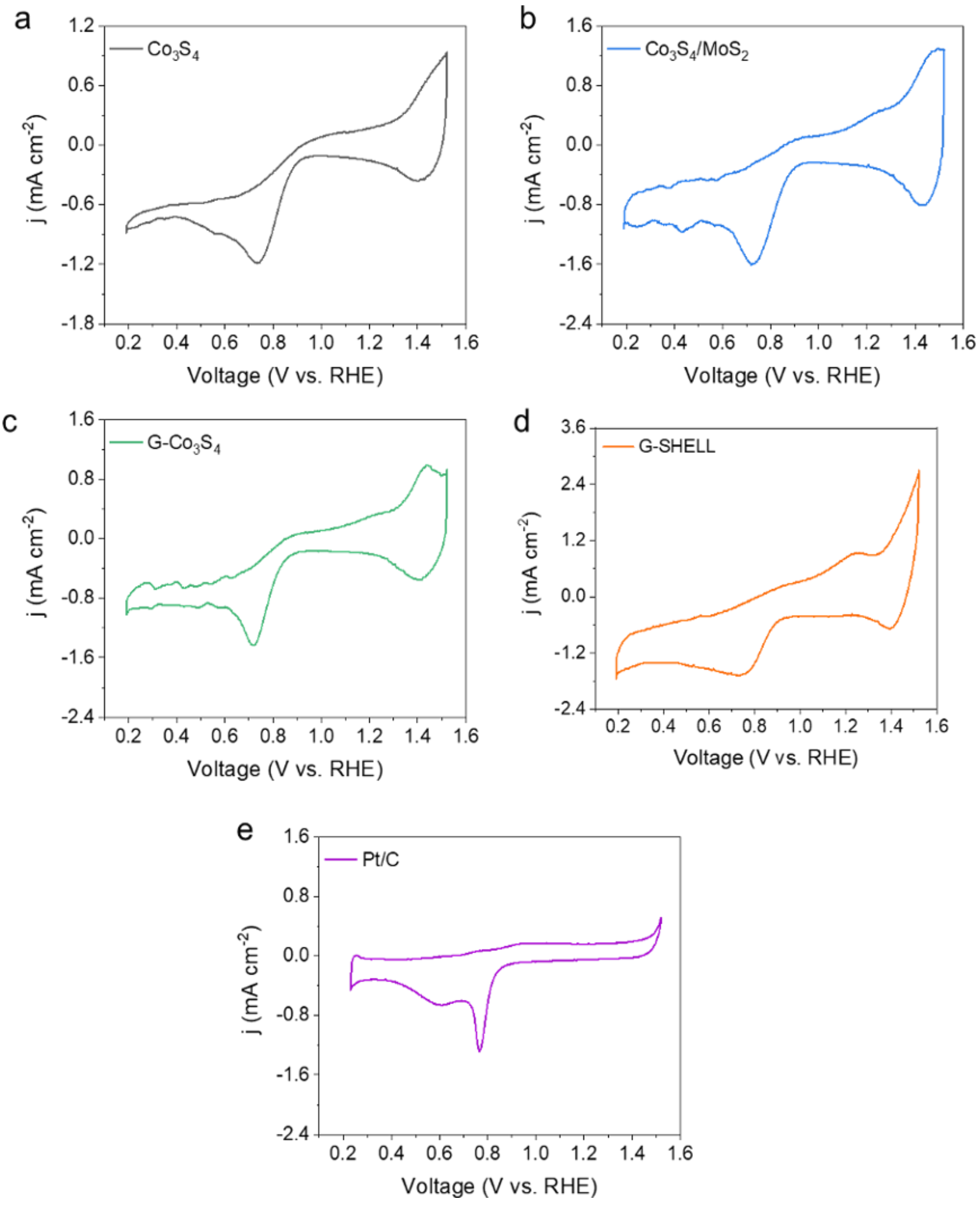


**Figure S25. Cyclic voltammetry (CV) curves of ORR in $O_2$-saturated 0.1 M KOH at 10mV $s^{-1}$.** a) $Co_3S_4$, b) $Co_3S_4/MoS_2$, c) G-$Co_3S_4$, d) G-SHELL, and e) Pt/C.

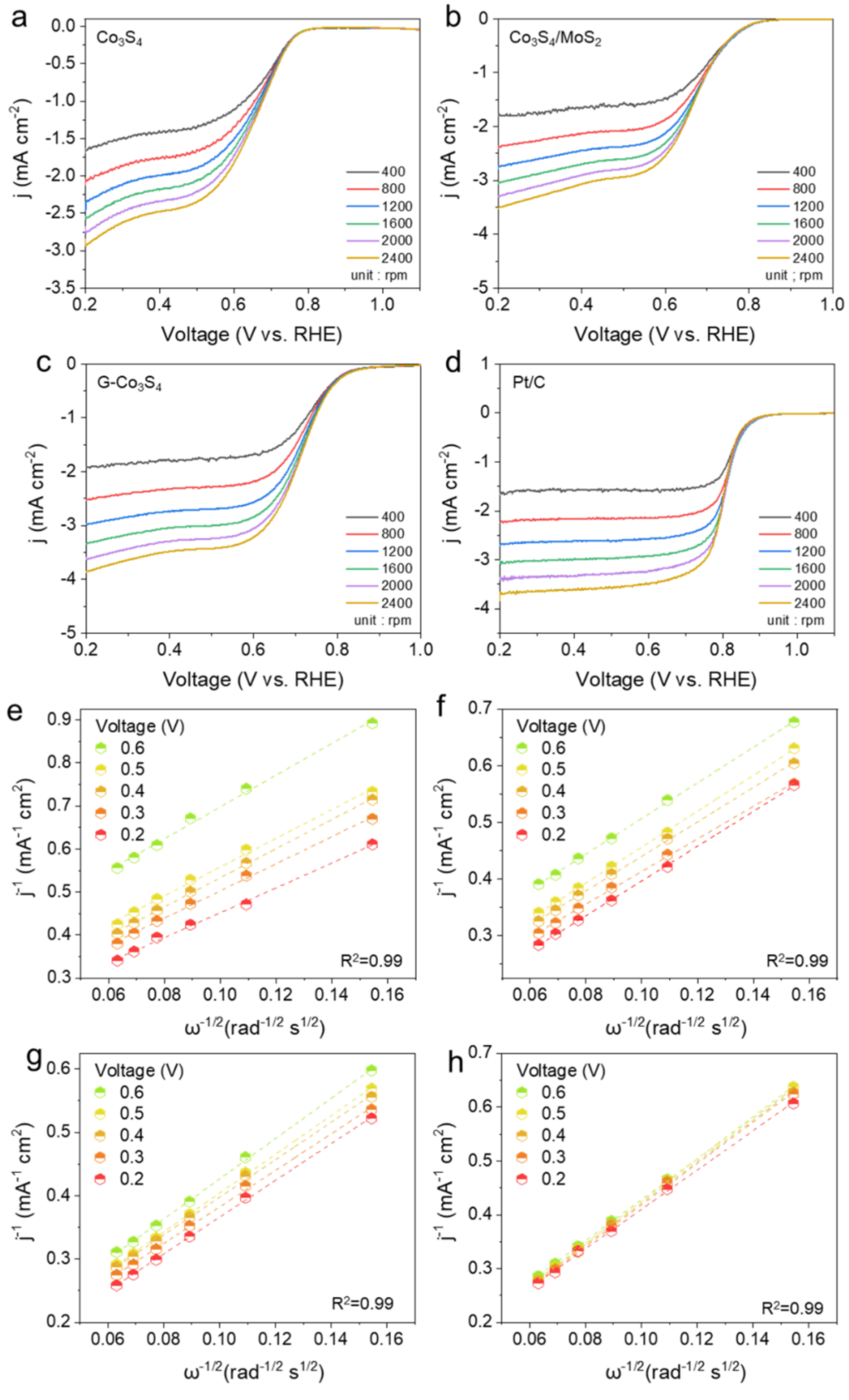


**Figure S26. RDE measurement for ORR with various rotating speeds.** a-d) LSV curves of $Co_3S_4$, $Co_3S_4/MoS_2$, G-$Co_3S_4$, Pt/C (at 5 mV $s^{-1}$), e-h) their corresponding K-L plots.

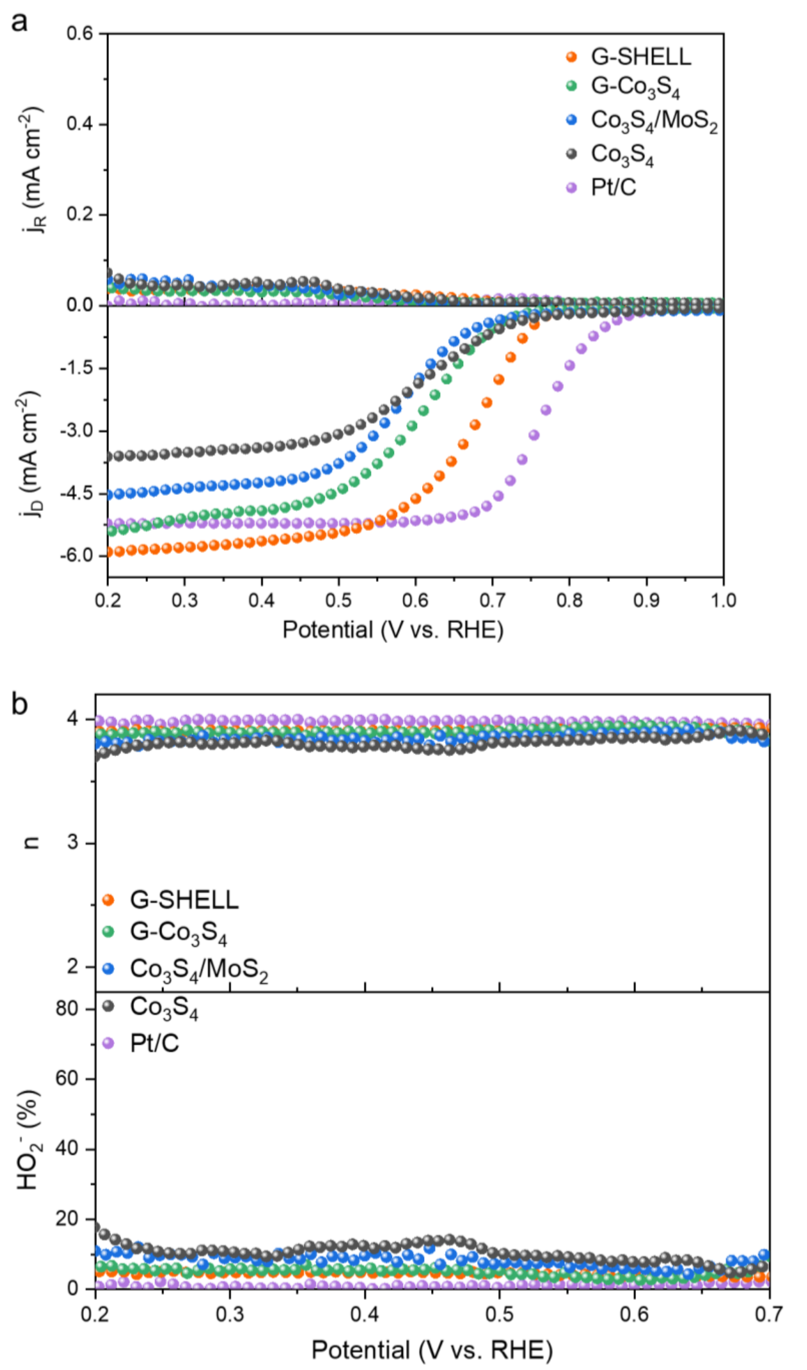


**Figure S27. Rotating-ring disk electrode (RRDE) measurement for calculating the electron transfer number (n) and hydrogen peroxide ($HO_2^-$) percentage.** a) LSV curves of $Co_3S_4$, $Co_3S_4/MoS_2$, G-$Co_3S_4$, G-SHELL, and Pt/C, b) Ring current and disk current correlated properties (n and % of $HO_2^-$). The measurement was conducted in $O_2$-saturated 0.1 M KOH with a rotating speed of 1600 rpm at 5mV $s^{-1}$.

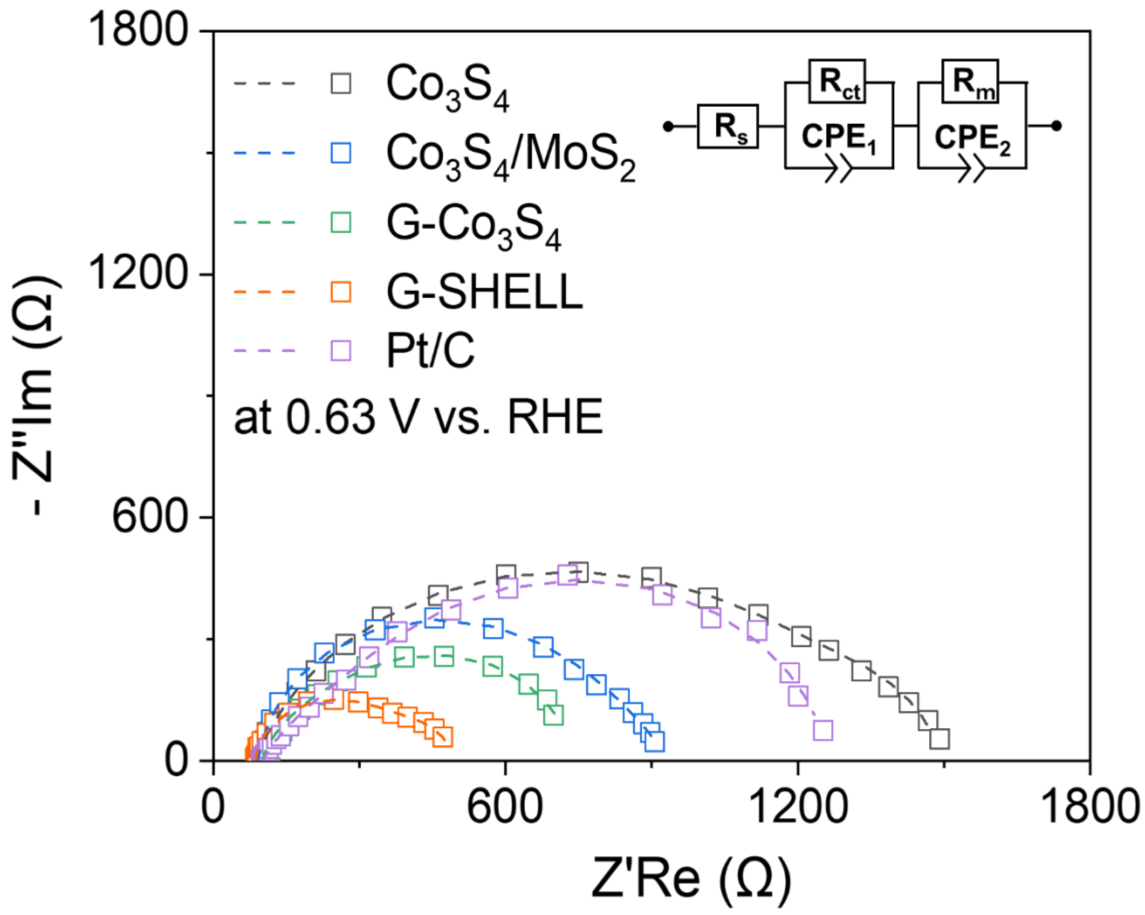


**Figure S28. Electrochemical impedance spectroscopy (EIS) spectra for ORR. The measurement was conducted in $O_2$-saturated 0.1 M KOH condition with a rotating speed of 1600 rpm.** The equivalent circuit model was inserted in the graph and the dashed lines indicate the fitted data.

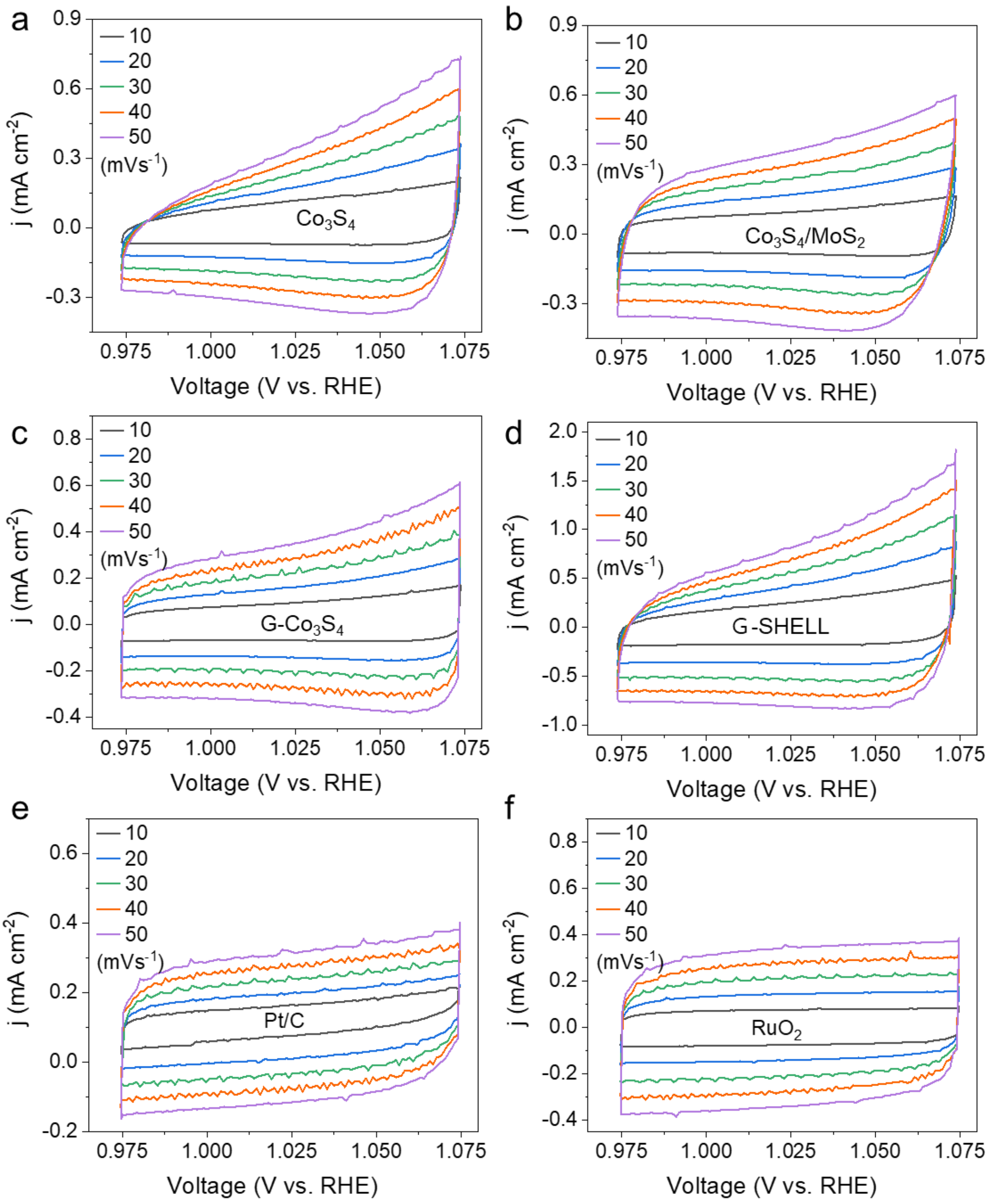


**Figure S29. CV spectra as a function of various scan rates.** a) $Co_3S_4$, b) $Co_3S_4/MoS_2$, c) G-$Co_3S_4$, d) G-SHELL, e) Pt/C, and f) $RuO_2$.

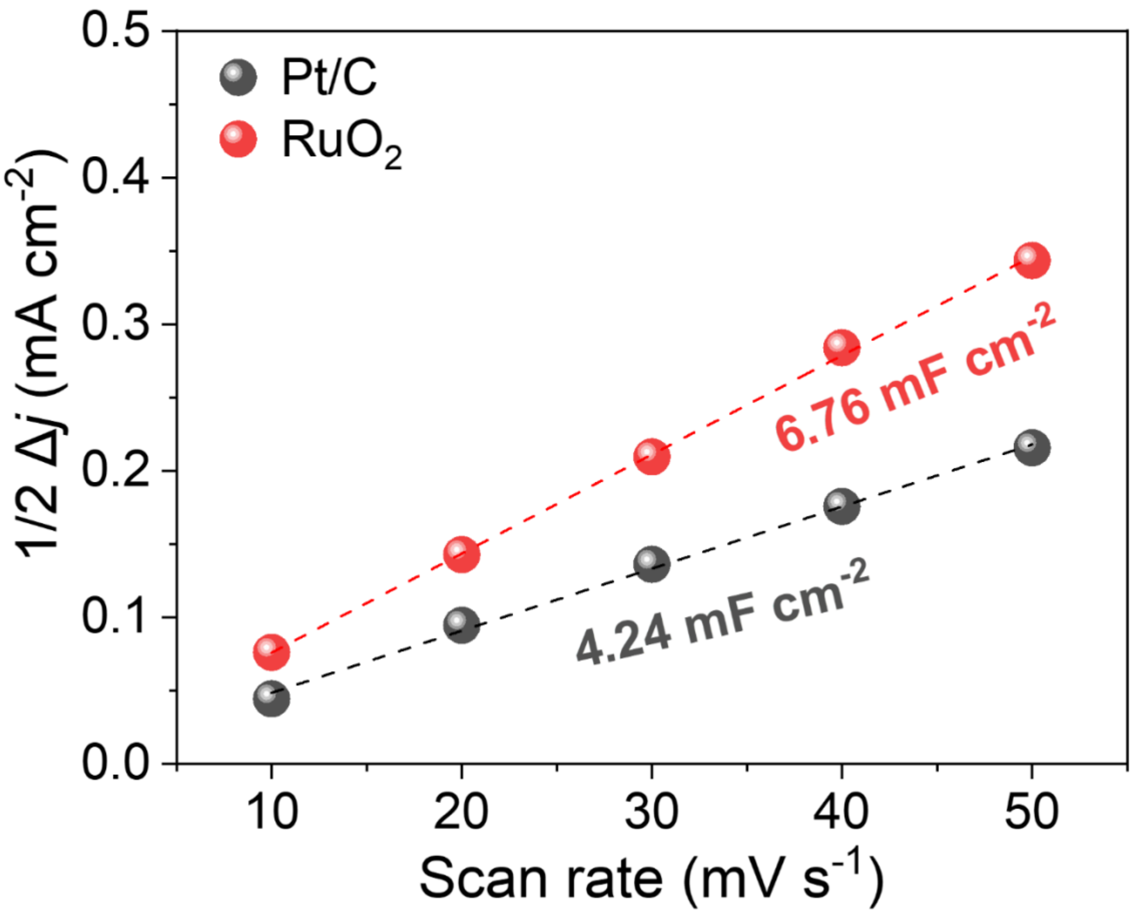


**Figure S30. Electric double layer capacitance calculation from the slope of 1/2Δ*j*-scan rate plot. (Δj is the current density difference between anodic and cathodic currents at 1.025 V vs. RHE in Figure S29e and S29f).**

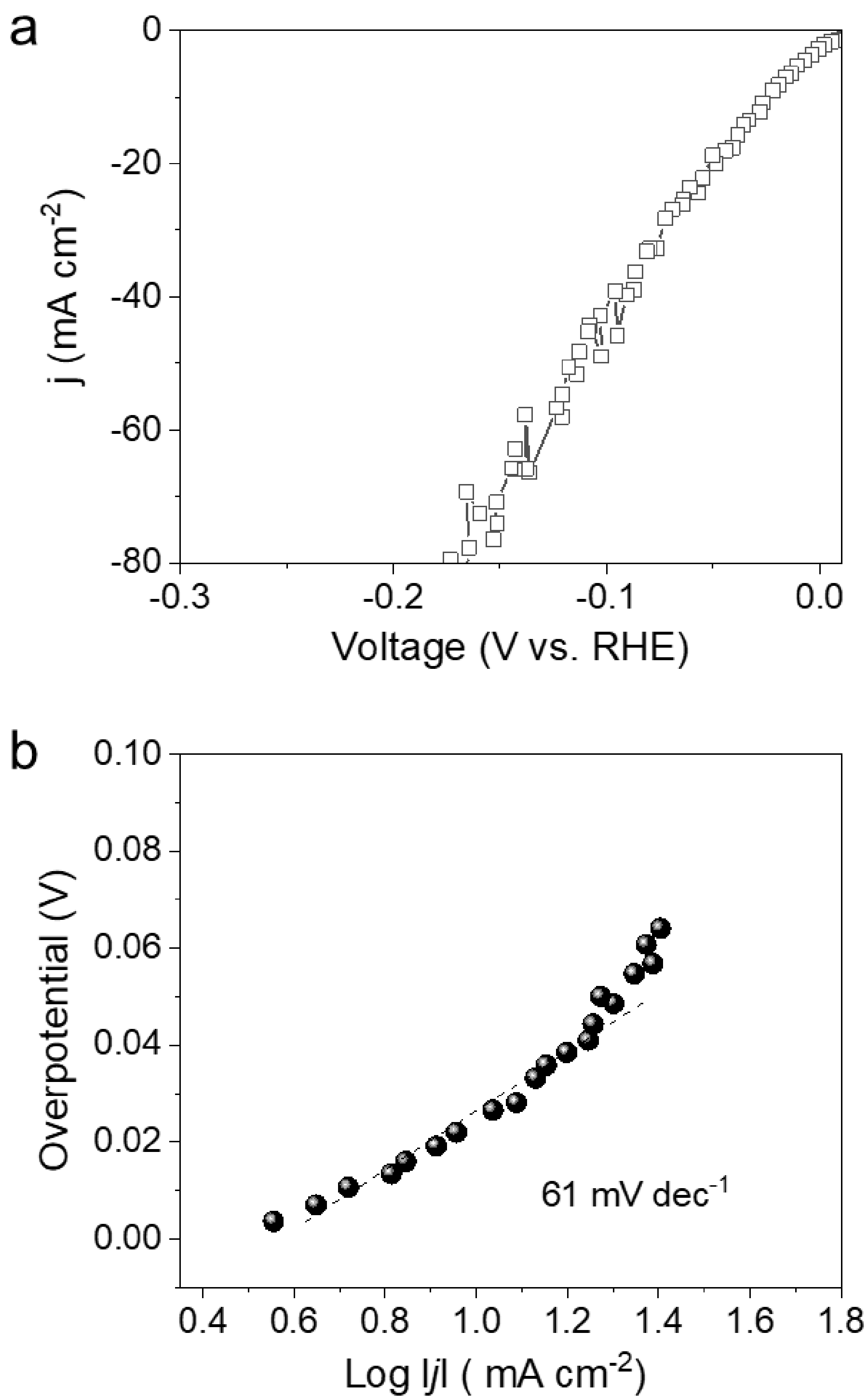


**Figure S31. HER performance of Pt/C.** a) LSV curves and b) Tafel plot.

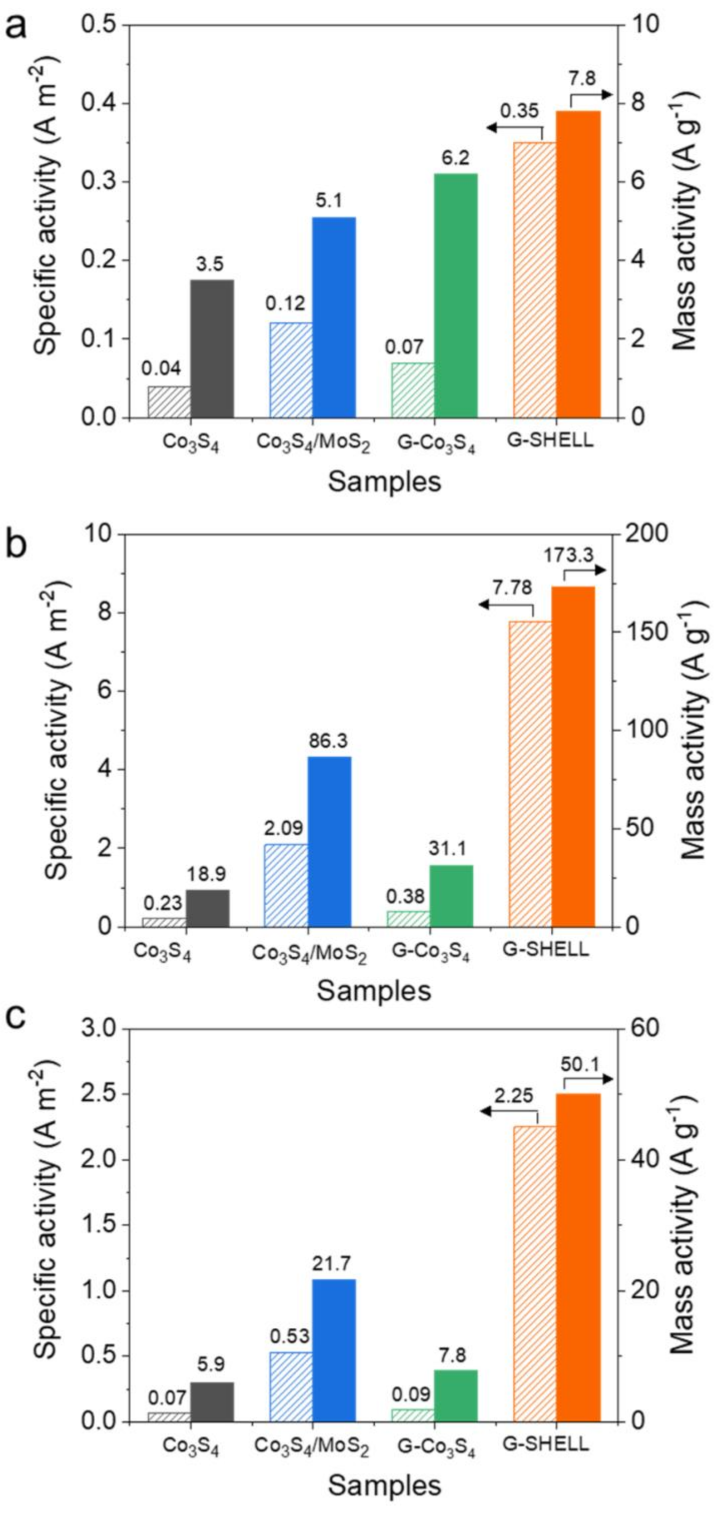


**Figure S32. Mass activity and Specific activity for three different reactions, current density values were calculated from the RDE electrode at 1600 rpm in Figure 4.** a) ORR at 0.6 V vs.RHE, b) OER at 1.6 V vs.RHE, and c) HER at -0.27 V vs.RHE.

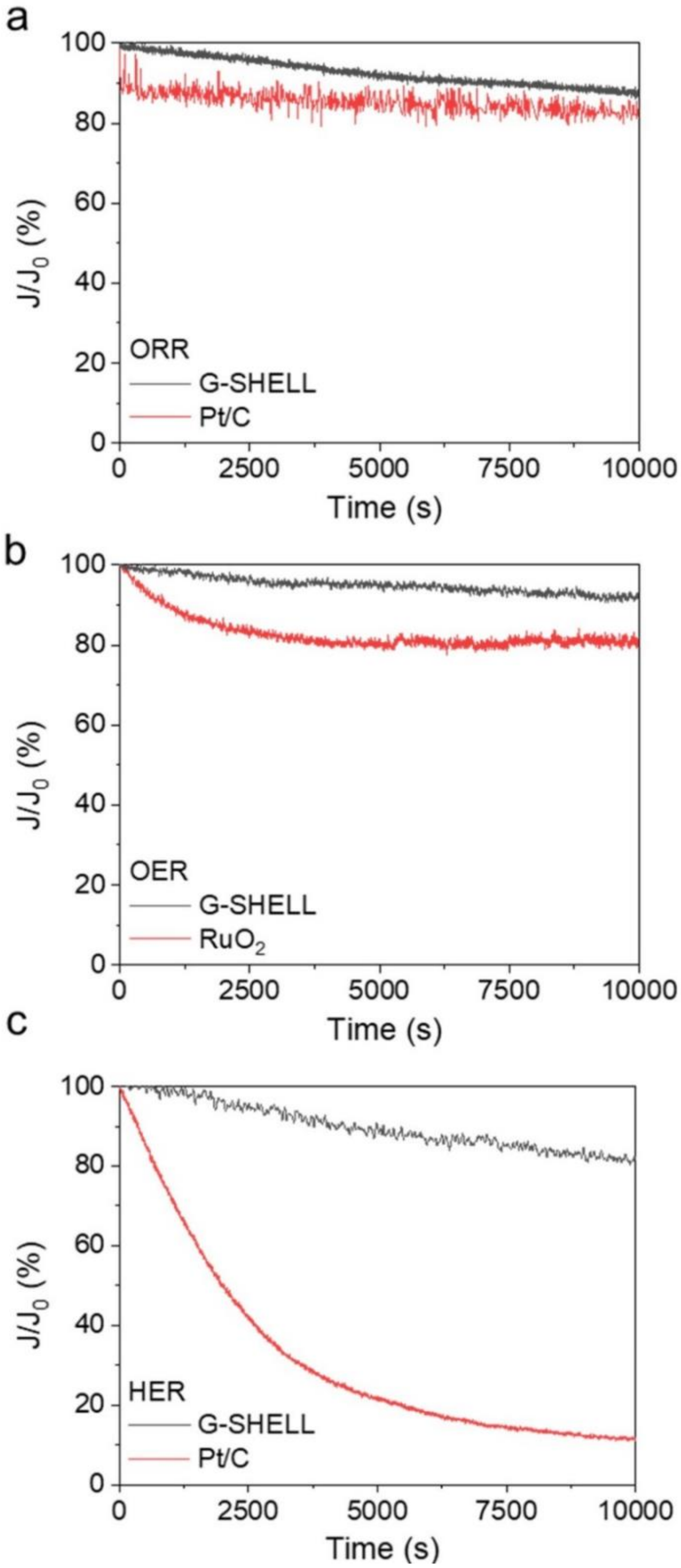


**Figure S33. Chronoamperometry-based stability tests of G-SHELL and noble metal electrocatalysts using RDE electrodes at 400 rpm for** a) ORR, b) OER, and c) HER.

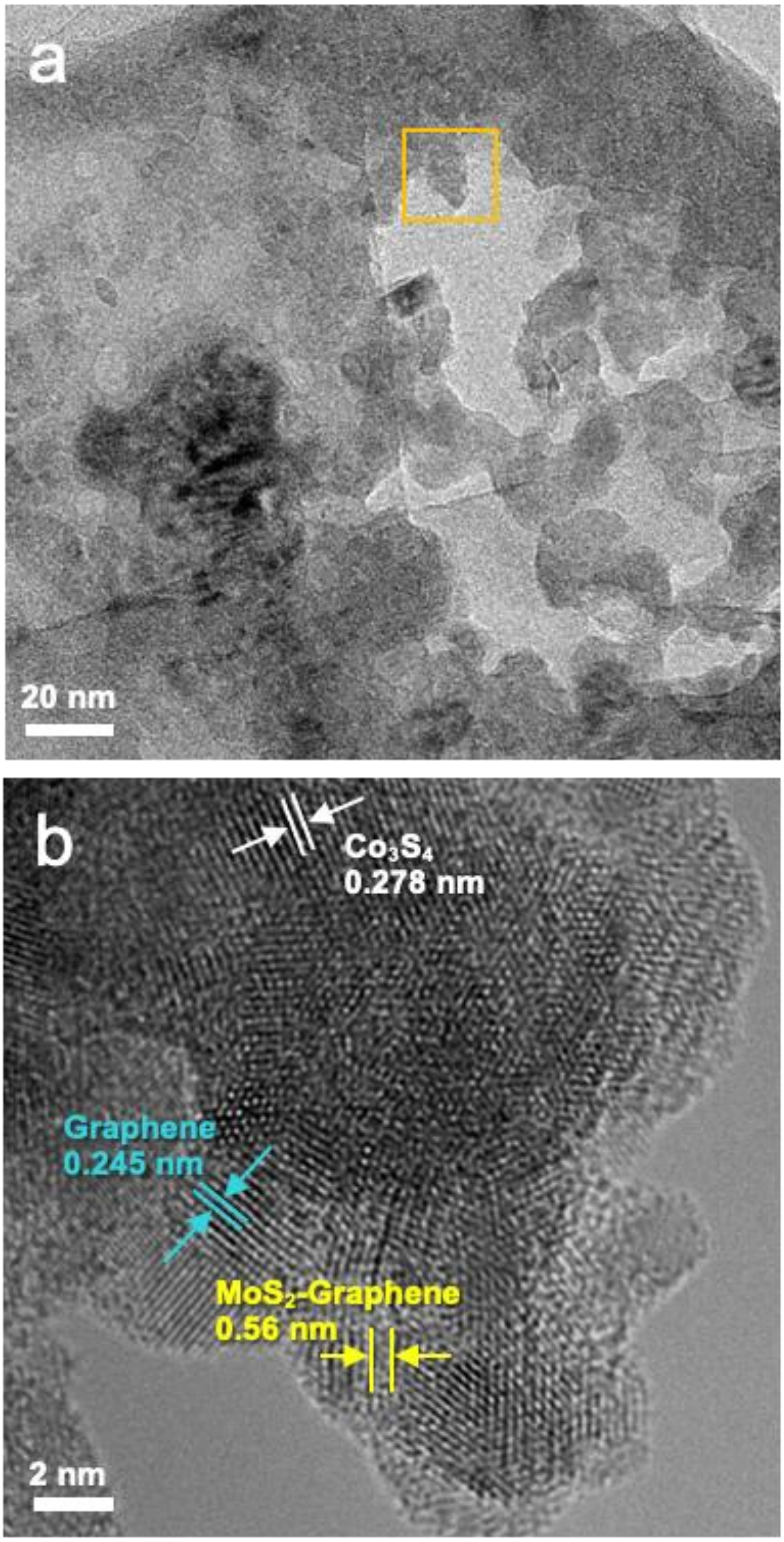


**Figure S34. Structural characterization of G-SHELL after immersing in 1 M KOH for 10 days.** a) TEM images and b) enlarged marked region.

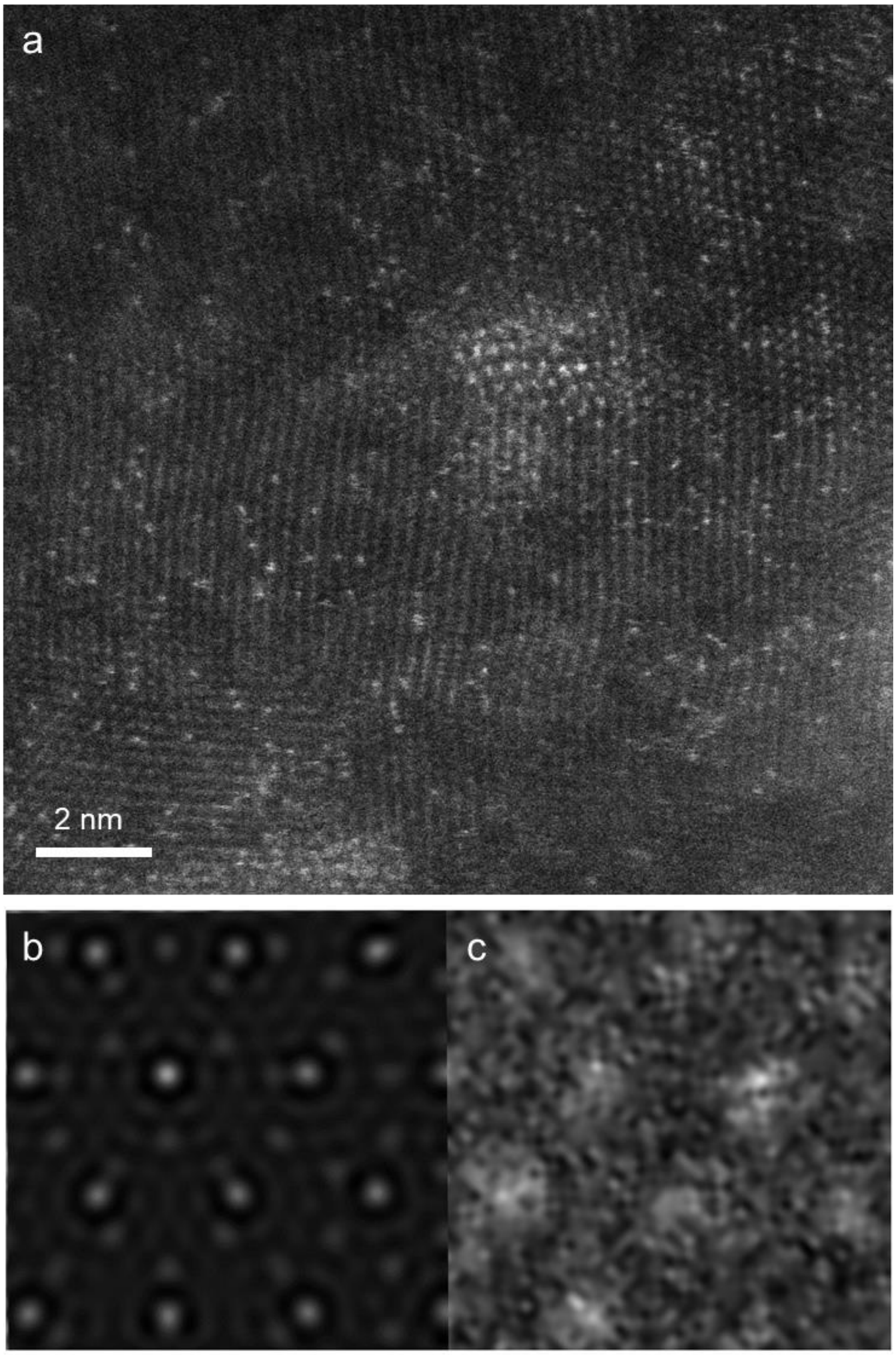


**Figure S35. Structural verification of G-SHELL after ORR durability test over 100 h.** a) STEM image of G-SHELL after HER durability test, b) Simulated STEM image of $MoS_2$/Graphene heterostructure, and c) atomic STEM image of G-SHELL after ORR durability test. The length and width of images b) and c) are 1 nm x 1 nm.

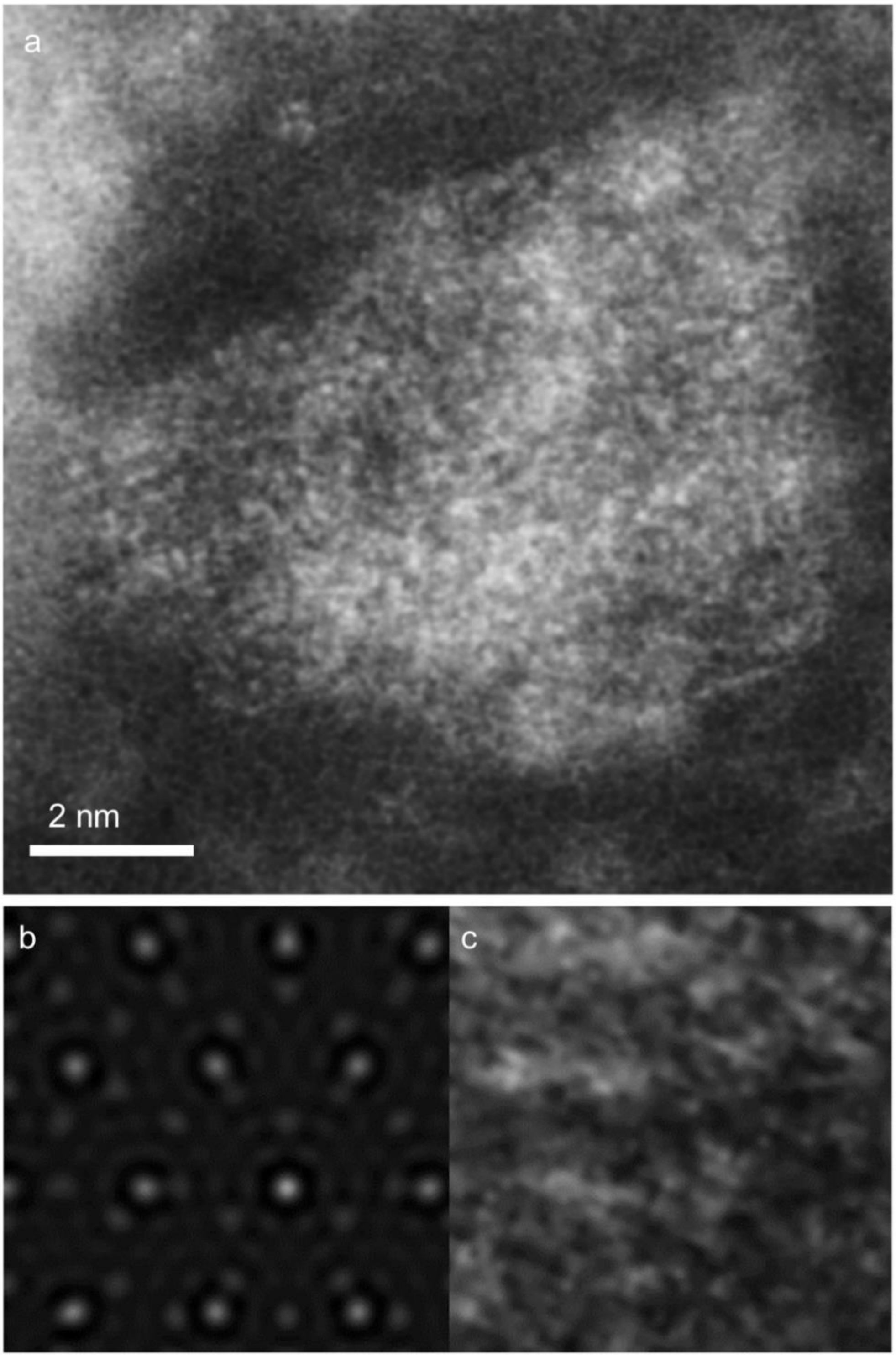


**Figure S36. Structural verification of G-SHELL after OER durability test over 100 h.** a) STEM image of G-SHELL after OER durability test, b) Simulated STEM image of $MoS_2$/Graphene heterostructure, and c) atomic STEM image of G-SHELL after OER durability test. The length and width of images b) and c) are 1 nm x 1 nm.

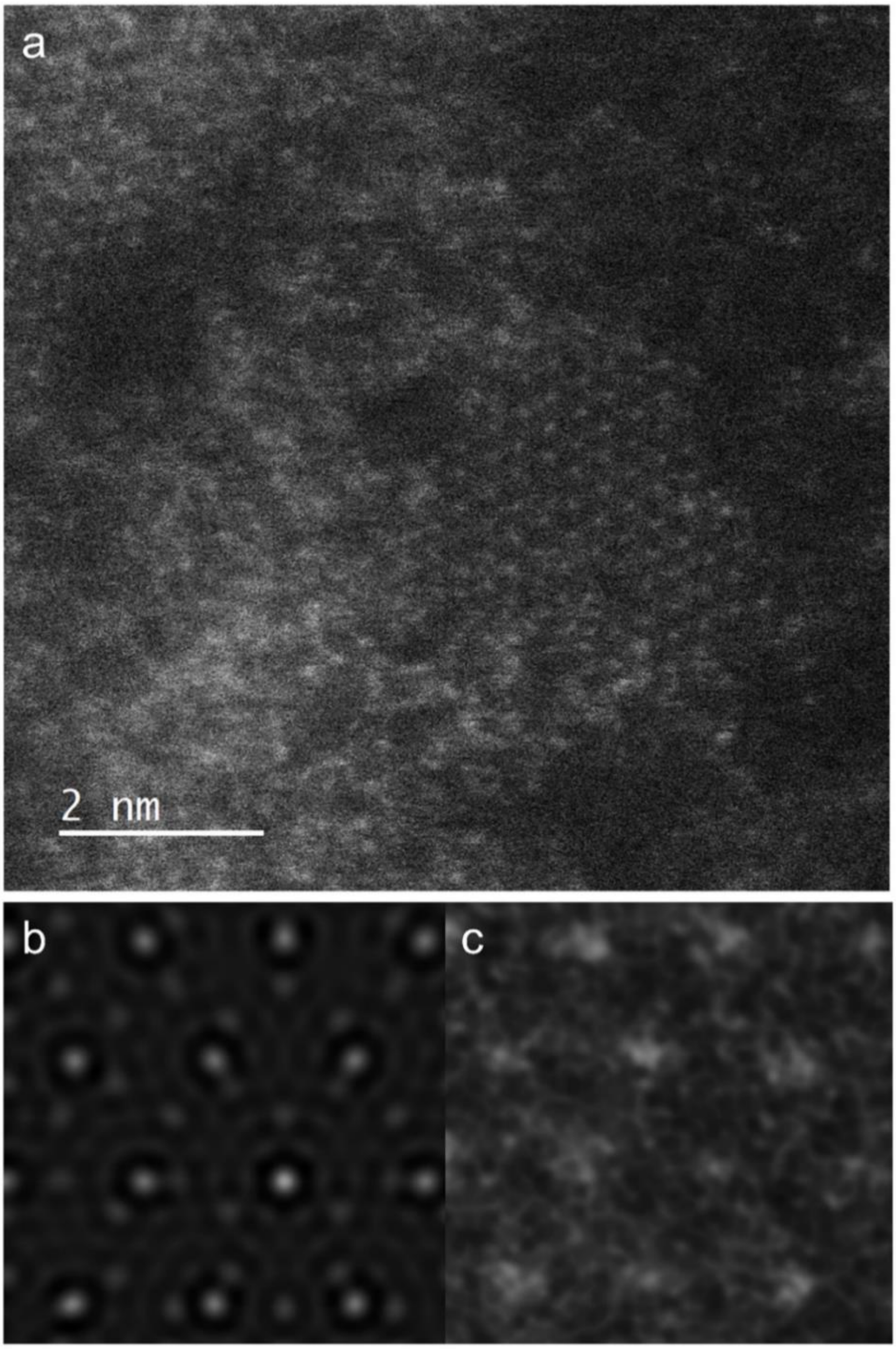


**Figure S37. Structural verification of G-SHELL after HER durability over 100 h.** a) STEM image of G-SHELL after HER durability test, b) Simulated STEM image of $MoS_2$/Graphene heterostructure, and c) atomic STEM image of G-SHELL after HER durability test. The length and width of images b) and c) are 1 nm x 1 nm.

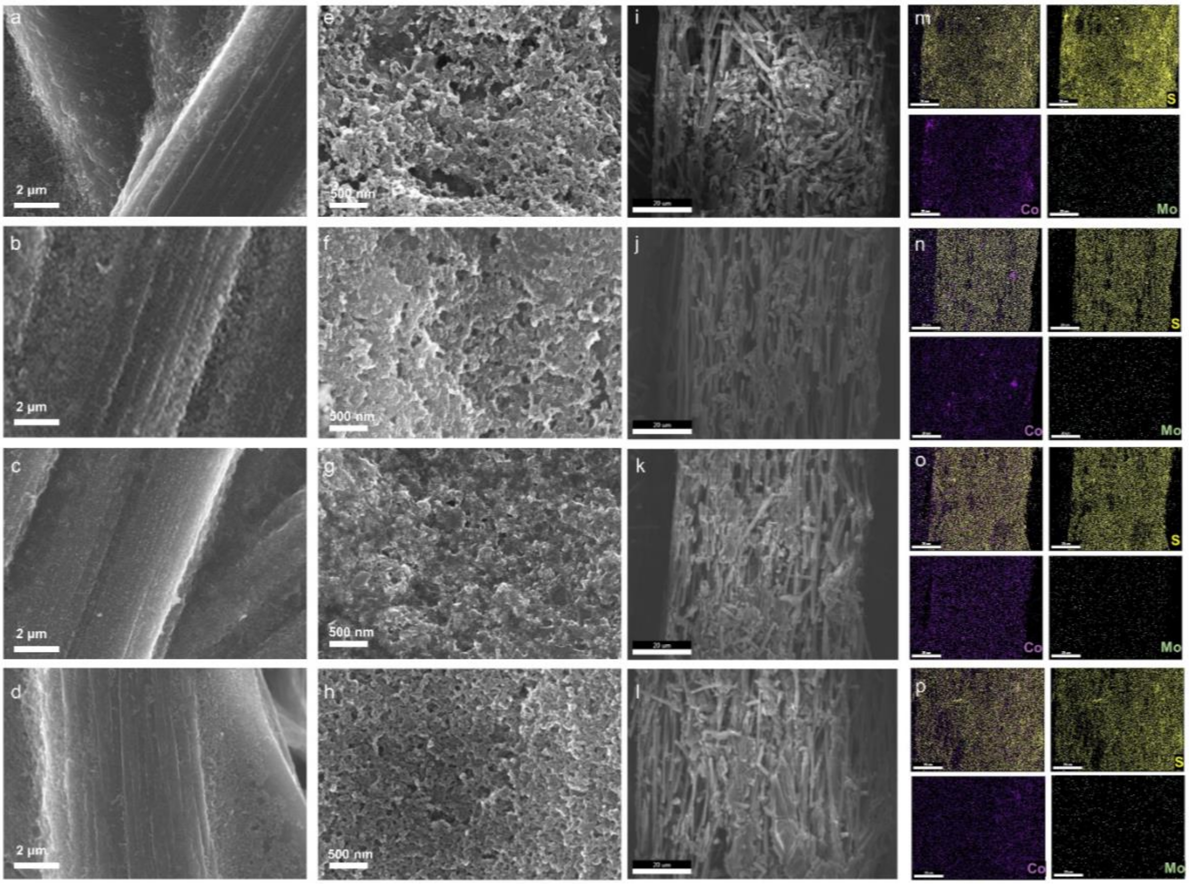


**Figure S38. SEM images of the electrode surface and cross-sectional images of SEM-EDS after durability tests.** a-d) low magnification SEM images, e-h) high-magnification SEM images, i-l) EDS-mode images, and m-p) EDS mapping of electrodes consisting of G-SHELL, G-SHELL after the ORR durability test, G-SHELL after the OER durability test, and G-SHELL after the HER durability test.

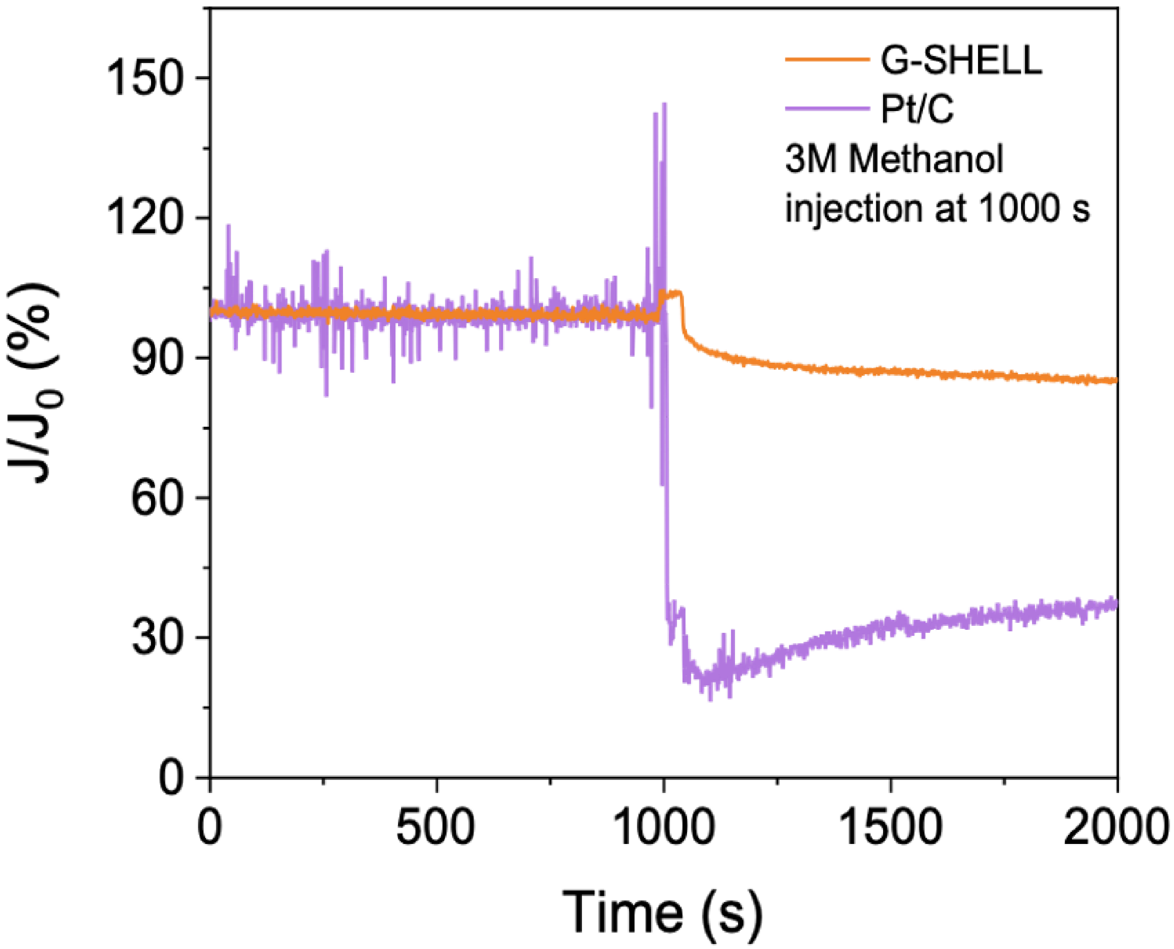


**Figure S39. Methanol tolerance estimation at 0.6 V vs. RHE under $O_2$-saturated 0.1 M KOH solution.**

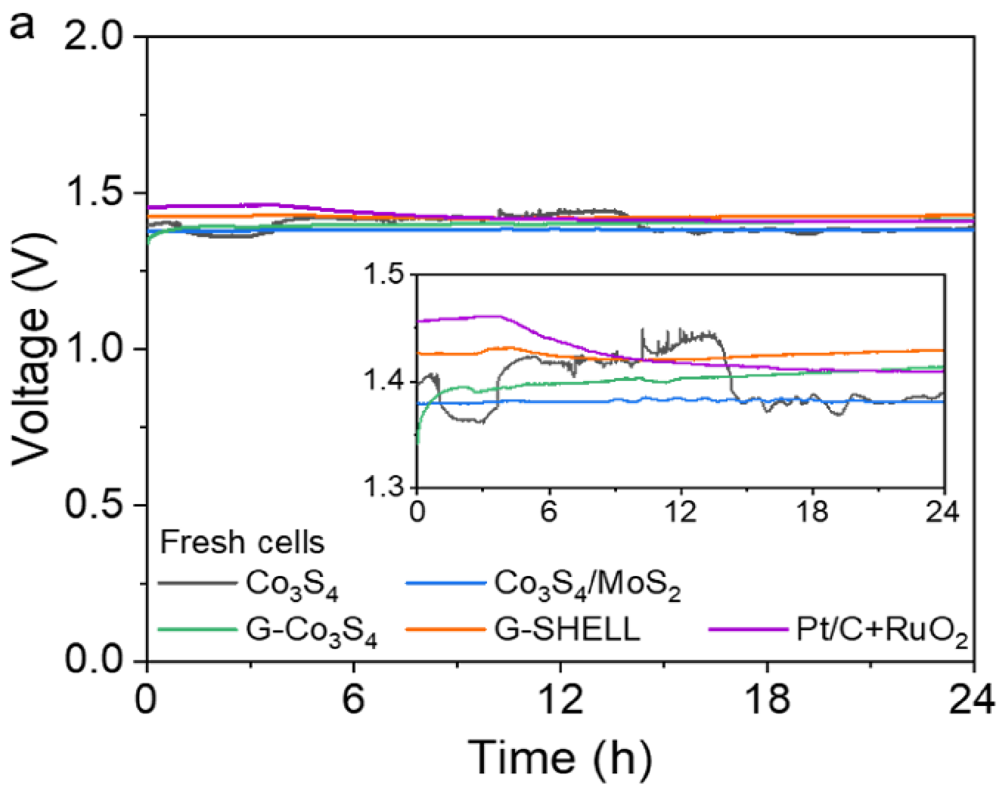


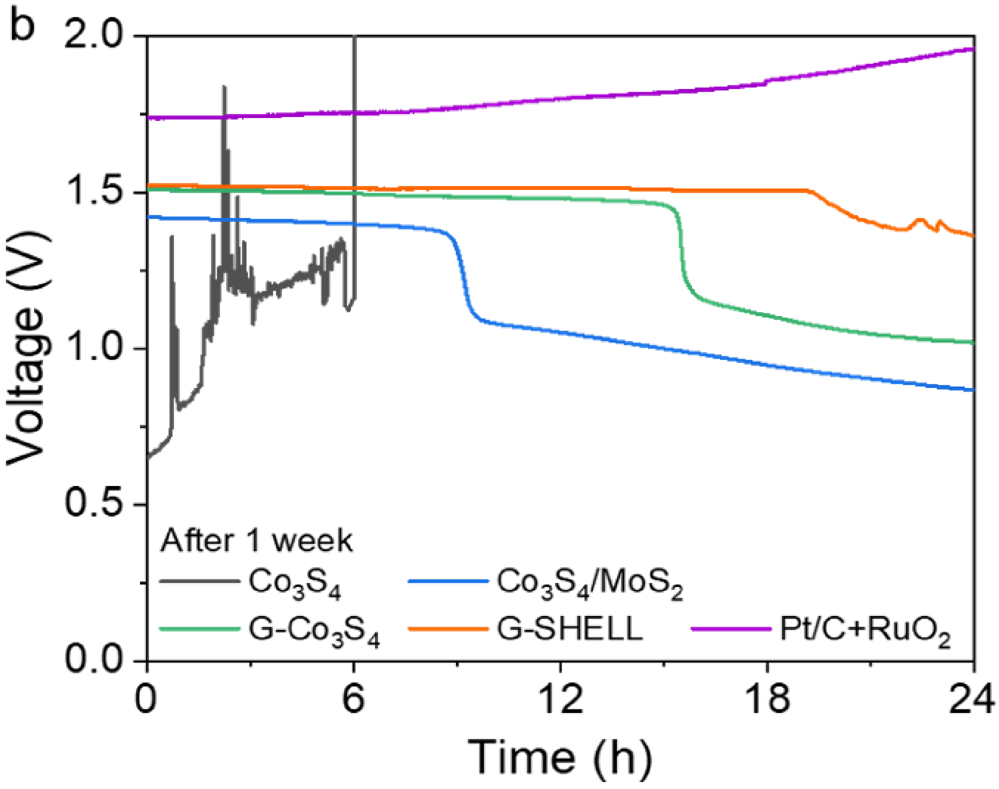


**Figure S40. Open circuit voltage (OCV) monitoring of Zn-air batteries (ZABs) in the ambient condition with 6 M KOH + 0.2 M Zn(OAC)$_2$ electrolyte for stack cells.** Those a) during the first 24 hours and b) after 1 week(168 hours).

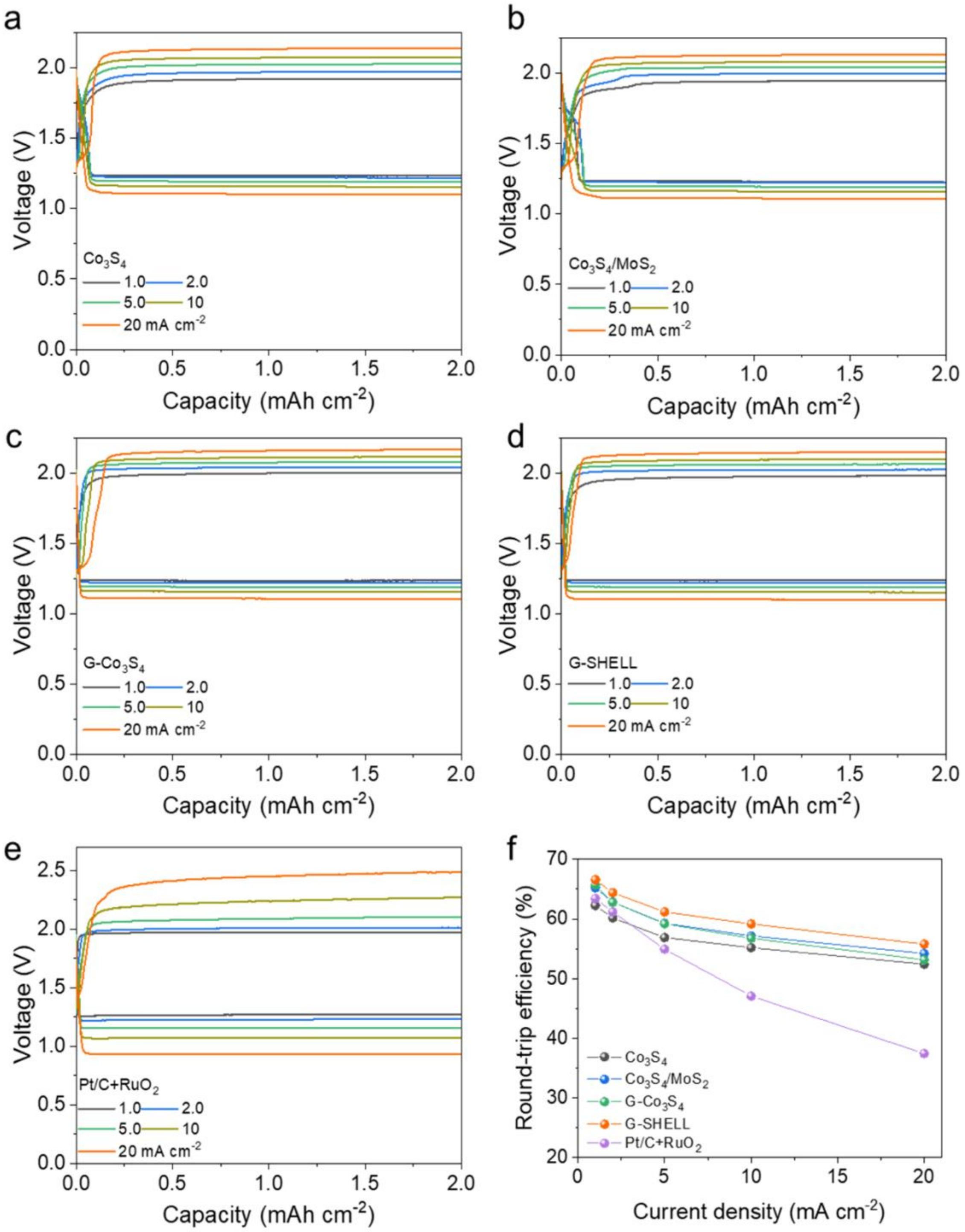


**Figure S41. Rate stability of ZABs under the ambient condition with 6 M KOH + 0.2 M $Zn(OAC)_2$ electrolyte for stack cells.** Rate stability behaviors of a) $Co_3S_4$, b) $Co_3S_4/MoS_2$, c) G-$Co_3S_4$, d) G-SHELL, and e) Pt/C+$RuO_2$, and f) their round-trip efficiency behaviors.

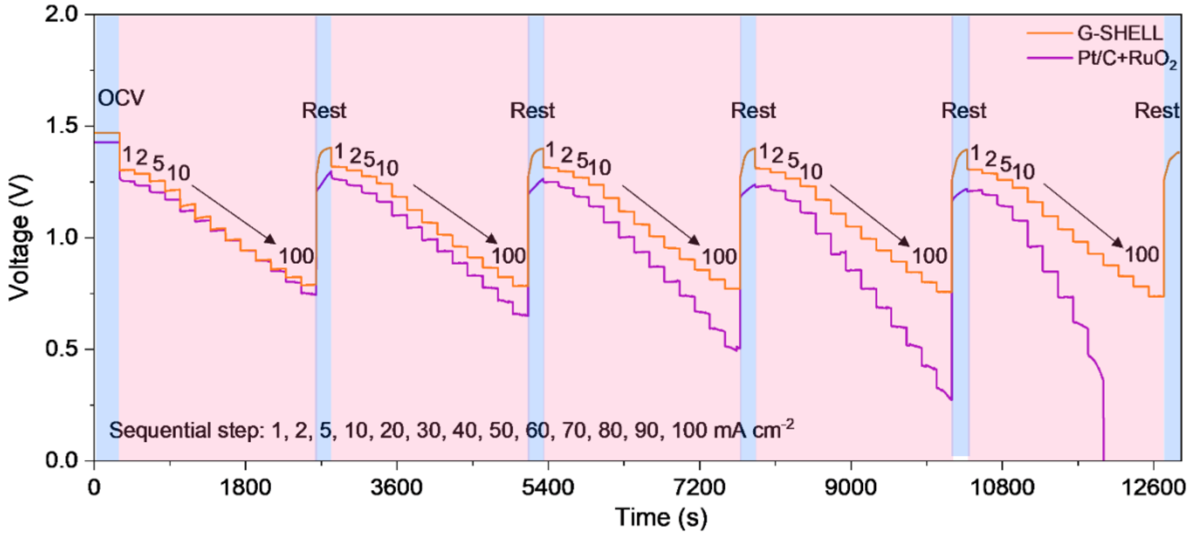


**Figure S42. The rate capability of ZABs under repeated discharge and resting cycles with the air electrodes of G-SHELL and Pt/C+$RuO_2$.**

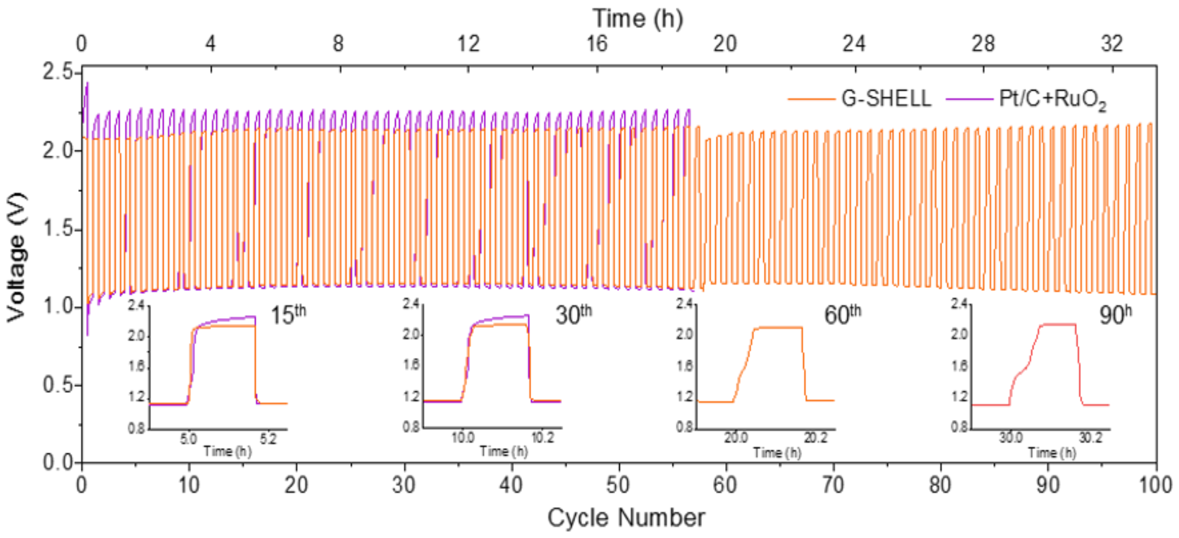


**Figure S43. Long-term cyclability test of ZABs at 10 mA cm$^{-2}$ with 20 min intervals.**

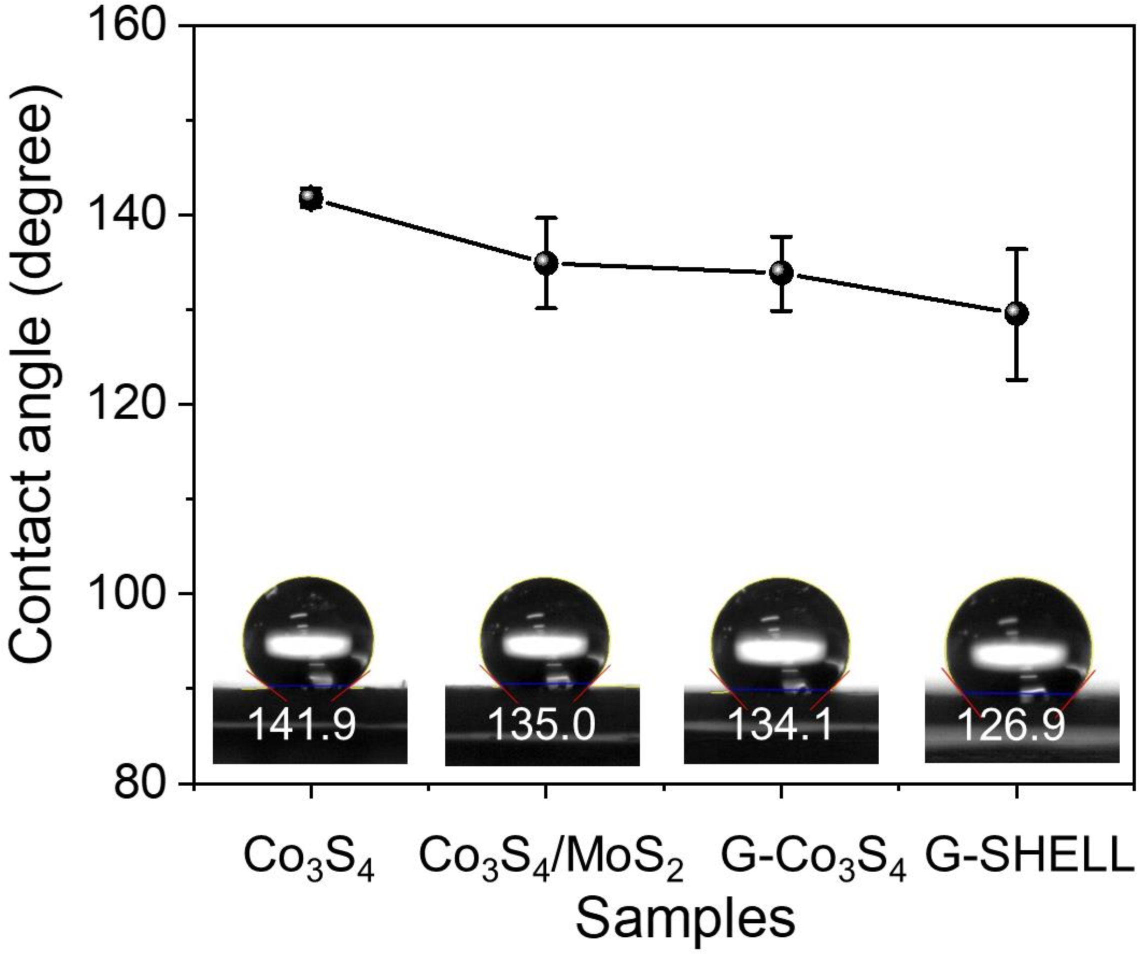


**Figure S44. Contact angle measurement for hydrophilicity analysis using a catalyst ink drop on a hydrophobic gas diffusion layer with a DI-water droplet on $Co_3S_4$, $Co_3S_4/MoS_2$, G-$Co_3S_4$, and G-SHELL.**

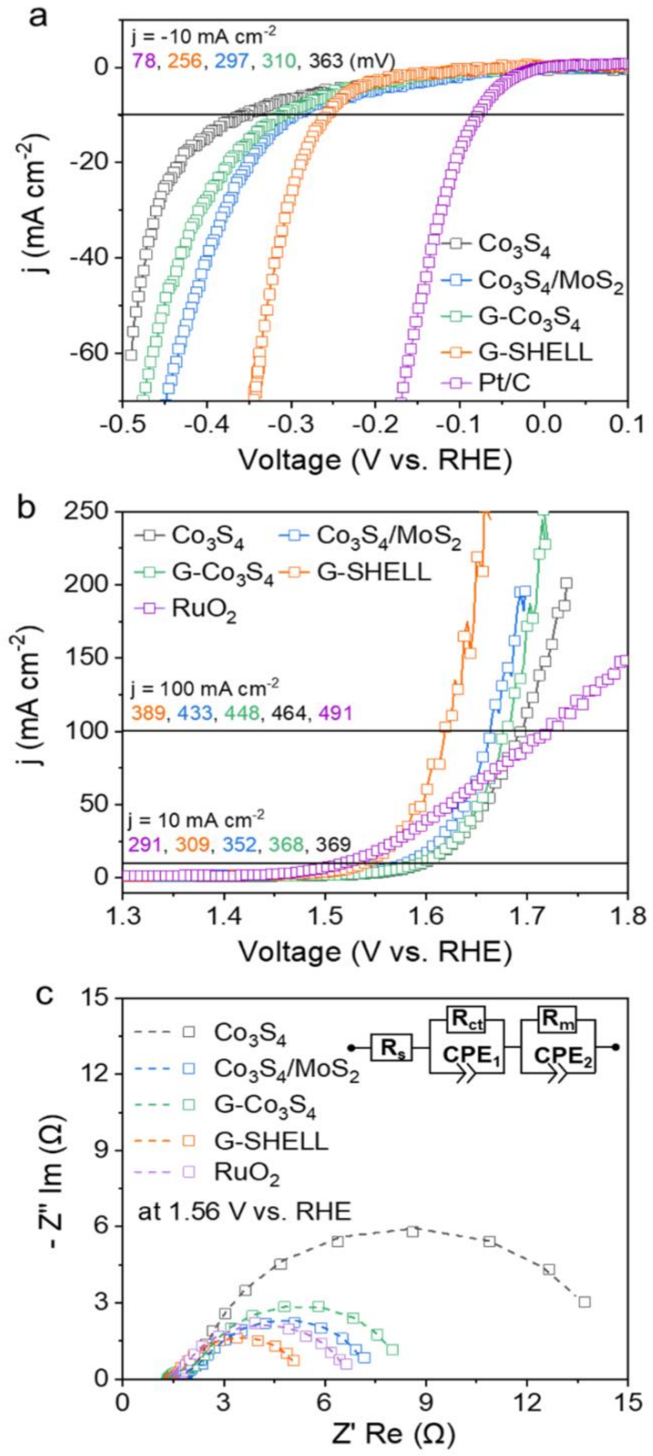


**Figure S45. Water-splitting performances of $Co_3S_4$, $Co_3S_4/MoS_2$, G-$Co_3S_4$, G-SHELL, Pt/C, and $RuO_2$ coated on a hydrophilic carbon substrate in $N_2$-saturated 1 M KOH solution.** a) HER, b) OER, and c) EIS performances at 1.56 V vs. RHE. The equivalent circuit model was inserted in the graph and dashed lines indicate the fitted data.

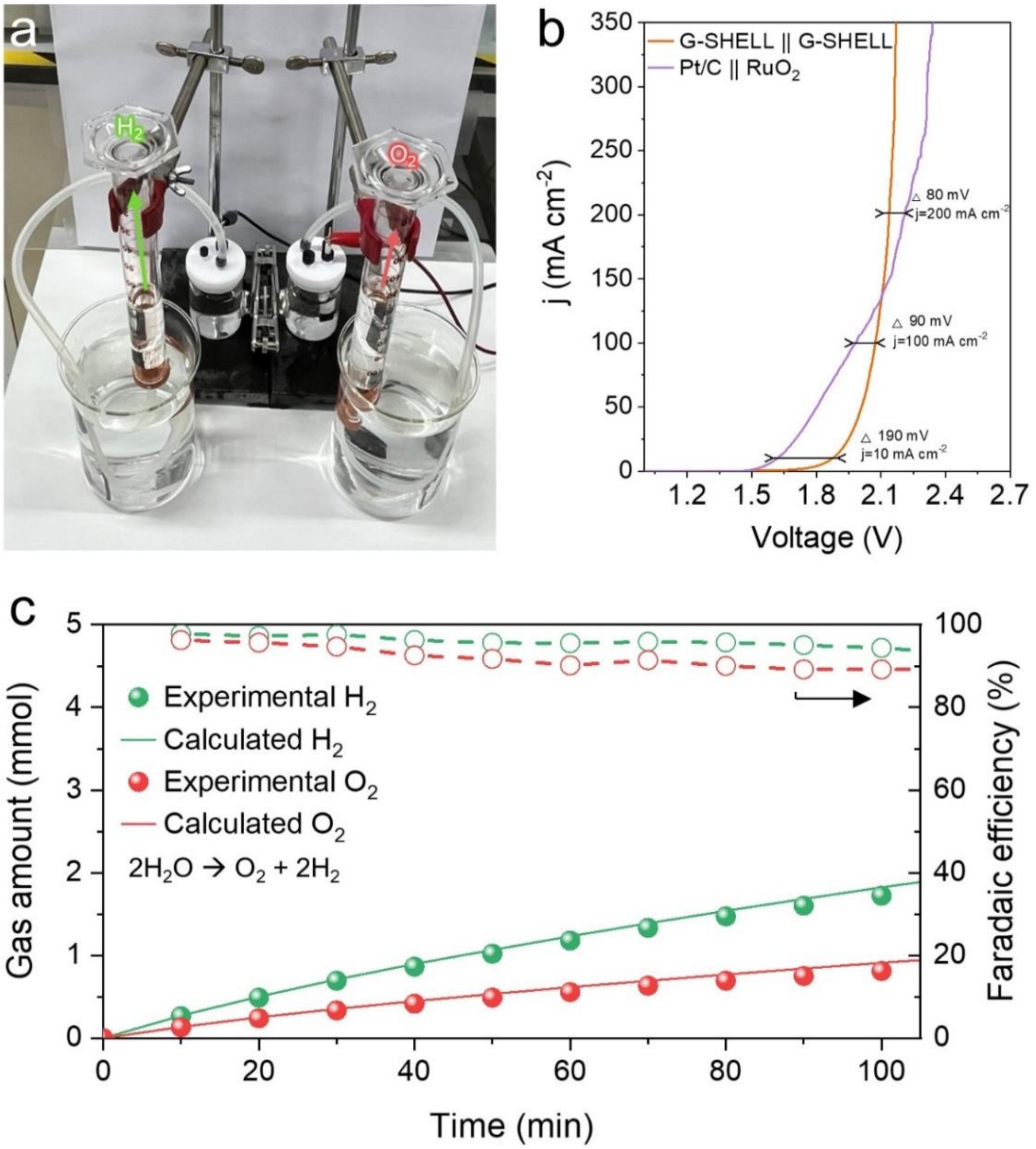


**Figure S46. Faradaic efficiency measurements.** a) optical images of home-made two-electrode water splitting and gas collection device by measuring water-displacement, b) LSV curves of symmetric G-SHELL ‖ G-SHELL and noble metal-based Pt/C ‖ $RuO_2$. c) the amount of collected gases at a constant voltage of 2.1 V.

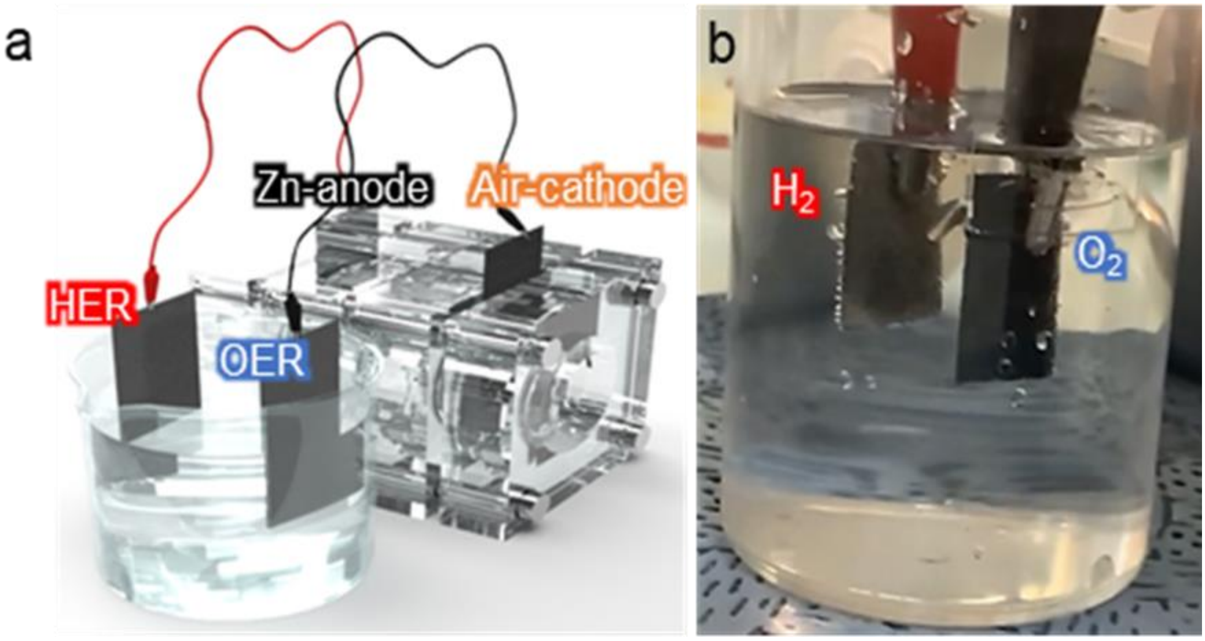


**Figure S47. Self-powered hydrogen production system with G-SHELL electrocatalysts for HER, OER, and ORR.** a) Illustration of a ZAB-driven water-splitting cell and b) snapshot during the electrolysis process.

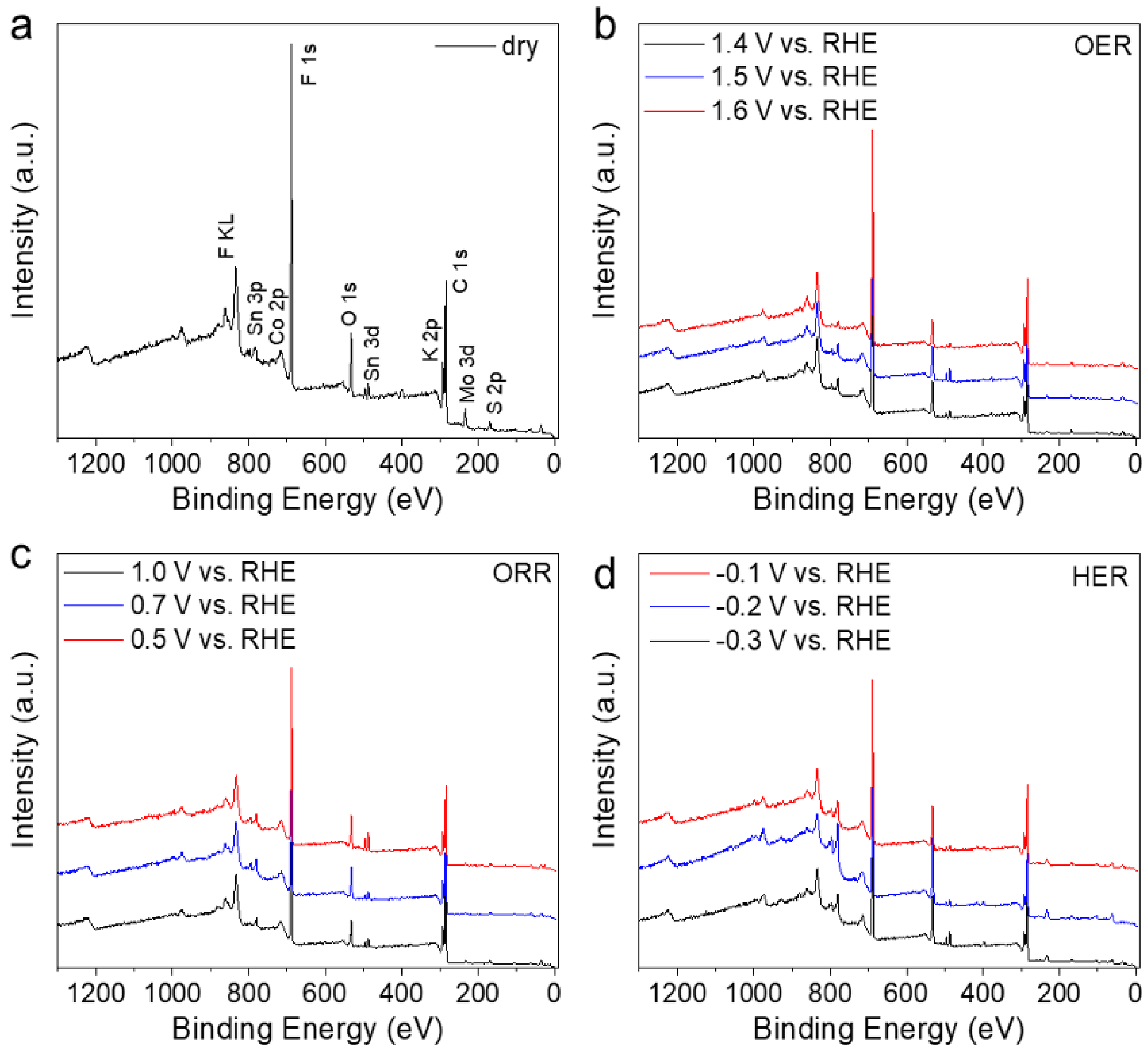


**Figure S48. *Ex-situ* XPS of three different potential regions with the fluorine-doped tin oxide (FTO) substrate.** XPS spectra of the electrodes (a) before each reaction, after (b) OER, (c) ORR, and (d) HER.

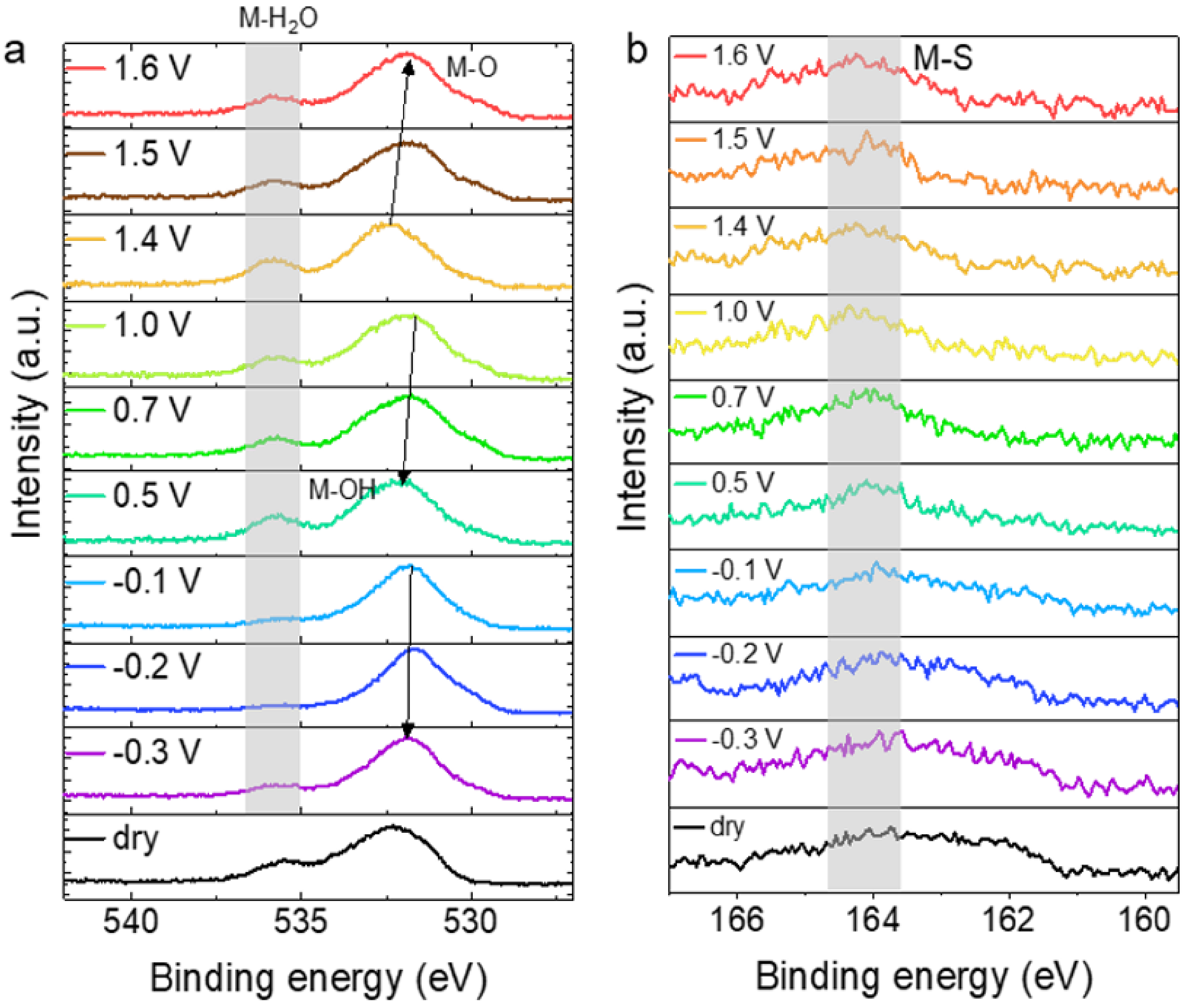


**Figure S49. *Ex-situ* XPS of three different potential regions for OER, ORR, and HER with the G-SHELL-coated FTO substrate.** XPS spectra of (a) O 1s and (b) S 2p.

**Table S1. Results for EXAFS fitting of Mo K edge analysis.**

| Samples | Path | Coordination number | Bond length (Å) | Bond disorder ($\sigma^2$) | R-factor of fit |
|---|---|---|---|---|---|
| Mo | Mo-Mo | 7.961 | 2.72717 | 0.00807 | 0.04656 |
| | Mo-Mo | 5.840 | 3.01148 | 0.00689 | |
| | Mo-Mo | 12.479 | 4.25158 | 0.00078 | |
| | Mo-Mo | 31.337 | 5.26681 | 0.00258 | |
| $MoO_3$ | Mo-O | 4.162 | 1.92873 | 0.00754 | 0.02627 |
| | Mo-O | 0.975 | 2.13093 | 0.00232 | |
| | Mo-Mo | 1.876 | 3.24489 | 0.00649 | |
| | Mo-O | 5.900 | 3.48932 | 0.00994 | |
| | Mo-O | 9.528 | 3.91132 | 0.00172 | |
| | Mo-Mo | 4.130 | 3.97492 | 0.00185 | |
| $MoS_2$ | Mo-S | 6.075 | 2.36955 | 0.00036 | 0.03608 |
| | Mo-Mo | 6.013 | 3.07707 | 0.00020 | |
| | Mo-S | 6.893 | 4.21504 | 0.00281 | |
| $Co_3S_4/MoS_2$ | Mo-S | 5.980 | 1.93488 | 0.00159 | 0.0488864 |
| | Mo-Mo | 5.985 | 3.36896 | 0.00247 | |
| | Mo-S | 6.000 | 4.01737 | 0.00323 | |
| | Mo-S | 6.213 | 4.76735 | 0.00303 | |
| G-SHELL | Mo-S | 6.030 | 2.32409 | 0.00501 | 0.0281265 |
| | Mo-Mo | 6.018 | 3.10931 | 0.00015 | |
| | Mo-S | 6.030 | 4.09086 | 0.00173 | |
| | Mo-C | 10.009 | 4.66713 | 0.00025 | |
| | Mo-S | 12.059 | 5.47879 | 0.00606 | |

**Table S2. Results for EXAFS fitting of Co K edge analysis.**

| Samples | Path | Coordination number | Bond length (Å) | Bond disorder ($\sigma^2$) | R-factor of fit |
|---|---|---|---|---|---|
| Co | Co-Co | 11.805 | 2.49711 | 0.00709 | 0.05166 |
| | Co-Co | 6.309 | 3.58932 | 0.00975 | |
| | Co-Co-Co | 48.787 | 3.62355 | 0.00853 | |
| | Co-Co-Co | 49.093 | 4.45469 | 0.00163 | |
| $Co_3S_4/MoS_2$ | Co-S | 4.017 | 2.37313 | 0.00240 | 0.02500 |
| | Co-Co | 12.123 | 3.71560 | 0.00757 | |
| | Co-S | 11.788 | 3.76559 | 0.00062 | |
| | Co-S-S | 50.601 | 4.72820 | 0.00863 | |
| G-SHELL | Co-S | 4.061 | 2.24346 | 0.00051 | 0.02824 |
| | Co-Co | 13.797 | 3.77284 | 0.00108 | |
| | Co-S | 12.016 | 3.91105 | 0.00638 | |
| | Co-S-Co | 24.732 | 4.28783 | 0.00256 | |

**Table S3. Comparison of onset potential, half-wave potential, kinetic current density, electron transference number, and hydrogen peroxide values under ORR.**

| Electrocatalyst | Onset potential ($E_{onset}$, V) | Half-wave potential ($E_{1/2}$, V) | Kinetic current density ($j_k$, mA $cm^{-2}$) | Electron transference number (n) | Hydrogen peroxide ($HO_2^-$, %) |
|---|---|---|---|---|---|
| $Co_3S_4$ | 0.82 | 0.64 | 4.5 | 3.83 | 8.58 |
| $Co_3S_4/MoS_2$ | 0.85 | 0.67 | 7.3 | 3.88 | 5.69 |
| G-$Co_3S_4$ | 0.87 | 0.71 | 10.4 | 3.92 | 3.51 |
| G-SHELL | 0.89 | 0.72 | 10.5 | 3.95 | 2.53 |
| Pt/C | 0.93 | 0.81 | 10.7 | 3.97 | 1.37 |

**Table S4. Comparison of the diffusion-limited current density ($j_L$ at 0.2 V vs. RHE) and EIS fitted parameters (at 0.63 V vs. RHE), including solution resistance ($R_s$), charge transfer resistance ($R_{ct}$), and mass transfer resistance ($R_m$) values under ORR. The EIS fitted parameter was calculated from Figure S28.**

| Electrocatalyst | Diffusion-limited current density ($j_L$, mA cm$^{-2}$) | Solution resistance ($R_s$, Ω) | Charge transfer resistance ($R_{ct}$, Ω) | Mass transfer resistance ($R_m$, Ω) |
|---|---|---|---|---|
| $Co_3S_4$ | 2.5 | 86.1 | 1235 | 171.8 |
| $Co_3S_4/MoS_2$ | 3.0 | 93.7 | 585 | 243.3 |
| G-$Co_3$S4 | 3.3 | 98.6 | 368.6 | 271 |
| G-SHELL | 3.9 | 85.6 | 290.8 | 113.3 |
| Pt/C | 3.0 | 100.4 | 903.6 | 259.8 |

**Table S5. Double-layer capacitance, HER overpotential, HER Tafel slope, OER overpotential, and OER Tafel slope values of recently reported alkaline water splitting electrocatalysts and this work.**

| Electrocatalyst | Double-layer capacitance ($C_{dl}$, mF $cm^{-2}$) | HER over-potential ($\eta_{10}$, mV) | HER Tafel Slope (mv $dec^{-1}$) | OER over-potential ($\eta_{10}$, mV) | OER Tafel Slope (mv $dec^{-1}$) | Reference |
|---|---|---|---|---|---|---|
| $Co_3S_4$ | 6.19 | 367 | 101 | 372 | 48.1 | This work |
| $Co_3S_4/MoS_2$ | 6.54 | 270 | 124 | 334 | 63.3 | |
| G-$Co_3S_4$ | 7.18 | 330 | 105 | 358 | 72.2 | |
| G-SHELL | 14.3 | 220 | 110 | 320 | 55.8 | |
| $RuO_2$ | 6.76 | | | 354 | 96.5 | |
| $Co(OH)_2$/Pt(111) | | 248 | | | | [7] |
| $NH_2$-BP | 0.27 | 290 | 63 | | | [8] |
| Co-COF@MOF | 6.42 | | | 328 | 43.23 | [9] |
| NiPc-Ni | 1.96 | | | 427 | 83.0 | [10] |
| $Co^{3+}$@3D-$Nb_2CTx$ NW | | 236 | 123 | 420 | 61.8 | [11] |
| HEMG-NPs | | 555 | 149 | 377 | 150 | [12] |
| $MoSe_2$-$Cu_2S$ (NHSs) | 7.35 | 350 | 110 | 264 | 63 | [13] |

**Table S6. Mass activity (MA) and specific activity (SA) for ORR, OER, and HER.**

| Electrocatalyst | Specific surface area ($S_{BET}$, $m^2\ g^{-1}$) | ORR at 0.6 V vs. RHE (SA, $A\ m^{-2}$) / (MA, $A\ g^{-1}$) | OER at 1.6 V vs. RHE (SA, $A\ m^{-2}$) / (MA, $A\ g^{-1}$) | HER at -0.27 V vs. RHE (SA, $A\ m^{-2}$) / (MA, $A\ g^{-1}$) |
|---|---|---|---|---|
| $Co_3S_4$ | 80.94 | 0.04 / 3.5 | 0.23 / 18.9 | 0.07 / 5.9 |
| $Co_3S_4/MoS_2$ | 41.31 | 0.12 / 5.1 | 2.09 / 86.3 | 0.53 / 21.7 |
| G-$Co_3S_4$ | 82.83 | 0.07 / 6.2 | 0.38 / 31.1 | 0.09 / 7.8 |
| G-SHELL | 22.28 | 0.35 / 7.8 | 7.78 / 173.3 | 2.25 / 50.1 |
| $RuO_2$ | 18.07 | - | 2.03 / 36.6 | - |
| Pt/C | 172.56 | 0.04 / 6.5 | - | 2.61 / 449.8 |

**Table S7. Performances of ZABs with different electrocatalysts.**

| Electrocatalyst | OCV (V) | Current density ($mA\ cm^{-2}$) | Discharge plateau (V) | Voltage polarization (V) | Round trip efficiency (%) |
|---|---|---|---|---|---|
| $Co_3S_4$ | 1.38 | 1 to 20 | 1.23 to 1.1 | 0.75 to 1.01 | 62 to 52 |
| $Co_3S_4/MoS_2$ | 1.38 | 1 to 20 | 1.23 to 1.1 | 0.66 to 0.94 | 65 to 54 |
| G-$Co_3S_4$ | 1.41 | 1 to 20 | 1.24 to 1.1 | 0.64 to 0.98 | 66 to 53 |
| G-SHELL | 1.43 | 1 to 20 | 1.24 to 1.1 | 0.61 to 0.86 | 67 to 56 |
| Pt/C+$RuO_2$ | 1.41 | 1 to 20 | 1.23 to 0.6 | 0.77 to 2.23 | 63 to 37 |

**Table S8. Performances of ZAB cells with different electrocatalysts.**

| Electrocatalyst | Current density ($mA\ cm^{-2}$) | Es ($Wh\ kg^{-1}_{Zn}$) | Reference |
|---|---|---|---|
| G-SHELL | 10 | 797 | This work |
| Pt/C+$RuO_2$ | 10 | 742 | |
| FeP/$Fe_2O_3$ | 5 | 517 | [14] |
| $LaCo_{0.8}Ru_{0.2}O_{3-\delta}$ | 5 | 554.2 | [15] |
| Nitride/N-$Ti_3C_2$ | 5 | 627 | [16] |
| S-LDH/NG | 5 | 772 | [17] |
| SA-PtCoF | 10 | 785 | [18] |
| Asy-NiFe | 25 | 685.1 | [19] |

**Table S9. EIS fitted parameters under OER (at 1.56 V vs. RHE), including solution resistance ($R_s$), charge transfer resistance ($R_{ct}$), and mass transfer resistance ($R_m$) values under ORR. The EIS fitted parameter was calculated from Figure S21.**

| Electrocatalyst | Solution resistance ($R_s$, Ω) | Charge transfer resistance ($R_{ct}$, Ω) | Mass transfer resistance ($R_m$, Ω) |
|---|---|---|---|
| $Co_3S_4$ | 1.495 | 11.53 | 1.543 |
| $Co_3S_4/MoS_2$ | 1.54 | 4.231 | 1.524 |
| G-$Co_3$S4 | 1.341 | 5.548 | 1.307 |
| G-SHELL | 1.474 | 3.323 | 0.449 |
| $RuO_2$ | 1.554 | 4.644 | 2.737 |

**Section S3. Supplementary references**


[1] M. G. Park, J. W. Choi, I. W. Ock, G. H. Kim, J. K. Kang, *Adv. Energy Mater.* 2021, **11**, 2003563.

[2] W. S. Hummers, R. E. Offeman, *J. Am. Chem. Soc.* 1958, **80**, 1399.

[3] J. Qin, S. Wang, X. Wang, *Appl. Catal. B; Environ*. 2017, **209**, 476-482.

[4] Y. Xue, Z. Zho, Y. Li, H. Liu, Y. Li, Small, 2017, **13**, 1700936.

[5] R. K. Singh, R. Devivaraprasad, T. Kar, A. Chakraborty, M. Neergat, *J. Electrochem. Soc.* 2015, **162**, F489-F498.

[6] A. J. Bard, L. R. Faulkner, *Electrochemical Methods: Fundamentals and Applications* 2000, **2**nd Edition, 1-1050.

[7] R. Subbaraman, D. Tripkovic, K.-C. Chang, D. Strmcnik, A. P. Paulikas, P. Hirunsit, M. Chan, J. Greeley, V. Stamenkovic, N. M. Markovic, *Nat. Mater*. 2012, **11**, 550-557.

[8] L. Shao, H. Sun. L. Miao, X. Chen, M. Han, J. Sun, S. Liu, L. Li, F. Cheng, J. Chen, *J. Mater. Chem. A* 2018, **6**, 2494-2499.

[9] Z. Guo, S. Yang, M. Liu, Q. Xu, G. Zeng, *Small* 2024, **20**, 2308598.

[10] J. Li, P. Liu, J. Mao, J. Yan, W. Song, *J. Mater. Chem. A* 2021, **9**, 1623-1629.

[11] S. Pang, W. Io, L. Wong, J. Zhao, J. Hao, *Adv. Sci*. 2020, **7**, 1903680.

[12] M. W. Glasscott, A. D. Pendergast, S. Goines, A. R. Bishop, A. T. Hoang, C. Renault, J. E. Dick, *Nat. Commun*. 2019, **10**, 2650.

[13] M. S. Hassan, P. Basera, S. Gahlawat, P. P. Ingole, S. Bhattacharya, S. Sapra, *J. Mater. Chem. A* 2021, **9**, 9837.

[14] K. Wu, L. Zhang, Y. Yuan, L. Zhong, Z. Chen, X. Chi, H. Lu, Z. Chen, R. Zou, T. Li, C. Jiang, Y. Chen, X. Peng, J. Lu, *Adv. Mater*. 2020, **32**, 2002292.

[15] S. G. Chandrappa, P. Moni, D. Chen, G. karkera, K. R. Prakasha, R. A. Caruso, A. S. Prakash, *J. Mater. Chem. A* 2020, **8**, 20612.

[16] Z. Wu, H. Wang, P. Xiong, G. Li, T. Qiu, W.-B. Gong, F. Zhao, C. Li, Q. Li, G. Wang, F. Geng, *Nano Lett*. 2020, **20**, 2892-2898.

[17] X. Han, N. Li, J. S. Baik, P. Xiong, Y. Kang, Q. Dou, Q. Liu, J. Y. Lee, C. S. Kim, H. S. Park, *Adv. Funct. Mater*. 2023, **33**, 2212233.

[18] Z. Li, W. Niu, Z. Yang, N. Zaman, W. Samarakoon, M. Wang, A. Kara, M. Lucero, M. V. Vyas, H. Cao, H. Zhou, G. E. Sterbinsky, Z. Feng, Y. Du, Y. Yang, *Energy Environ. Sci*. 2020, **13**, 884.

[19] J. Liu, C. Zhao, D. Ren, J. Wang, R. Zhang, S. Wang, C. Zhao, B. Li, Q. Zhang, *Adv. Mater*. 2022, **34**, 2109407.
